# A Practical Guide to Evidence Synthesis: Meta‐Analysis, Network Meta‐Analysis, Meta‐Regressions and Beyond

**DOI:** 10.1111/jre.70150

**Published:** 2026-07-25

**Authors:** Yu‐Kang Tu, Roberto Farina, Alessandra Viola, Tou‐Yuan Tsai, James S. Hodges

**Affiliations:** ^1^ Institute of Health Data Analytics & Statistics, College of Public Health National Taiwan University Taipei Taiwan; ^2^ Department of Dentistry, National Taiwan University Hospital National Taiwan University Taipei Taiwan; ^3^ Health Data Research Center National Taiwan University Taipei Taiwan; ^4^ Section of Dentistry University of Ferrara Ferrara Italy; ^5^ Institute of Epidemiology & Preventive Medicine, College of Public Health National Taiwan University Taipei Taiwan; ^6^ Center for Health Data & Advanced Translational Analytics, Dalin Tzu Chi Hospital, Buddhist Tzu Chi Medical Foundation Chiayi Taiwan; ^7^ School of Medicine Tzu Chi University Hualien Taiwan; ^8^ Division of Biostatistics and Health Data Science, School of Public Health University of Minnesota Minneapolis Minnesota USA

**Keywords:** consistency, exchangeability, meta‐analysis, network meta‐analysis, transitivity

## Abstract

Meta‐analysis has become an indispensable tool in medical and dental research, providing an estimate of treatment efficacy or harm by combining data from all relevant studies identified by a systematic review. Meta‐analysis produces evidence at the highest level of the study design hierarchy. Meta‐analysis is a broad term for a set of statistical tools used to analyse data across a range of research questions. Different research questions have different criteria for selecting relevant studies and extracting and organising data in different forms and require different statistical approaches to synthesising the data. This article focuses on meta‐analysis for comparing interventions. We use plain but precise language to explain the statistical concepts and assumptions underlying these methods. This article is divided into three parts. We begin with statistical models for and key assumptions of standard pairwise meta‐analysis, comparing two treatment groups. We then extend our discussion to network meta‐analysis, comparing multiple treatment groups. Finally, we discuss a few issues that arise in selecting studies and preparing data for a meta‐analysis. Throughout, we use examples from periodontal regeneration; all analyses were conducted using the free statistical software R. We strongly encourage close collaboration between periodontal researchers and experienced statisticians when planning and conducting meta‐analyses.

## Graphical abstract

This tutorial provides a clear and precise overview of pairwise and network meta‐analysis, including their core methodological assumptions.

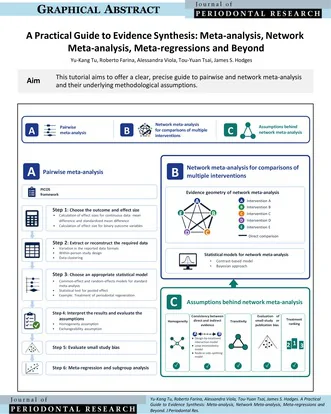

## 1 Introduction

Meta‐analysis has become an indispensable tool in medical and dental research, providing an overall estimate of treatment efficacy or harm by combining data from all relevant studies identified through a systematic review. Meta‐analysis is, therefore, an analysis of analyses, offering evidence of the highest level in the hierarchy of study designs [1, 2].

Meta‐analysis is a broad term for a set of statistical tools used to analyse data for a range of research questions including comparing the efficacy and safety of interventions [3], assessing the accuracy of diagnostic tests [4] and evaluating the dose–response relationship between exposure and disease risk [5]. Different research questions collect data in different forms and require different statistical approaches to synthesising the data.

Several articles provide overviews for dental researchers of conducting systematic reviews and meta‐analyses [6, 7, 8]. A few other articles assess the quality of published systematic reviews in oral health research using critical appraisal tools such as AMSTAR (A MeaSurement Tool to Assess systematic Reviews)‐2 and ROBIS (Risk of Bias Assessment Tool for Systematic Reviews) [9, 10, 11, 12, 13]. Systematic reviews in dental research are generally of low quality and at high risk of bias, and most of their results are not robust [12, 14, 15]. Those studies mainly assessed systematic reviews and meta‐analyses that compared two intervention groups, while more and more systematic reviews include network meta‐analysis (NMA) to compare multiple interventions [3, 16, 17, 18, 19]. It is very likely that the quality of NMA in dental research has room for significant improvement.

This article's aim is to provide periodontal researchers with a basic understanding of commonly used meta‐analytic methods, including both pairwise and network meta‐analyses for comparisons of interventions. The statistical concepts and assumptions underlying these methods are explained using plain language, emphasising issues specific to meta‐analyses in dental research.

This article has three parts. We begin with statistical models for and key assumptions of pairwise meta‐analysis, comparing two treatment groups. We then extend our discussion to network meta‐analysis, comparing multiple treatment groups. We also demonstrate how to conduct meta‐regression to identify effect modifiers. Finally, we discuss issues that arise in selecting studies and preparing data for a meta‐analysis. Throughout, we use examples from periodontal regeneration. All statistical analyses were conducted using the free statistical software R. The data and R scripts for the pairwise and network meta‐analysis are provided in the online Supporting Information S1 and R scripts. We also provide a list of common errors in conducting meta‐analysis and NMA in Table S1.

## 2 Pairwise Meta‐Analysis

To compare interventions, pairwise meta‐analysis synthesises results of trials comparing an active to a control treatment or comparing two active treatments. A systematic review is first conducted to identify relevant studies; data for meta‐analysis is then extracted from these studies. Because this article focuses on meta‐analysis, we only briefly mention the PICOS framework, which defines a systematic review's scope.

### 2.1 PICOS Framework

In a systematic review, the PICOS framework is often used to define the key components of a research question and eligibility criteria, ensuring transparency, reproducibility and clinical relevance:

*Population*: The target patient group defined by demographic, clinical and disease‐related characteristics (e.g., age, sex, disease definition, severity, setting).

*Intervention*: The treatment or strategy under evaluation, including type, dose, frequency and duration, as applicable.

*Comparator*: A reference treatment, standard care, placebo, or none, to which the intervention is compared.

*Outcomes*: Prespecified clinical, biochemical, functional, or patient‐reported measures used to quantify the intervention's effect versus the comparator's.

*Study design*: The types of study design eligible for inclusion in the review, such as randomised controlled trials, non‐randomised controlled studies, cohort studies, or case–control studies.

Information about the outcomes listed in the PICOS statement is extracted and organised, and quantitative data is processed for meta‐analysis. Effect size measures for the outcomes are then computed to compare the performance of different interventions in terms of efficacy and harm.

In the following section, we break the process of conducting a pairwise meta‐analysis into six steps:

Step 1. Choose the outcome and effect size.
Step 2. Extract or reconstruct the required data.
Step 3. Choose an appropriate statistical model.
Step 4. Interpret the results and evaluate the assumptions.
Step 5. Evaluate small study bias.
Step 6. Conduct subgroup analysis and meta‐regression to explore heterogeneity.

For demonstration, we use data from a recently published systematic review that identified 10 randomised controlled trials comparing guided tissue regeneration (GTR) with enamel matrix derivatives (EMD) to treat intrabony defects [20]. At each step, we first explain the concept and process, then use worked examples to illustrate. The primary outcome for our demonstration is reduction in probing pocket depth (PPD), a continuous variable measured in millimetres.

### 2.2 Step 1: Choose the Outcome and Effect Size

#### 2.2.1 Calculation of Effect Sizes for Continuous Data: Mean Difference

To conduct a meta‐analysis, an effect size is selected to measure the relative efficacy between two interventions. A statistical model is then used to summarise the effect sizes reported by the included studies, generally using a weighted mean. For continuous data, such as changes in probing depth, attachment level, or gingival recession, the mean value is typically used to measure treatment efficacy, and treatment comparisons of two groups are reported as differences in means. Meta‐analysis assigns a weight to the mean difference reported by each study to calculate a weighted mean difference.

For a study that randomly assigns patients to two treatment groups, A and B, the results in the two groups are considered independent. The effect size, mean difference, is calculated as $m_{A}-m_{B}$, where $m_{A}$ and $m_{B}$ are the means of groups A and B, respectively. For instance, average PPD reduction in the GTR and EMD groups in a study by Sculean et al. [21] are 4.5 and 4.3 mm, respectively. Thus, the mean difference is 4.5−4.3=0.2. The SE is calculated using this formula:

(1) $$\sigma =s_{p}\sqrt{\frac{1}{n_{A}}+\frac{1}{n_{B}}},$$

(2) $$\text{for}\;s_{p}^{2}=\frac{\left(n_{A}-1\right)s_{A}^{2}+\left(n_{B}-1\right)s_{B}^{2}}{n_{A}+n_{B}-2},$$

where $n_{A}$ and $n_{B}$ are the sample sizes and $s_{A}^{2}$ and $s_{B}^{2}$ are the variances in the GTR and EMD groups, respectively. A common mistake is confusing the standard error (SE) with the standard deviation (SD) when extracting data. SE is a measure of the uncertainty of the mean as an estimate of the population mean, while SD is a description of variation between individuals. For instance, when reporting the treatment effect of each intervention, we report the mean PPD reduction and its SD. A large SD suggests a wide variation in patients' responses to the treatment. For the difference in PPD reduction between two interventions, we report the mean difference and the SE of the mean. As the sample size increases, we have more information about the population mean, so the SE decreases. In contrast, SDs from different samples should be more or less similar, as they all estimate the population's SD. If one study's reported SD is much smaller than the other studies', it is likely in fact the SE. Statistical software packages for meta‐analysis usually allow users to enter the mean, sample size and SD for each group and to calculate each study's effect size internally before combining results across studies. Box 1 provides definitions of key statistical terms in this article.

BOX 1 **Definitions of statistical terms in this article.**

**Table:** 

Statistical terms	Definitions
SD (SD)	SD measures how far values in a dataset typically deviate from the mean. It is the square root of the variance.
SE of mean (SEM)	SEM measures how far a sample mean is likely to deviate from the true population mean, which is useful in making a statistical inference about the population mean. $\mathrm{SEM}=\frac{\mathrm{SD}}{\sqrt{n}}$, so SEM decreases when the sample size n increases.
Within‐person design	An experimental method in which the same participants are exposed to all interventions of a study. Participants serve as their own controls, allowing researchers to directly compare performance across conditions, reduce individual variation, use smaller sample sizes and increase statistical power.
Intraclass correlation (ICC)	ICC is the ratio of between‐cluster variance to total variance. It measures the reliability of measurements or the degree of similarity within clusters.
Heterogeneity	Variation in reported results across the included studies in a pairwise meta‐analysis or across studies comparing the same interventions in a network meta‐analysis.
Exchangeability	In a meta‐analysis, relative treatment effects are exchangeable when the included studies have a similar distribution of effect modifiers.

#### 2.2.2 Calculation of Effect Sizes for Continuous Data: Standardised Mean Difference

Some patient‐reported outcomes are measured using different tools, such as questionnaires or visual scales. Because these tools may have different ranges of possible values, they need to be standardised before they can be combined in a weighted mean difference. Three common methods to calculate standardised mean differences are Cohen's d, Hedges' g and Glass's Δ:

$${\text{Cohen}}^{\prime }s\;d_{i}=\frac{m_{\mathrm{Ai}}-m_{\mathrm{Bi}}}{s_{p_{i}}},$$

where $m_{\mathrm{Ai}}$ and $m_{\mathrm{Bi}}$ are the means of group A and group B in study i respectively and $s_{p_{i}}$ is the squared root of the pooled variance, $s_{p}^{2}$, in Equation (2). Hedges' g is a modification of Cohen's d intended to correct for potential bias in Cohen's d in small sample sizes:

$${\text{Hedges}}^{\prime }s\;g_{i}=\frac{m_{\mathrm{Ai}}-m_{\mathrm{Bi}}}{s_{p_{i}}}\left(1-\frac{3}{4N_{i}-9}\right),$$

where $N_{i}=n_{\mathrm{Ai}}+n_{\mathrm{Bi}}$, the total sample size of the two groups in study i. When $N_{i}$ is large, Hedges' g is very close to Cohen's d. Glass's Δ differs from Cohen's d in the denominator:

$${\text{Glass}}^{\prime }s\;\Delta_{i}=\frac{m_{\mathrm{Ai}}-m_{\mathrm{Bi}}}{s_{\mathrm{Bi}}},$$

where $s_{\mathrm{Bi}}$ is the SD of the group B, which is taken to be a control group. If several treatments are compared to the control group, it might be better to use the SD computed only from the control group, so that effect sizes do not differ if the groups have different variances [22]. This is especially relevant in a network meta‐analysis that includes multi‐arm trials. Unless a pairwise meta‐analysis includes many studies with small sample sizes and the SDs substantially differ between the experimental and control groups, the three methods for standardisation will yield similar results.

#### 2.2.3 Calculation of Effect Size for Binary Outcome Variables

Binary outcomes, such as treatment success or failure, are often reported in a 2‐by‐2 table:

**Table:** 

	Success	Failure	
Treatment A	$n_{1A}$	$n_{0A}$	$N_{A}=n_{1A}+n_{0A}$
Treatment B	$n_{1B}$	$n_{0B}$	$N_{B}=n_{1B}+n_{0B}$
	$N_{1}=n_{1A}+n_{1B}$	$N_{0}=n_{0A}+n_{0B}$	$N=N_{1}+N_{0}=N_{A}+N_{B}$

where $n_{\mathrm{jk}}$ is the number of teeth or patients in each cell of the table. Odds ratios (OR) and risk ratios (RR) are often used to measure the relative efficacy between two interventions:

OR=n1An0An1Bn0BandRR=n1ANAn1BNB.

OR or RR reported by different studies can be combined in different ways to estimate an average effect. One way is to transform the OR or RR to a continuous variable that approximately follows a normal distribution: for example, transforming the OR to the natural log OR (logOR):

logOR=logn1An0An1Bn0B=logn1An0A−logn1Bn0B,

or transforming a RR to a natural log RR (logRR):

logRR=logn1ANAn1BNB=logn1ANA−logn1BNB.

The approximate variance of logOR, used to calculate weights for a meta‐analysis, is:

$$\mathrm{var}\left(\text{logOR}\right)=\frac{1}{n_{1A}}+\frac{1}{n_{0A}}+\frac{1}{n_{1B}}+\frac{1}{n_{0B}};$$

and the approximate variance of logRR is:

$$\mathrm{var}\left(\text{logRR}\right)=\frac{1}{n_{1A}}-\frac{1}{N_{A}}+\frac{1}{n_{1B}}-\frac{1}{N_{B}},$$

as long as none of the denominators is zero. If $n_{1A}$ or $n_{1B}$ is 0 (very low success rate) or $n_{0A}$ or $n_{0B}$ is 0 (very high success rate), a simple solution is to add 0.5 to each of the four cells in the 2‐by‐2 table and then apply the formulas. If both $n_{1A}$ and $n_{1B}$ are 0 or both $n_{0A}$ and $n_{0B}$ are 0, such a study is usually excluded from a meta‐analysis because it provides no information about the difference in treatment efficacy between A and B. The inverse‐variance methods for the common‐effect and random‐effects models (described below) can be used to synthesise data to obtain a weighted mean of logOR or logRR and a confidence interval. These can then be transformed back to OR and RR by taking their exponentials. When success or failure rates in many studies are extremely low or high, this approach may artificially add too many non‐existent cases in the data, leading to biased results. Alternative methods for binary data meta‐analysis, such as Mantel–Haenszel's method or Peto's odds ratio, can address some of these problems, but both are common‐effect models and are rarely used to compare multiple interventions. Readers can consult standard textbooks for details [23, 24]. Box 2 compares continuous and dichotomous variables according to the characteristics of effect sizes.

BOX 2 **Effect size measures for continuous versus dichotomous outcomes.**

**Table:** 

Feature	Continuous outcomes	Dichotomous outcomes
Typical data	Mean, SD, sample size	Number of events, total sample size
Common effect measures	Mean Difference (MD) Standardised Mean Difference (SMD)	Risk Difference (RD) Risk Ratio (RR) Odds Ratio (OR)
Definition	Difference in group means	Comparison of event probabilities
Formula (basic)	$\mathrm{MD}=m_{A}-m_{B}$ $\mathrm{SMD}=\frac{m_{A}-m_{B}}{s_{p}}$	$\mathrm{RD}=p_{1}-p_{0}$ $\mathrm{RR}=\frac{p_{1}}{p_{0}}$ $\text{OR}=\frac{\frac{p_{1}}{1-p_{1}}}{\frac{p_{0}}{1-p_{0}}}$
Scale dependence	MD depends on measurement scale SMD is scale‐free	RD is absolute difference in risk RR/OR are relative measures
When to use	MD: same measurement scale in all studies SMD: different scales measuring same construct	RD: clinically intuitive RR: common in cohort/RCTs OR: case–control studies, logistic regression
Statistical properties	Usually approximately normal (large samples)	Often analysed on log scale (RR, OR)
Common transformation for meta‐analysis	SMD may require small‐sample correction (Hedges' g)	log(RR), log(OR) used for analysis

### 2.3 Step 2. Extract or Reconstruct the Required Data

#### 2.3.1 Variation in the Reported Data Formats

For continuous outcomes, data may be presented in different formats or some information may be missing. If changes in PPD are not reported, for each treatment arm we can use the mean baseline ($\mathrm{PP}D_{0}$) and follow‐up PPD ($\mathrm{PP}D_{1}$) values, along with their SDs, to calculate the mean PPD reduction and its SD. The mean PPD reduction (ΔPPD) is the difference between the mean baseline and follow‐up PPD values: $\Delta \mathrm{PPD}=P{\mathrm{PD}}_{0}-\mathrm{PP}D_{1}$ and its SD ($sd_{\Delta \mathrm{PPD}}$) is given by this formula:

$$sd_{\Delta \mathrm{PPD}}=\sqrt{sd_{\mathrm{PP}D_{0}}^{2}+sd_{\mathrm{PP}D_{1}}^{2}-r_{01}sd_{\mathrm{PP}D_{0}}sd_{\mathrm{PP}D_{1}}},$$

where $sd_{\mathrm{PP}D_{0}}$ and $sd_{\mathrm{PP}D_{1}}$ are the SDs of baseline and follow‐up PPD respectively and $r_{01}$ is their Pearson's correlation. Usually, $r_{01}$ is not reported; instead, a value of 0.5 is often used because baseline and follow‐up PPD are repeated measurements of the same variable and expected to be positively correlated. We may also obtain $r_{01}$ from other studies that reported SDs for baseline and follow‐up PPD and change in PPD, and use the average or the highest and lowest reported value [25].

If a trial reports only the mean follow‐up PPD and SD, the difference in the mean follow‐up PPD between the two groups can be used as ΔPPD by assuming that the mean baseline PPD of the two groups is approximately equal due to randomisation, in which case the group difference in PPD reduction is the difference between the mean follow‐up PPDs. When the baseline and follow‐up SDs are equal, the variance of the change in PPD equals the variance of the follow‐up PPD if the correlation between baseline and follow‐up PPD, $r_{01}$, is 0.5. Under this condition, using follow‐up PPD has the same statistical precision as using change in PPD [26, 27].

On rare occasions, a study may report only the mean difference in PPD reduction between two groups and the *p*‐value from a statistical test, such as a two‐sample *t*‐test. We can obtain the SE for the mean difference by assuming the ratio of the mean to the SE follows a *t* distribution. For instance, suppose a study reported a difference of 2.6 mm in PPD reduction between GTR and EMD, with p<0.001 from a *t*‐test. The corresponding t‐value for the *p*‐value is derived from:

$$t=\frac{\theta }{\sigma_{\theta }}=\frac{2.6}{\sigma_{\theta }},$$

where θ is the mean difference in PPD reduction and σ is its SE. We can obtain a critical value for t using the cumulative t‐distribution function in statistical software. For two‐sided significance level 0.001 and 38 degrees of freedom (N=40), the critical value corresponds to a cumulative probability of 0.9995 (i.e., upper‐tail probability 0.0005), giving t=3.566. Thus, we impute the SE for $\left(\sigma_{\theta }\right)$ as $\frac{2.6}{3.566}=0.73$, which is larger than the true SE because the exact *p*‐value is below 0.001.

#### 2.3.2 Within‐Person Study Design

The above formulas assume that the observations are independent, for example, that each patient contributes only one intrabony defect. This assumption is violated when a trial uses a within‐person study design, such as a split‐mouth or crossover design. Patients' responses to a second treatment may be affected by the previous treatment, known as a carry‐over effect, so the results of the two treatments within the same patients cannot be considered independent [28]. For instance, suppose two teeth from each of 12 patients were randomly assigned to either GTR or EMD, or patients were randomly assigned to use a manual toothbrush first and an electric toothbrush second, or *vice versa*. A correct analysis calculates the difference in PPD reduction for the pair of teeth within a patient, then uses a paired *t*‐test to assess whether the average difference across the 12 pairs differs from zero. When a meta‐analysis includes studies with both within‐person and parallel‐group designs, a straightforward approach is to extract from each study the mean difference in treatment outcomes between two groups and its SE and provide those to a statistical software package. Sometimes studies with a within‐person design report only the mean treatment outcomes for each group. A simple solution is to use a within‐person correlation $r_{\mathrm{AB}}$ in calculating the variance of the mean difference:

$$\sigma_{\theta }^{2}=\sigma_{A}^{2}+\sigma_{B}^{2}-2r_{\mathrm{AB}}\sigma_{A}\sigma_{B},$$

where $\sigma_{\theta }^{2}$ is the variance of the difference in PPD reduction between the GTR and EMD, and $\sigma_{A}^{2}$ and $\sigma_{B}^{2}$ are the variances of the mean PPD reduction for GTR and EMD, respectively. The within‐person correlation between two teeth $r_{\mathrm{AB}}$ is usually not reported, and it has been suggested that a positive value of 0.25 be used instead [29].

For some software packages, it is more convenient to provide data on individual treatment groups within each study rather than on the difference between a study's two groups. This is especially true for network meta‐analysis, where some studies have more than two treatment groups. We then need to adjust the data if Equations (1) and (2) are used to calculate the variance of the mean difference between the two groups. For instance, the original SDs reported by Sculean et al. [21] are 1.3 and 1.2 mm for the GTR and EMD groups, respectively, while the adjusted SDs are 1.14 and 1.02 mm, respectively. For NMA, we refer readers to our earlier paper for guidance on making adjustments to data from studies using a within‐person design to compare more than two interventions [30].

#### 2.3.3 Data Clustering

Sometimes, more than one tooth from a patient receives the same treatment in a trial, and teeth within the same patient cannot be assumed to be independent. This violates the assumption of independently and identically distributed data underlying most classic statistical tests, such as the *t*‐test, ANOVA and regression. For instance, suppose each of 10 patients contributes three teeth in a clinical trial comparing two surgical root coverage techniques. If teeth in the same patient are considered independent, a tooth's response to treatment is considered no more similar to the response to treatment of another tooth in the same patient than to the response of a tooth in a different patient. Therefore, it is as if the 60 teeth from 20 patients came from 60 different patients. This assumption is usually unrealistic because responses of teeth within the same patient are affected by effect modifiers that pertain to the patient, such as smoking. However, teeth within the same patient differ in their characteristics at the tooth level, so they are unlikely to show identical responses to treatment.

This type of data is known as multilevel or hierarchical data, and advanced statistical methods, such as multilevel models or generalised estimating equations, are required to account for the correlation of data within the same patient. A meta‐analysis should use the SE of the treatment outcome obtained from these methods. If a trial did not use these methods in its original analysis and reported only the SD of the data and the number of teeth and patients, adjustments need to be made to the reported SE.

The Cochrane Handbook describes an approach to adjusting for correlation between data within the same patient by calculating the effective sample sizes [25], which ranges from the number of patients (the minimum) to the number of teeth (the maximum). This approach first calculates the design effect (DE) using the following approximate formula:

$$\mathrm{DE}=1+\left(M-1\right)\times \mathrm{ICC},$$

where M is the average cluster size and ICC is the intraclass correlation coefficient. For instance, if each patient contributes three teeth, M = 3. ICC is the ratio of between‐cluster variance to total variance:

$$\mathrm{ICC}=\frac{s_{B}^{2}}{{s_{B}^{2}+s}_{w}^{2}},$$

where $s_{B}^{2}$ is between‐cluster (i.e., between‐patient in our example) variance, and $s_{W}^{2}$ is within‐cluster variance. The total number of teeth is then divided by DE to obtain the effective sample size. For instance, in our hypothetical example, each of the 10 patients in the treatment or the control group contributes 3 teeth to the trial. Suppose ICC is 0.5, so DE is $1+\left(3-1\right)\times 0.5=2$. The effective sample size of each group is, therefore, $\frac{30}{2}=15$. When teeth within the same patient are completely independent, ICC is 0, the effective sample size is the number of teeth. When teeth within the same patients have a correlation of 1, ICC is 1, the effective sample size is the number of patients.

Calculating ICC requires access to individual data of the original trial, which is usually unavailable. One solution is to contact authors to request original data. ICC derived from studies in which original data are made available is then applied to those for which original data are unavailable [3]. If the original data are not available from any study, a pragmatic though conservative approach is to use the number of patients as the effective sample size, which is equivalent to assuming an ICC of 1.

### 2.4 Step 3. Choose an Appropriate Statistical Model

#### 2.4.1 Common‐Effect and Random‐Effects Models for Standard meta‐Analysis

For a pairwise meta‐analysis comparing treatment efficacy between GTR and EMD, the effect size measure for a study is the mean difference in PPD reduction between the two groups in that study. For study i, the mean difference (MD, denoted as ${\hat{\theta }}_{i}$) and its SE (denoted as $\sigma_{i}$) reported by each trial is summarised across studies i as the pooled mean difference in PPD reduction:

$$\begin{array}{c} \;\hat{\theta }=\frac{\sum w_{i}{\hat{\theta }}_{i}}{\sum w_{i}},\;\text{Model}\;\left(1\right) \end{array}$$

where $w_{i}=\frac{1}{\sigma_{i}^{2}}$ is the weight assigned to study i, which is inversely related to $\sigma_{i}^{2}$, the squared SE of ${\hat{\theta }}_{i}$. Thus, a study reporting a more precise estimate ${\hat{\theta }}_{i}$, that is, a smaller SE or a shorter confidence interval (the confidence interval's width is a multiple of the SE) receives more weight than a study reporting a less precise estimate ${\hat{\theta }}_{i}$. Model (1) is known as the *common‐effect model*, also called the fixed‐effect model. This model assumes that the true unobserved difference in PPD reduction between GTR and EMD is the same for all studies, even though the *observed* differences in PPD reduction vary due to sampling variation. The pooled mean difference, $\hat{\theta }$ given in Model (1), is the ‘best’ guess of that true unobserved difference, θ. Because the SE of the mean of a continuous variable is inversely related to the square root of the sample size ($\sigma =\frac{\mathrm{SD}}{\sqrt{n}}$, where n is the sample size), a study with a larger number of patients will, therefore, have a greater weight in a meta‐analysis.

The *random‐effects model*, by contrast, assumes study $i^{\prime }s$ true effect size $\theta_{i}$ is a random variable drawn from a normal distribution with mean θ and variance $\tau^{2}$, so it varies from study to study. For example, a τ^2^ of 0.5 corresponds to a SD of approximately 0.7 mm, meaning that the true treatment effects typically vary across studies by about 0.7 mm around the overall pooled effect. If the treatment effects ${\hat{\theta }}_{i}$ estimated in different studies vary considerably, the random‐effects model's assumption of varying true effects is likely more realistic than the common‐effect model's assumption of a fixed true effect. The random‐effects model uses a different weight in Model (1), with $w_{i}=\frac{1}{\sigma_{i}^{2}+{\hat{\tau }}^{2}}$, where ${\hat{\tau }}^{2}$ is an estimate of the between‐study heterogeneity $\tau^{2}$ in the treatment effect. There are several methods for estimating $\tau^{2}$, for example, the popular DerSimonian‐Laird method and the restricted maximum likelihood method. Because a constant ${\hat{\tau }}^{2}$ is added to the denominator of $w_{i}$, studies with larger weight due to a smaller SE, $\sigma_{i}^{2}$, are assigned a relatively smaller weight in the random‐effects model than in the common‐effect model. Box 3 summarises the assumptions of the common‐effect and random‐effects models.

BOX 3 **Summary of the assumptions and applications of common‐effect and random‐effects models.**

**Table:** 

	Common (fixed) effect model	Random‐effects model
Assumptions	The unobserved true treatment effect, θ, is a fixed value in the patient population from which patients in the included studies were recruited.	The unobserved true treatment effect, $\theta_{i}$, is a random variable in the patient population from which patients in the included studies were recruited. Usually, $\theta_{i}$ is assumed to follow a normal distribution.
Applications	Choose the common‐effect model when effect modifiers have little or no impact on treatment effects.	Choose the random‐effects model when effect modifiers lead to differences in treatment effects across studies. Differences in patient characteristics (effect modifiers) are a common reason why treatment effects vary across studies.

#### 2.4.2 Statistical Test for Pooled Effect

In a meta‐analysis, the statistical significance of the pooled effect is commonly assessed using a Z‐test. Once the pooled estimate $\hat{\theta }$ and its SE, $\mathrm{SE}\left(\hat{\theta }\right)$, have been obtained (under either a common‐effect or random‐effects model), the test statistic is calculated as:

$$Z=\frac{\hat{\theta }}{\mathrm{SE}\left(\hat{\theta }\right)}.$$

Under the null hypothesis of no effect (θ=0), the Z statistic approximately follows a standard normal distribution with zero mean and variance 1. A two‐sided *p*‐value can then be obtained by comparing Z to the standard normal distribution. When Z>1.96 or Z<−1.96, the null hypothesis of no effect is rejected. A 95% confidence interval for the pooled estimate is given by

$$\hat{\theta }\pm 1.96\times \mathrm{SE}\left(\hat{\theta }\right),$$

and statistical significance at the 5% level is achieved if this interval does not include the null value (e.g., 0 for mean differences or log odds ratios, and 1 for odds ratios or risk ratios). Thus, the Z‐test and confidence interval provide equivalent information, expressed in different forms.

#### 2.4.3 Example: Pairwise meta‐Analysis of Randomised Controlled Trials Comparing GTR With EMD

In our example, 10 randomised controlled trials comparing GTR with EMD were included in the meta‐analysis. Figure 1 is a forest plot, showing the mean difference in PPD reduction between GTR and EMD reported by each study and the weighted mean differences from the common‐effect and random‐effects models.

**FIGURE 1:**
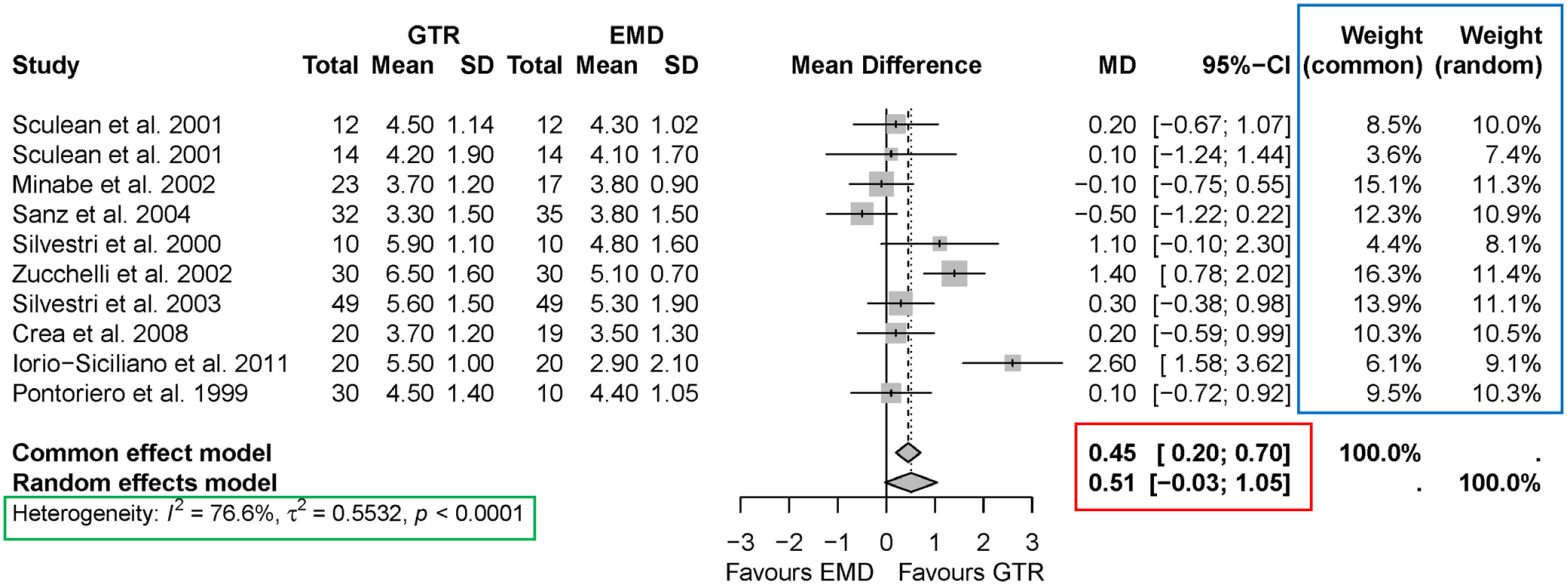
Forest plot for PPD reduction in the meta‐analysis comparing GTR with EMD. Columns on the left show the study labels (Study), including the name of the first author and publication year, and for each of the GTR and EMD groups, the number of patients (Total), the mean PPD reduction (Mean), and the SD (SD). The red box on the bottom right shows the mean differences in PPD reduction and their associated confidence intervals estimated by the common‐effect and random‐effects models; the blue box shows the weights assigned to each study by the two models. The solid line in the middle of the plot is the null effect, indicating no difference in PPD reduction between GTR and EMD. The dashed line and dotted line on the right show the weighted mean differences estimated by the common‐effect and random‐effects models, respectively. The green box on the bottom left shows heterogeneity statistics such as $I^{2}$ and $\tau^{2}$, discussed below.

For each trial, the mean difference in PPD reduction is shown as a tick and its confidence interval is shown as a horizontal line. The size of the grey box surrounding the tick is proportional to the weight assigned to each trial in the common‐effect model. The common‐effect weights do not differ greatly between the 10 trials, and the random‐effects model's weights differ even less. The two diamonds at the bottom represent the pooled estimates and confidence intervals obtained from the common‐effect and random‐effects models. The solid line in the middle is the null effect, indicating no difference in PPD reduction between GTR and EMD. The dashed line and dotted line on the right show the weighted mean differences estimated by the common‐effect and random‐effects models, respectively. Both indicate that GTR achieves around 0.5 mm greater PPD reduction than EMD, but the difference is not statistically significant in the random‐effects model due to a wider confidence interval.

As PPD was measured and reported on the same scale in all included studies, the mean difference is generally the preferred effect measure. Using the standardised mean difference and the random‐effects model yields a pooled effect estimate of 0.35 (95% CI: −0.02 to 0.71). Although this does not alter the substantive conclusion, the standardised mean difference is less interpretable because it removes the original measurement unit and expresses the treatment effect in standard deviation units.

### 2.5 Step 4: Interpret the Results and Evaluate the Assumptions

#### 2.5.1 Homogeneity Assumption

For a meaningful interpretation of the pooled overall treatment effect estimate, the standard meta‐analysis requires that the included studies be comparable. Study comparability may be attained by using strict inclusion criteria in the systematic review. for instance, only studies that (1) excluded smokers, (2) included patients with intrabony defects deeper than 4 mm and (3) provided intensive maintenance care are included. This would reduce variation in study populations and protocols and ensure that included studies are more comparable in their distributions of effect modifiers. The reported results of the included studies may thus be more homogeneous, allowing a straightforward interpretation of the pooled estimate. In this scenario, between‐study heterogeneity in the reported treatment effects, $\tau^{2}$, is expected to be small and attributable to sampling variation within studies, so the common‐effect and random‐effects models yield similar results.

However, few trials would meet such strict inclusion criteria, and the results would apply only to patient populations with characteristics similar to those in the included studies, that is, non‐smokers with intrabony defect deeper than 4 mm receiving intensive maintenance care. Additional criteria could be added to further improve comparability; then, even fewer studies would be included, and conducting a meta‐analysis would become unnecessary or impossible.

Alternatively, we can relax the selection criteria to include more studies in the meta‐analysis and investigate variation in the reported treatment effects. Between‐study heterogeneity in Figure 1's forest plot, $\tau^{2}$, is estimated to be 0.55 by the DerSimonian‐Laird method. Its square root is 0.74 mm, which can be roughly interpreted as the SD of the distribution of true differences between GTR and EMD ($\theta_{i}$) across the trials. The *p*‐value <0.0001 is from Cochran's Q‐test for heterogeneity:

(3) $$Q=\sum_{i=1}^{k}w_{i}{\left({\hat{\theta }}_{i}-\hat{\theta }\right)}^{2}=\sum_{i=1}^{k}\frac{{\left({\hat{\theta }}_{i}-\hat{\theta }\right)}^{2}}{\sigma_{i}^{2}},$$

where k is the number of studies. A small *p*‐value suggests that observed heterogeneity is greater than expected from sampling variability within studies. Q in Equation (3) can be interpreted as the sum of the squared differences between the observed treatment effects and the average treatment effect, standardised by the SE of the observed treatment effect. If two studies report the same ${\hat{\theta }}_{i}$, the one with smaller $\sigma_{i}$ contributes more to the sum in Equation (3) than the one with larger $\sigma_{i}$.

To illustrate how *Q* is calculated, consider three studies with these estimated effects and SEs:

**Table:** 

Study	Effect ($\hat{\theta_{i}}$)	SE ($\sigma_{i}$)	Weight $\left(w_{i}=\frac{1}{\;\sigma_{i}^{2}}\right)$	$w_{i}{\left({\hat{\theta }}_{i}-\hat{\theta }\right)}^{2}$
1	0.2	0.1	100	$100\;{\left(0.20-0.189\right)}^{2}=0.0121$
2	0.5	0.2	25	$25\;{\left(0.50-0.189\right)}^{2}=2.418$
3	0.1	0.1	100	$100\;{\left(0.10-0.189\right)}^{2}=0.7921$

Under a common‐effect model, each study is weighted by the inverse of its variance, as shown in the Weight column. Then we calculate the pooled effect: $\hat{\theta }=\frac{\sum w_{i}{\hat{\theta }}_{i}}{\sum w_{i}}=\frac{100\times 0.2+25\times 0.5+100\times 0.1}{100+25+100}=0.189$. The next step is to calculate weighted squared deviations, $w_{i}{\left({\hat{\theta }}_{i}-\hat{\theta }\right)}^{2}$, as shown in the rightmost column. The Q statistic is the sum of the weighted squared deviations: Q=0.0121+2.418+0.7921=3.22. Q=3.22 is then compared to a Chi‐square distribution with 2 degrees of freedom, which is the number of studies minus 1. Since Q is close to the degrees of freedom, this suggests little evidence of heterogeneity in this example.

$I^{2}$ is another popular index for evaluating between‐study heterogeneity [31]. $I^{2}$ is defined as:

$$I^{2}=\left(\frac{Q-\mathrm{df}}{Q}\right)\times 100\%,$$

where Q is Cochran's Q and df is the degrees of freedom, k−1, which is the expected contribution to Q of sampling variation for a meta‐analysis with k studies. Under the common‐effect model assumption that the true effect is the same for all studies, the observed effects of individual studies may still vary because, for example, they recruited different patient samples. The expected variation is k−1, so $I^{2}$ measures the contribution of the additional variation to total variation, ranging from 0% to 100%. There is a common misconception that $I^{2}=0$ indicates no heterogeneity at all. The correct interpretation is that observed heterogeneity is less than what would be expected from random variation. $I^{2}$ between 30% and 60% is considered moderate heterogeneity; $I^{2}$ > 60% is deemed substantial or considerable heterogeneity [23]. The importance and interpretation of $I^{2}$ depend on the magnitude and direction of the treatment effects. For example, $I^{2}=76.6\%$ in Figure 1 is quite large, indicating substantial heterogeneity in the reported differences in PPD reduction between GTR and EMD. Both common‐effect and random‐effects models indicate that GTR achieves approximately 0.5 mm greater PPD reduction than EMD; however, GTR did not consistently outperform EMD, as it achieved a nonsignificantly smaller PPD reduction than EMD in two of the 10 trials in the meta‐analysis. Therefore, we need to be cautious in interpreting the better treatment effects of GTR.

Choosing the common‐effect or random‐effects model using any rule based on Cochran's Q test or $I^{2}$ is not recommended. As explained in the previous section, these two models make different assumptions about the true treatment effects, and Cochran's Q test and $I^{2}$ are affected by several factors. For instance, when the number of included studies is small, both Cochran's Q and $I^{2}$ are unreliable [32, 33]. Researchers should instead consider which model best suits their research questions. For example, because the GTR group in our meta‐analysis comprises different types of barrier membranes and surgical techniques, the assumption of a fixed true effect seems unrealistic. Therefore, we will focus on results from the random‐effects model. From a statistical perspective, the random‐effects and the common‐effect models yield similar results when the heterogeneity ($\tau^{2}$) is small, as both models assign similar weights to each study.

#### 2.5.2 Exchangeability Assumption

Standard meta‐analysis combines the differences between two interventions in treatment outcomes within each study, known as treatment contrasts, yielding a pooled mean difference. It does not combine the absolute effects of each treatment across studies and then compare the difference in mean effects between two treatments. This is because standard meta‐analysis assumes that treatment contrasts are exchangeable and therefore comparable across studies, whereas the absolute effects of each treatment are usually not assumed to be either exchangeable or comparable.

Take the meta‐analysis comparing GTR with EMD as an example: although the included studies met the eligibility criteria, they differed in baseline characteristics of the treated teeth and patients, as well as in administration of the same treatment regimens. Intrabony defect depths might be greater in some studies, while investigators in other studies may have more experience and greater skill. Since only randomised trials were included, the factors or effect modifiers that potentially affect treatment outcomes are considered balanced within each trial, so the difference in PPD reduction between GTR and EMD reported by each trial is an unbiased estimate of the true value for that trial's protocol and study population. In contrast, the absolute treatment effects, that is, mean PPD reduction of GTR or EMD, are not exchangeable or comparable across trials, so a weighted mean across studies of absolute treatment effects lacks a clear interpretation. We discuss the exchangeability assumption further in the sections on network meta‐analysis, as it plays a crucial role in the validity of the results.

Some effect modifiers may modify the relative effect of GTR versus EMD. If those effect modifiers are distributed differently in different included studies, a substantial heterogeneity in the reported mean differences in PPD reduction may be observed. Subgroup analysis and meta‐regression can then be used to identify those relative effect modifiers.

In summary, meta‐analysis assumes no relative effect modifiers; therefore, treatment contrasts are comparable across studies and can be pooled together to yield an average effect.

### 2.6 Step 5: Evaluate Small Study bias

The common‐effect and random‐effects models should yield similar estimates of θ in Model (1) despite different weight assignments. If the two models give substantially different results, this suggests small‐study bias or publication bias, which arise from a correlation between effect size and sample size. Recall that the SE of the mean is inversely related to the square root of the sample size, so studies with small sample sizes tend to have greater variation in the observed effect sizes. Some small studies may produce large effect sizes by chance and therefore be more likely to be published than studies giving non‐significant or null effects. The most popular method to evaluate this correlation is Egger's test, which assumes a linear relation between effect size, ${\hat{\theta }}_{i}$, reported by study i and its SE, $\sigma_{i}$, in a regression model:

$${\hat{\theta }}_{i}=b_{0}+b_{1}\sigma_{i}+\epsilon_{i},\;\text{Model}\;\left(2\right)$$

where the intercept, $b_{0}$, is the expected ${\hat{\theta }}_{i}$ when $\sigma_{i}$ is zero. It represents the estimated treatment effect we would expect from an infinitely large, perfectly precise study (i.e., in which random error is essentially zero). As no such study exists, this interpretation of $b_{0}$ is an extrapolation. The slope, $b_{1}$, describes how the estimated treatment effect changes as studies become less precise (i.e., larger $\sigma_{i}$). If the slope is zero, results are similar across small and large studies. If the slope is non‐zero, smaller studies tend to report systematically different results (often larger effects), which may indicate publication or small‐study bias. Dividing the equation by $\sigma_{i}$, we obtain Egger's regression model:

$$\frac{{\hat{\theta }}_{i}}{\sigma_{i}}=b_{0}\frac{1}{\sigma_{i}}+b_{1}+\epsilon_{i},\;\text{Model}\;\left(3\right)$$

where the regression coefficient for $\sigma_{i}$ in Model (2), $b_{1}$, becomes the intercept in Model (3), that is, the expected standardised effect, $\frac{{\hat{\theta }}_{i}}{\sigma_{i}}$, when $\sigma_{i}$ approaches infinity. Therefore, if Model (3)'s intercept deviates from zero significantly, it indicates a relationship between effect sizes and SEs. Because SEs are inversely related to sample sizes, a significant intercept in Egger's regression suggests that effect sizes are related to sample sizes. Figure 2 is a scatter plot of the results from Egger's regression for the meta‐analysis comparing GTR and EMD. The vertical axis is the standardised effect, $\frac{{\hat{\theta }}_{i}}{\sigma_{i}}$, and the horizontal axis is the inverse of the SE, $\frac{1}{\sigma_{i}}$. Each circle represents a study. The blue line is the null hypothesis of a zero intercept, $b_{1}$, in Model (3), while the red line is the confidence interval for the intercept. As the red line crosses the blue line, we cannot reject the null hypothesis that the intercept is zero; we conclude that this test provides no evidence of small study bias.

**FIGURE 2:**
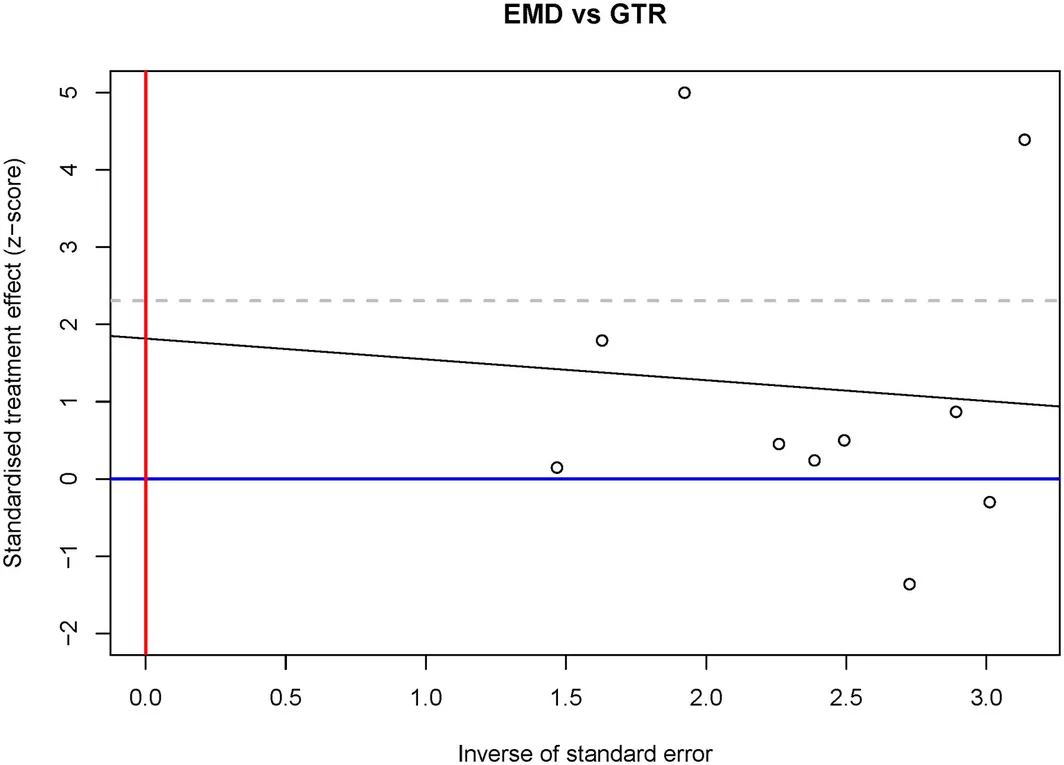
A scatter plot for Egger's regression test.

Another common approach to evaluating small study bias is a funnel plot, which plots the SE ($\sigma_{i}$) against the estimated effect size (${\hat{\theta }}_{i}$). Figure 3 is a contour‐enhanced funnel plot, in which a filled circle represents a study. The vertical axis is reversed, so studies with larger sample sizes and smaller SEs (i.e., smaller sampling variation in their estimated effect sizes) are shown at the top while smaller, less precise studies are shown at the bottom. In the absence of publication bias, the studies will spread out in a symmetrical funnel shape around the pooled estimate, with variability due to sampling error increasing down the y‐axis. The funnel's centre is a vertical dotted line at a mean difference of 0.45 mm, which is the weighted mean difference in PPD reduction between GTR and EMD, estimated by the common‐effect model. The two sloped dotted lines are the confidence limits of the weighted mean difference. The 10 filled dots are expected to be distributed around the centre of the funnel symmetrically if there is no publication bias. Since EMD entered the market later than GTR, a greater number of small studies in the bottom‐right part of the funnel would be evidence of publication bias favouring EMD, which is not the case in Figure 3.

**FIGURE 3:**
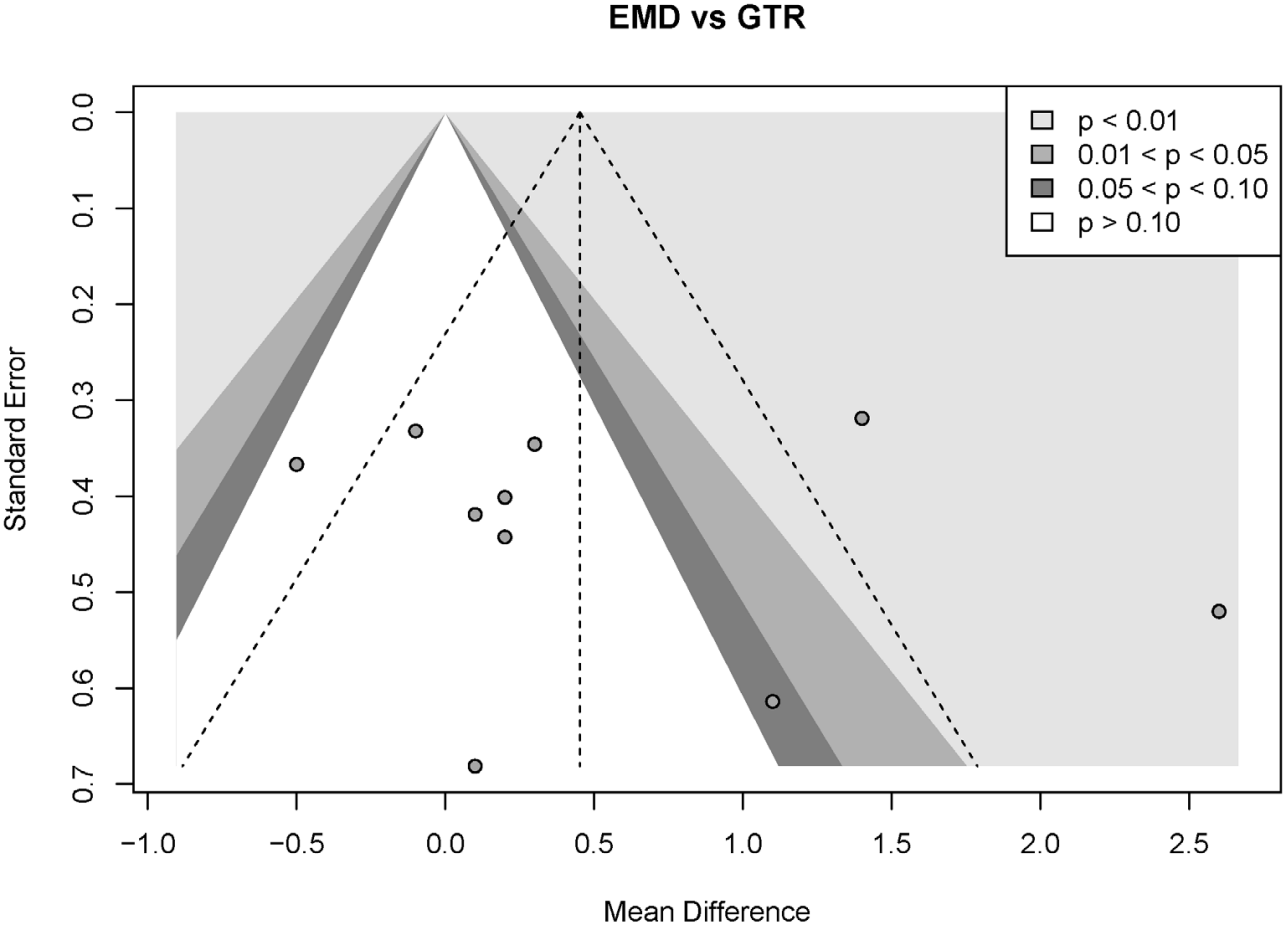
Funnel plot for the meta‐analysis comparing GTR with EMD.

To enhance interpretation, Figure 3 is coloured in different shades of grey under a hypothetical scenario in which there is no difference in PPD reduction between GTR and EMD. Small studies tend to have larger SEs, so they are located at the bottom of the plot; they need to achieve larger effect sizes to obtain smaller *p*‐values. If more small studies favouring one treatment appear in the parts of the funnel plot with small *p*‐values, this suggests publication bias. For example, if many small studies are found in the shaded areas in the bottom‐right of Figure 3, this suggests that small studies reporting significantly better effects of GTR compared to EMD are more likely to be published.

### 2.7 Step 6: Meta‐Regression and Subgroup Analysis

When between‐study heterogeneity is large, the random‐effects model is not the ultimate solution. A better course is to try to identify sources of heterogeneity. Meta‐regression investigates relationships between reported relative treatment effects and potential effect modifiers that vary across the included studies. Model (1) for the random‐effects model can be written as a regression model:

$${\hat{\theta }}_{i}=b_{0}+\delta_{i}+\varepsilon_{i},\;\text{Model}\;\left(4\right)$$

where ${\hat{\theta }}_{i}$ is study *i*'s reported treatment effect, such as the difference in mean PPD reduction, $b_{0}$ is the intercept, $\delta_{i}$ is study *i*'s draw of the random effect for between‐study heterogeneity, with zero mean and variance $\tau^{2}$, and $\varepsilon_{i}$ is sampling error, with zero mean and SD equal to the SE of ${\hat{\theta }}_{i}$. As Model (4) has no explanatory variables, the intercept $b_{0}$ is the weighted mean of the ${\hat{\theta }}_{i}$, equivalent to $\hat{\theta }$ in Model (1). We can then add potential effect modifiers to Model (4), hoping to explain between‐study heterogeneity $\delta_{i}$. For example, four of the 10 studies in Figure 1 used resorbable membranes in GTR (Group 1), one used both resorbable and non‐resorbable membranes (Group 2), and five used non‐resorbable membranes (Group 3). Therefore, the meta‐regression model for the types of membranes used in GTR can be written as:

$${\hat{\theta }}_{i}=b_{0}+b_{1}\text{Group}1_{i}+b_{2}\text{Group}2_{i}+\delta_{i}+\varepsilon_{i},$$

where ${\hat{\theta }}_{i}$ is the difference in PPD reduction between GTR and EMD, $\text{Group}1_{i}$ is a dummy variable taking the value 1 if study *i* used resorbable membranes only and 0 otherwise, while $\text{Group}2_{i}$ is a dummy variable taking the value 1 if study *i* used both resorbable and non‐resorbable membranes and 0 otherwise. The intercept $b_{0}$ is estimated to be 1.06 mm (*p* < 0.05) and represents the mean difference for the reference group of studies with non‐resorbable membranes. The regression coefficient $b_{1}$ for resorbable vs. non‐resorbable membranes is −1.16 mm (95% CI: −2.13 to −0.20), indicating a significantly smaller PPD reduction for resorbable membranes compared with non‐resorbable membranes. Resorbable membranes show no better effect than EMD on PPD reduction $\left(1.06-1.16=-0.10\right)$. The regression coefficient $b_{2}$ for *Group*2 is −0.96 mm (95% CI: −2.51 to 0.59), suggesting that GTR and EMD in the single study with both resorbable and non‐resorbable membranes achieved similar results (1.06−0.96=0.10). Between‐study variability (${\hat{\tau }}^{2}$) is reduced from 0.55 to 0.34, and $I^{2}$ from 76.6% to 66.4%.

Because membrane type is a categorical variable, we can also do a subgroup meta‐analysis in which the included studies are divided into three groups. In Figure 4, the average difference in PPD reduction between GTR and EMD is estimated for each subgroup; an overall estimate is shown at the bottom of the forest plot. Heterogeneity is estimated within each subgroup and also between subgroups. Results from the subgroup analysis are like those from the meta‐regression, with little difference between GTR with resorbable membranes and EMD, while GTR with non‐resorbable membranes achieves 1 mm greater PPD reduction than EMD. Heterogeneity remains high ($I^{2}=79.1\%$), however, for GTR with non‐resorbable membranes. This suggests that non‐resorbable membranes may, on average, achieve better but less predictable results. In contrast, resorbable membranes achieve worse but more predictable results ($I^{2}=0\%$). Therefore, whether GTR with non‐resorbable membranes is the preferred treatment depends on whether predictable results can be achieved.

**FIGURE 4:**
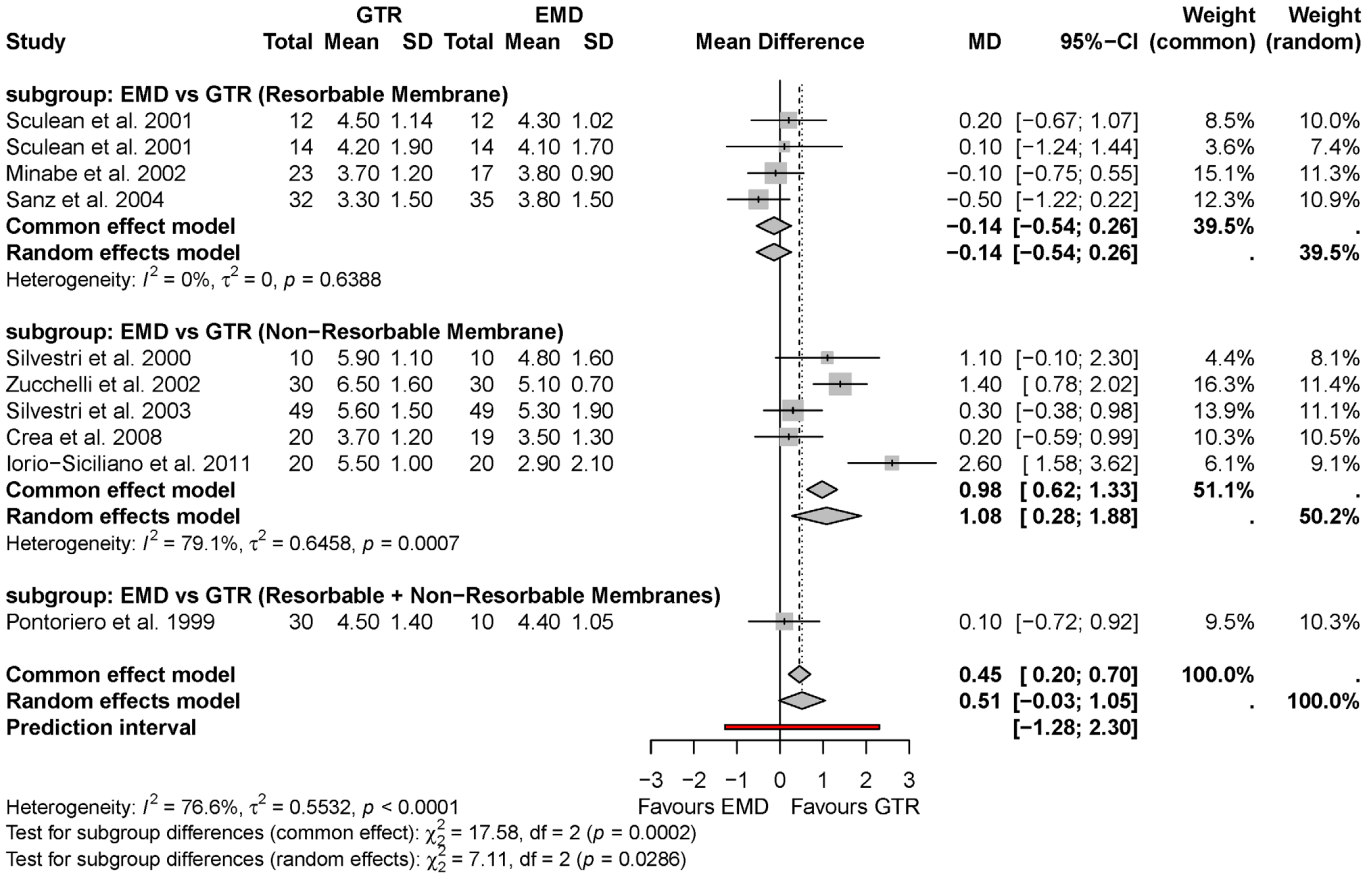
Forest plot for subgroup meta‐analysis of the difference in PPD reduction between GTR and EMD.

## 3 Network meta‐Analysis for Comparisons of Multiple Interventions

In most clinical scenarios, it is necessary to consider the pros and cons of several treatment strategies. However, doing multiple pairwise meta‐analyses to evaluate more than two treatments has serious limitations. Because the number of available studies differs between the pairwise comparisons, some pairwise meta‐analyses may lack statistical power to detect a difference between treatments in efficacy or complications. This can lead to inconclusive or even inconsistent results. For instance, suppose we want to determine whether two new and more expensive treatments, B and C, are better than the standard treatment A. We do three pairwise meta‐analyses (A vs. B, B vs. C, A vs. C) to synthesise evidence about each pair of treatments. The first meta‐analysis shows B is significantly better than A. The second meta‐analysis shows C is better than A but the difference is not statistically significant. These results seem to suggest that B is more effective than C. However, the third meta‐analysis, of a different set of clinical trials, shows that B is slightly less effective than C. We are now in a conundrum: is C better than B or not? These pairwise meta‐analyses use different sets of clinical trials for each comparison, so the evidence base varies across comparisons.

Network meta‐analysis (NMA), which emerged about 20 years ago, aims to address these limitations by incorporating all relevant evidence into a single statistical analysis comparing all available treatments [34, 35, 36]. It combines direct and indirect evidence to evaluate the performance of multiple treatments. The number of systematic reviews using NMA has increased dramatically in recent years since more user‐friendly software packages have become available. However, NMA's validity relies on several assumptions, and a proper interpretation of NMA results requires careful evaluation of these assumptions in the light of subject‐domain knowledge.

### 3.1 Evidence Geometry of Network meta‐Analysis

A network plot is commonly used to show the distribution or geometry of evidence from studies included in a network meta‐analysis. Figure 5 shows a network plot for an across NMA comparing five treatments. Each node in the plot represents a treatment modality; a node's area is proportional to the number of patients receiving the treatment. An edge connecting two nodes indicates that at least one study directly compared the two treatments. The width of an edge is proportional to the number of studies shown on the edge, comparing the two treatments. For treatments that form a loop, such as treatments A, B and C in Figure 5, we can evaluate consistency of estimates given by direct and indirect evidence. Evidence geometry also determines how biased evidence in part of a network affects the relative treatment effects between two nodes in the network [37].

**FIGURE 5:**
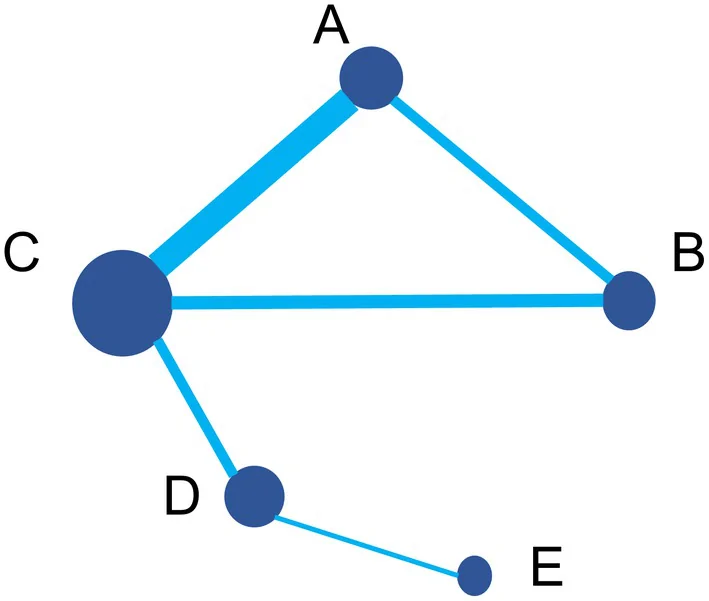
A network plot for a network meta‐analysis comparing five treatments A to E. Each node in the plot represents a treatment modality; the area of each node is proportional to the number of patients receiving the treatment. An edge connecting two nodes indicates that at least one study directly compared the two interventions. The width of each edge is proportional to the number of studies shown on each edge, comparing the two interventions.

### 3.2 Statistical Models for Network meta‐Analysis

#### 3.2.1 Contrast‐Based Model

The contrast‐based model, also known as the Lu‐Ades model [35, 38, 39], is the most popular statistical approach to NMA. It was first implemented in a Bayesian statistical framework; a frequentist version is available in the statistical software packages R and Stata [40, 41]. Suppose we have a set of trials comparing K treatments, with each trial comparing at least two treatments. We first calculate a treatment contrast between each pair of treatments in each trial. Trials with multiple treatments contribute more than one treatment contrast to the analysis. These treatment contrasts are then used to estimate the relative effects of each treatment relative to a reference treatment (e.g., A), as follows. In study *i*, the average response of patients receiving treatment *k* (k=AtoK) is ${\hat{\theta }}_{\mathrm{ik}}$, which is assumed to estimate the true mean response $\theta_{\mathrm{ik}}$ with error arising from sampling variation, so ${\hat{\theta }}_{\mathrm{ik}}=\theta_{\mathrm{ik}}+\epsilon_{\mathrm{ik}}$ where $\epsilon_{\mathrm{ik}}$ is assumed to follow a normal distribution with mean 0 and SD equal to the (known) SE of ${\hat{\theta }}_{\mathrm{ik}}$. Then $\theta_{\mathrm{ik}}$ is further modelled as:

$$\begin{array}{c} \theta_{\mathrm{ik}}=\left\{\begin{array}{cc} \mu_{\mathrm{ib}}\; & \text{if}\;k=b\;\\ \mu_{\mathrm{ib}}+\delta_{\mathrm{ibk}} & \text{if}\;k\;\text{is not}\;b \end{array}\right.\;\;\text{Model}\;\left(5\right) \end{array}$$

$$\delta_{\mathrm{ibk}}\sim N\left(d_{\mathrm{bk}},\tau^{2}\right)=N\left(d_{\mathrm{AK}}-d_{\mathrm{Ab}},\tau^{2}\right)$$

where b is a baseline or reference treatment that the meta‐analyst chooses for study *i*, $\mu_{\mathrm{ib}}$ is the true mean response of patients in study i receiving the baseline treatment b, and $\delta_{\mathrm{ibk}}$ is the difference in true treatment response between patients receiving b and k and is assumed to follow a normal distribution with mean $d_{\mathrm{bk}}=d_{\mathrm{AK}}-d_{\mathrm{Ab}}$ and variance $\tau^{2}$.

For instance, suppose an NMA compares flap operation (FO), GTR and EMD to treat intrabony defects, and we use letters A, B and C respectively to denote these three treatments in Model (5). The analysis includes clinical trials that compared two or more of the three treatments. Trial 1 compared GTR to FO, and FO is chosen as the baseline treatment for this trial. The average PPD reduction observed in patients receiving FO is ${\hat{\theta }}_{1A}$, which is used to estimate $\mu_{1A}$. ${\hat{\theta }}_{1B}$, the average PPD reduction observed in patients receiving GTR, is used to estimate $\mu_{1A}$ and $\delta_{1\mathrm{AB}}$. Because ${\hat{\theta }}_{1A}$ provides the only information for $\mu_{1A}$, ${\hat{\mu }}_{1A}={\hat{\theta }}_{1A}$, and ${\hat{\theta }}_{1B}-{\hat{\theta }}_{1A}$ is used to estimate $\delta_{\mathrm{iAB}}$. This is how the contrast‐based model got its name, because ${\hat{\theta }}_{1B}-{\hat{\theta }}_{1A}$ is a treatment contrast within study 1. Any trial comparing A with B provides data for estimating $\delta_{\mathrm{iAB}}$. For trials comparing A with C, their treatment contrasts ${\hat{\theta }}_{iC}-{\hat{\theta }}_{iA}$ are used to estimate $\delta_{\mathrm{iAC}}$. For trials comparing B with C, we use B as the baseline treatment and ${\hat{\theta }}_{iC}-{\hat{\theta }}_{iB}$ for estimating $\delta_{\mathrm{iBC}}$. It would make no difference if we chose C as the baseline treatment. However, the mean of $\delta_{\mathrm{iBC}}$, $d_{\mathrm{BC}}$, is expressed as $d_{\mathrm{AC}}-d_{\mathrm{AB}}$, so for a network that compares *k* treatments, k−1 relative effects are estimated, known as basic parameters [35], to ensure that all pairwise relative effects are consistent, that is, for any treatment contrast, its effect $d_{\mathrm{jk}}=d_{\mathrm{Ak}}-d_{\mathrm{Aj}}$. A is, therefore, the global baseline or reference treatment for this NMA, and we can choose any treatment as A for a convenient interpretation of results.

An important feature of the contrast‐based model is that each study must provide an estimate of at least one treatment contrast; therefore, studies with only a single arm are excluded. Furthermore, the contrast‐based model estimates only relative treatment effects, not absolute effects (average outcome) of each treatment. For instance, an NMA about periodontal regeneration gives estimates of differences in PPD reduction between each pair of surgical treatments; it does not provide estimates of PPD reduction for each treatment. This is the same as the pairwise meta‐analysis shown in Figure 1, which shows only the average difference between GTR and EMD in PPD reduction.

In Model (5), the heterogeneity variance $\tau^{2}$ represents variation across trials in a given treatment contrast, so Model (5) can be seen as a random‐effects model. When $\tau^{2}=0$, Model (5) reduces to a common‐effect model. An estimated treatment contrast is a weighted combination of estimates given by direct evidence—comparisons within a single trial—and indirect evidence. Indirect evidence for the contrast C vs. A, for instance, arises from combining the comparison of C vs. B and B vs. A. Indirect evidence is generally less informative than direct because its variance is the sum of the variances of two treatment contrasts instead of one.

#### 3.2.2 Bayesian Approach

The contrast‐based model was first proposed within the Bayesian statistical framework, although the frequentist version usually yields similar results. In the Bayesian framework, prior beliefs about the model parameters are specified and incorporated into the analysis. Posterior distributions of the model parameters are then computed from the prior information and the observed data. For example, a prior for the mean difference in PPD reduction may be assumed to follow a normal distribution with a mean of 1 mm and a SD of 2 mm. This suggests that the mean difference is most likely to be 1, and the range is from −3 to 5 mm. However, a low‐information prior is usually used to minimise the impact of prior information on the final results, since evidence supporting a particular hypothesis is often unavailable [35, 42]. For example, we may specify a prior for the mean difference in PPD reduction with a normal distribution of a zero mean and a SD of 50 mm. Since the PPD reduction in surgical treatments for periodontal regeneration typically differs by only a few millimetres, the prior probability across this possible range is almost the same. The posterior distribution of the model parameters is therefore mainly determined by the observed data.

Because most statistical models, such as Model (5), include many unknown parameters (e.g., $\mu_{\mathrm{ik}}$ and $\delta_{\mathrm{ibk}}$), software packages use algorithms based on the Markov Chain Monte Carlo (MCMC) method to compute approximate posterior probability distributions. MCMC begins with a set of starting values and iteratively samples from the joint posterior distribution of the model parameters. Samples taken from this distribution are then used to estimate the posterior distribution of each parameter in the model [42, 43]. Summary statistics, such as mean or median, are used to present the central location of the distribution, and a 95% credible interval represents the range within which the probability of an unknown model parameter falls, based on observed data and prior beliefs.

## 4 Assumptions Behind Network meta‐Analysis

### 4.1 Homogeneity

NMA requires several assumptions to obtain meaningful estimates of treatment effects. The first assumption is homogeneity. A pairwise meta‐analysis compares one pair of treatments; an NMA compares multiple pairs. The homogeneity assumption in pairwise meta‐analysis requires that the included trials comparing a given pair of treatments are comparable with respect to key factors that moderate patients' response to treatment, such as patient characteristics, study setting, length of follow‐up and outcome measures [44]. If we extend the homogeneity assumption to trials in an NMA that compare different pairs of treatments, this leads to the consistency and transitivity assumptions, which we now describe.

### 4.2 Consistency Between Direct and Indirect Evidence

NMA assumes consistency between direct and indirect evidence and calculates a weighted average of them to provide the best estimate of the difference in effect between two treatments [45]. In practice, small differences between direct and indirect evidence may be observed, and they are usually interpreted as random sampling errors. When the differences are large and cannot be attributed to random variation, we need to investigate potential sources of inconsistency, such as heterogeneity in study protocols (e.g., drug doses, follow‐up duration, patient inclusion criteria), patient populations (e.g., age, comorbidities) and methods of assessing outcomes.

When none of the included trials compares more than two treatments, it is relatively straightforward to separate direct from indirect evidence. Evaluating inconsistency becomes more complex when some trials compare three or more treatments. Three statistical models are commonly used to assess the consistency assumption, each with its specific focus.

#### 4.2.1 Design‐By‐Treatment Interaction Model

The distinction between direct and indirect evidence becomes less clear in the presence of multi‐arm trials. For instance, a trial that compares three treatments A, B and C provides direct evidence for the difference between three pairs of treatments, $\theta_{\mathrm{AB}}$, $\theta_{\mathrm{BC}}$ and $\theta_{\mathrm{AC}}$. However, these three estimates are necessarily consistent because any one treatment contrast can be derived from the other two. Focusing on this internal coherence, the design‐by‐treatment interaction model distinguishes between estimates of a treatment contrast obtained from different ‘designs’ of trials [46, 47]. In this context, a *design* refers to a set of trials that compare a given set of treatments. In using the design‐by‐treatment interaction model, trials are first grouped into different ‘designs’, and differences in treatment estimates between different designs are evaluated using the design‐by‐treatment interaction. Figure 6 shows an NMA with four designs comparing three treatments, FO, GTR and EMD: design AB compares FO with GTR, design AC compares FO with EMD, design BC compares GTR with EMD, and design ABC compares all three treatments. For the difference between FO and GTR, designs ABC and AB provide direct comparisons, $\theta_{\mathrm{AB}}^{\left(\mathrm{ABC}\right)}$ and $\theta_{\mathrm{AB}}^{\left(\mathrm{AB}\right)}$, respectively, while designs BC and AC provide indirect comparisons, $\theta_{\mathrm{BC}}^{\left(\mathrm{BC}\right)}$ and $\theta_{\mathrm{AC}}^{\left(\mathrm{AC}\right)}$, respectively. The total variation in the reported differences is partitioned into between‐design variation (i.e., inconsistency or the design‐treatment interaction) and within‐design variation (i.e., heterogeneity). For instance, in Figure 6, both $\theta_{\mathrm{AB}}^{\left(\mathrm{ABC}\right)}$ and $\theta_{\mathrm{AB}}^{\left(\mathrm{AB}\right)}$ are direct estimates of the mean difference in PPD reduction between FO and GTR, and the difference between $\theta_{\mathrm{AB}}^{\left(\mathrm{ABC}\right)}$ and $\theta_{\mathrm{AB}}^{\left(\mathrm{AB}\right)}$ is between‐design variation, that is, the design‐by‐treatment interaction. In contrast, differences in the reported estimates amongst the five studies with design ABC are within‐design heterogeneity.

**FIGURE 6:**
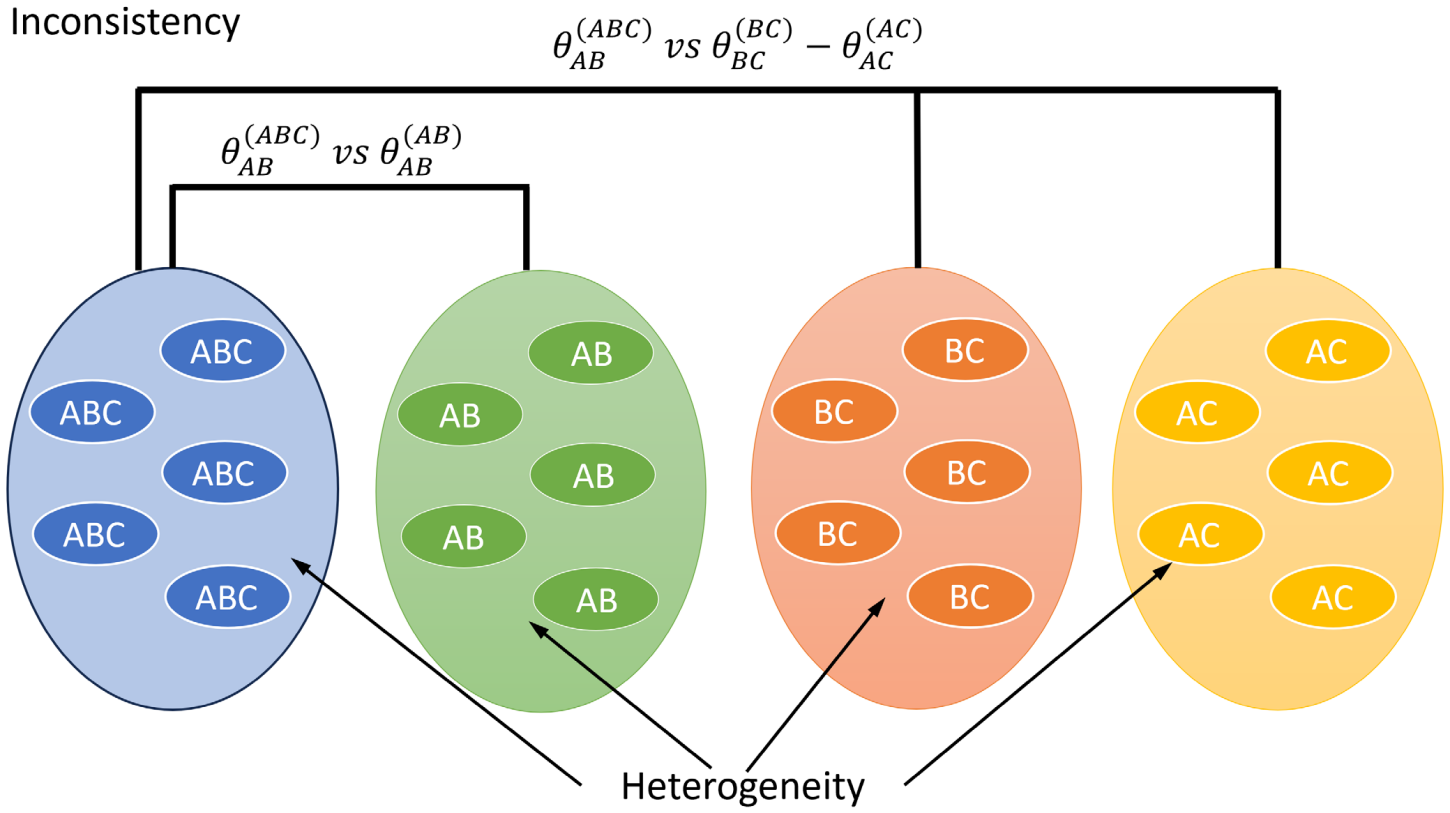
Illustration of the design‐by‐treatment interactions. Large circles of different colours represent four different study designs, each comparing 2 or 3 treatments. Each large circle contains 5 small circles, representing single studies. The blue circle, representing design ABC, and the green circle, representing design AB, provide estimates $\theta_{\mathrm{AB}}^{\left(\mathrm{ABC}\right)}$ and $\theta_{\mathrm{AB}}^{\left(\mathrm{AB}\right)}$, respectively, for the difference in treatment effects between A and B. The difference between $\theta_{\mathrm{AB}}^{\left(\mathrm{ABC}\right)}$ and $\theta_{\mathrm{AB}}^{\left(\mathrm{AB}\right)}$ is between‐design variation, that is, the design‐by‐treatment interaction, while differences in the reported estimates amongst the five studies in design ABC or in design AB are within‐design heterogeneity.

The design‐by‐treatment interaction model is commonly used with an ANOVA‐type test to evaluate global inconsistency by examining differences in treatment effect estimates between various designs within the network [48]. If the results are statistically significant, loop inconsistency and node‐splitting models are then used to identify local inconsistency.

#### 4.2.2 Loop Inconsistency Model

The loop inconsistency model assesses the potential inconsistency between direct and indirect evidence for each pair of treatments within a loop formed by three or more treatments in a network, such as that in figure 5 [45, 49]. If a loop involves only two‐arm trials, selecting any pair of treatments for assessment yields identical results. The loop inconsistency model can be viewed as a simplified version of the design‐by‐treatment model if no distinction is made between two‐arm and multi‐arm trials [46]. For example, we can merge designs AB and ABC into a single design; then the between‐design inconsistency in the direct estimates of the treatment contrast A versus B is reduced to within‐design heterogeneity. When a loop involves trials with more than two arms, we may merge designs in multiple ways, leading to different assessments of loop inconsistency. For instance, we may merge design BC with design ABC into a group. In practice, various ways of merging designs typically yield similar results, as multi‐arm trials are usually few. Readers interested in learning more can find more explanations in our previous publication [48].

#### 4.2.3 Node or Side‐Splitting Model

This model assesses the inconsistency of direct and indirect evidence by dividing trials into two groups: those that provide direct evidence and those that do not [41, 43, 45, 50]. This is equivalent to combining different designs of trials into two groups [51, 52]. Thus, both the node‐splitting model and the loop inconsistency model can be viewed as simplifications of the design‐by‐treatment interaction model. We then evaluate the difference, for each treatment contrast, between the direct and indirect evidence, except for treatment contrasts with only direct or only indirect evidence. However, as in the loop‐inconsistency model, separating direct from indirect evidence is not straightforward when an NMA includes trials with more than two arms. For example, to assess inconsistency between direct and indirect evidence in the comparison between A and B in Figure 6, we may combine designs AB and ABC into a single group of direct evidence and designs BC and AC into a separate group of indirect evidence. However, design ABC also provides indirect evidence for comparisons between B and C and between A and C, and the statistical literature has been debating how to partition the total evidence into direct and indirect components [51, 52, 53]. A simple and accurate method is to estimate indirect evidence by calculating the difference between the total evidence from the NMA and the direct evidence. Then, we compare the difference between direct and indirect estimates [52].

### 4.3 Transitivity

According to the Cochrane Handbook for Systematic Reviews of Interventions, a set of randomised trials satisfies transitivity if these trials, on average, are similar with respect to all effect modifiers other than the intervention under comparison [54, 55]. Transitivity requires that intervention A be similar across trials of A versus B and across trials of A versus C with respect to characteristics (effect modifiers) that may affect the two relative effects. The transitivity assumption is usually assessed by visualising the distribution of effect modifiers across different designs that compare different pairs of treatments. This approach works only when the number of trials for each design is large.

A recent article argued that all three assumptions, namely homogeneity, consistency and transitivity, amount to a single assumption of exchangeability [56]. In the previous section, we noted that standard meta‐analysis assumes that a given treatment contrast is exchangeable across trials. In NMA, exchangeability requires that trials of the same design are comparable, so their estimates of the same treatment contrast can be combined into a weighted mean. For trials with different designs, exchangeability requires that a treatment contrast in one design is comparable to that in other designs. For instance, treatment contrast A vs. B in design AB is comparable to that contrast in design ABC. Had trials with design BC also compared A, or had trials with design AC compared B, they would have produced estimates for the treatment contrast AB similar to those from trials in design AB. If this exchangeability assumption holds, direct and indirect evidence will, therefore, be consistent [57, 58]. Box 4 summarises the key statistical concepts for assessing the assumptions of NMA.

Violations of the transitivity or exchangeability assumptions could have major ramifications for interpreting NMA results. For instance, suppose A is a minimally invasive surgical technique for root coverage, while B is a standard surgical technique with a connective tissue graft. Both are compared to the coronally repositioned flap (CRF) technique. Clinical trials show that A achieves a 90% success rate for complete root coverage, while CRF achieves 60%. Other trials show that B achieves a 50% success rate for complete root coverage, while CRF achieves 30%. An indirect comparison of A versus B is meaningful only when the two groups of trials use similar criteria for selecting patients and defects. For instance, if trials on A versus CRF include mainly teeth with gingival recession of Class I & II in Miller's classification, while trials on B versus CRF include many teeth with Class III & IV, the indirect comparison violates the transitivity/exchangeability assumption because teeth with Class I and II gingival recession are more likely to achieve complete root coverage than those with Class III and IV.

BOX 4 **Summary of the key statistical concepts for assessing the assumptions of NMA.**

**Table:** 

Assumption	Explanation
Direct and indirect evidence	In a network meta‐analysis, trials comparing the same pair of interventions, for example, A and B, provide direct evidence on the difference in treatment efficacy between A and B. Other trials that do not include both A and B may provide indirect evidence.
Homogeneity	The homogeneity assumption in pairwise meta‐analysis requires that trials comparing a given treatment pair, such as A versus B, be comparable with respect to key effect modifiers. This assumption is extended to other treatment pairs in an NMA that includes trials comparing different treatment pairs.
Consistency	When direct and indirect evidence yield the same estimate of a relative treatment effect, the evidence is consistent. A network meta‐analysis assumes consistency for all pairwise comparisons within the network.
Transitivity	Transitivity assumes that different sets of trials have similar distributions of potential effect modifiers. For example, if trials comparing interventions A and B have a distribution of effect modifiers similar to that of trials comparing A with C, a meaningful indirect comparison between B and C is possible.
Design‐by‐treatment interaction	‘Design’ refers to a set of trials comparing the same set of treatments. The variation in estimates of treatment contrasts between different designs of trials is inconsistency, while the variation across trials of the same design is heterogeneity. The design‐by‐treatment interaction is often used to assess the overall inconsistency within an NMA by using an ANOVA‐type test.
Loop inconsistency	Loop inconsistency is identical to design‐by‐treatment interaction when the included trials in an NMA compare only two treatments. When some trials compare three or more treatments, they are merged with trials comparing two treatments to form a group. For instance, trials comparing A, B and C, are merged with trials comparing A and B to form group A. However, the formation of groups is not unique, as trials comparing A, B and C, can merge with trials comparing B and C. The loop inconsistency model is often used to assess inconsistency within a loop in a network plot.
Node‐splitting	For each treatment comparison with both direct and indirect evidence, a separate node‐splitting model is fitted. The model estimates the treatment effect using studies that directly compare the two treatments and separately estimates the same effect using the rest of the network. Inconsistency is assessed by comparing these two estimates.

### 4.4 Evaluation of Small‐Study or Publication bias

Assessing small‐study or publication bias in an NMA is more complex than in a pairwise meta‐analysis. For instance, in an NMA comparing treatments A, B and C, we may have direct evidence for the three pairwise comparisons A versus B, A versus C and B versus C. We can then draw three funnel plots to evaluate small‐study bias for each direct comparison. To evaluate small‐study bias across the comparisons, we combine the three funnel plots into a single plot. Because each pair has its own weighted mean difference, we first align the central locations of the three funnel plots to zero by subtracting the weighted mean difference for each direct comparison from each study's reported mean difference. We then evaluate the asymmetry in the distribution of studies in the combined funnel plot. However, before we combine the funnel plots, we need to decide how to calculate the mean differences in each pairwise comparison. For example, suppose treatment C came on the market later and is more expensive than B, and B appeared later and is more expensive than A. We therefore hypothesise that a small‐study or publication bias may favour the more expensive treatment. When drawing the funnel plot for NMA, we could consistently calculate the mean difference in treatment effect of each study as $\theta_{k,k^{\prime }}=\theta_{k^{\prime }}-\theta_{k}$, where $\theta_{k^{\prime }}$ is the effect of the more expensive treatment, and $\theta_{k}$ is the effect of the less expensive one. If small studies are more likely to report a greater benefit of a more expensive treatment over a less expensive one, we will observe more studies in the bottom‐right corner of the funnel plot than in the bottom left.

In this simple scenario, it may be easy to determine the order of treatments in calculating $\theta_{k,k^{\prime }}$. However, when we compare many treatments in an NMA, it may not be straightforward to determine the order of treatments. Should we order treatments by cost, or by the date when they became available? What if two treatments have similar costs or become available at a similar time? There are many factors to consider and people may have different opinions. Different orderings of treatments will produce different funnel plots, giving rise to different conclusions. Thus, researchers should provide rationales for their treatment ordering, and the funnel plot and results from the associated statistical tests should be interpreted with great caution [59].

### 4.5 Treatment Ranking

Because the Bayesian approach is simulation‐based, it is easy to rank treatments by calculating the probability that each treatment occupies each position. For example, suppose we make 10 000 MCMC draws for a network meta‐analysis comparing treatments A, B and C. Treatment A ranks first in 5000 simulations, second in 4000, and third in 1000. Thus, the ranking probabilities of being best, second, and third for A are estimated at 0.5, 0.4 and 0.1, respectively. We can perform the same calculations for the other two treatments. An overall performance index, called the surface under the cumulative ranking curve (SUCRA), ranging from 0 to 1, can then be derived from the ranking probabilities of each treatment [60, 61, 62]. The higher the SUCRA value, the greater the likelihood that a treatment is better than the others in the network; the closer the SUCRA value is to 0, the greater the likelihood that a treatment is worse than the others. SUCRA is particularly useful when differences in treatment effects are small. Estimating ranking probabilities is not restricted to the Bayesian approach. A simulation approach within a frequentist statistical framework has been implemented in the ‘network’ suite for Stata software [41]. The P‐score, derived from the *p*‐values for the pairwise comparisons between treatments in an NMA, has been shown to be identical to SUCRA under certain assumptions [63]. It is available in the ‘netmeta’ package for R software [40].

## 5 Example of Network Meta‐Analysis in Periodontal Regeneration

### 5.1 Example Data

This section uses an example comparing the effectiveness of surgical interventions for periodontal regeneration of intrabony defects. Details of the systematic review, including the literature search, study selection, data extraction and quality assessment of individual studies, are in the original article [18]. Because an NMA compares multiple treatments, a systematic review is more time‐consuming and error‐prone. Although it is beyond this article's scope to discuss the whole procedure in detail, we cannot overstate the importance of conducting a rigorous systematic review before analysing the data.

Although an NMA is more complex than a pairwise meta‐analysis, it follows similar principles. For example, the research question is first formulated using the PICOS framework, while interventions in an NMA consist of a group of treatments rather than a single one. The comparator is usually a global reference treatment, such as a placebo or standard care. Note that for a study to be included in an NMA, it does not need to include the comparator; it only needs to include at least two of the intervention or comparator treatments. NMA follows the same principles for selecting the effect size measure for treatment outcomes and conducting data extraction and reconstruction. Since NMA involves more studies comparing multiple treatments, clinical and statistical heterogeneity is expected, so the random‐effects model is usually the preferred approach to data analysis.

The original NMA of this dataset was published in 2020 [18]; we repeated the same literature search strategy to update the analysis with data from eligible trials published between 1 October 2019 and 15 December 2025. Seven additional randomised controlled trials comparing two surgical interventions were identified [64, 65, 66, 67, 68, 69, 70]. For PPD reduction, a total of 65 trials were included in the updated analysis: 56 two‐arm, 8 three‐arm and 1 four‐arm. Forty‐four trials used a parallel‐group design, while 21 used a split‐mouth design. Table S2 shows the extracted data used in our analysis. A total of 3095 patients were assigned to 1 of 8 treatment modalities: flap operation (FO), EMD, EMD with bone grafts (EMD + BG), EMD with platelet‐rich plasma/fibrin and bone grafts (EMD + PRP + BG), GTR, GTR with EMD (GTR + EMD), GTR with bone grafts (GTR + BG) and GTR with platelet‐rich plasma/fibrin and bone grafts (GTR + PRP + BG). The sample sizes ranged from 7 to 83 per treatment group, with an average of 22 per group. For this tutorial, we used the ‘netmeta’ package in R to do the data analysis [40].

In the following sections, we break the process of conducting a network meta‐analysis into six steps:

Step 1. Draw a network plot.
Step 2. Conduct statistical analysis and interpret the results
Step 3. Evaluate the inconsistency between direct and indirect evidence:
Step 4. Evaluate the transitivity assumptions.
Step 5. Calculate the ranking of treatments.
Step 6. Conduct subgroup analysis and meta‐regression to explore heterogeneity.

### 5.2 Step 1: Draw a Network Plot

Figure 7 is a network plot showing the evidence geometry of the data included in the analysis. Each of the eight nodes in the plot represents a surgical treatment. The plot has 13 edges, indicating that 13 of the 28 pairwise comparisons had direct evidence, while the remaining 15 had only indirect evidence. Two comparisons (GTR + BG vs. GTR + PRP + BG and EMD + BG vs. EMD + PRP + BG) have only direct evidence. The number of studies that compare two treatments is shown on the edge corresponding to that treatment pair. The distribution of studies is uneven, with the most comparisons between GTR and FO and between EMD and FO, with 18 and 19 studies, respectively. Figure 7 has several loops; consistency between direct and indirect evidence within each loop needs to be assessed.

**FIGURE 7:**
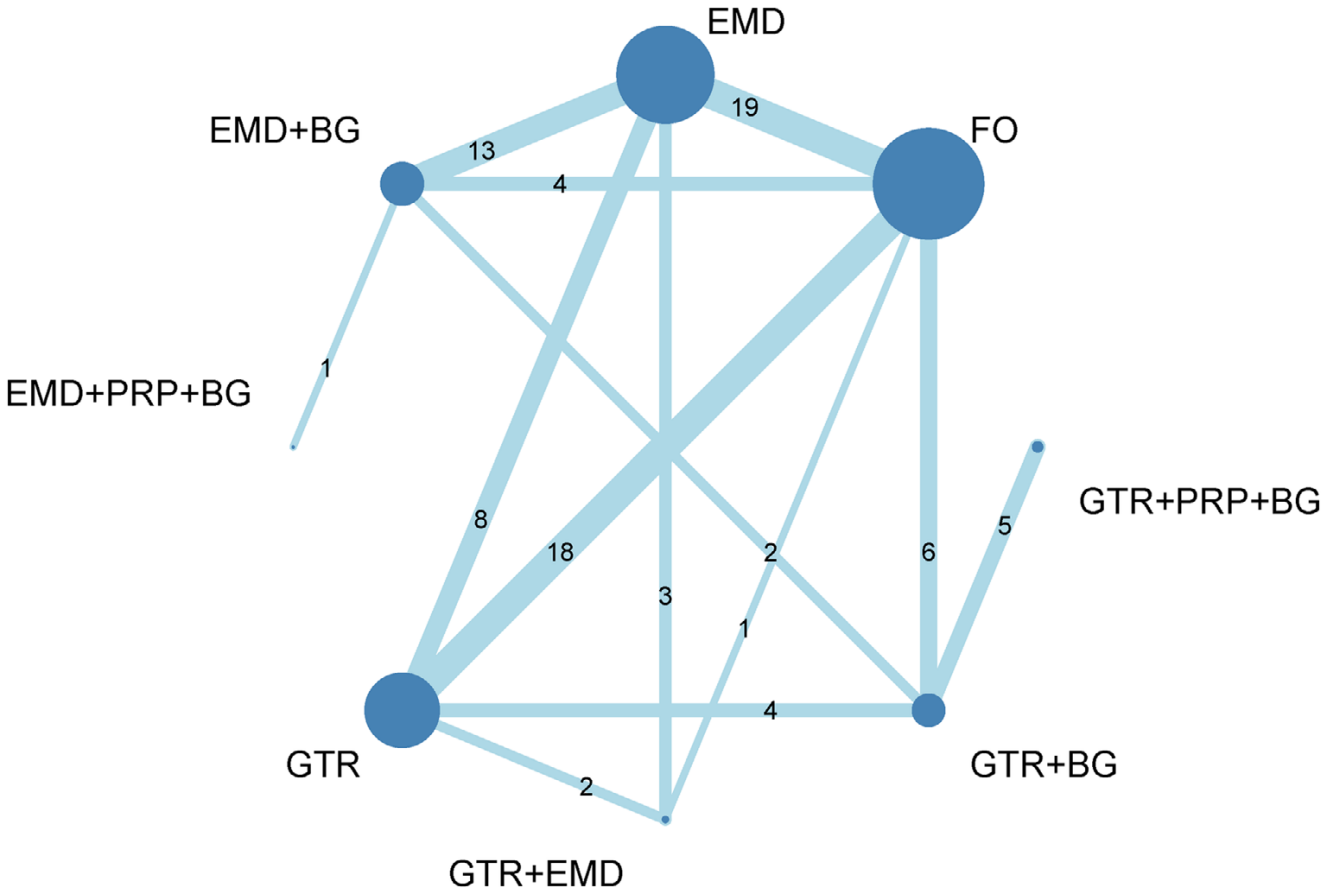
Network plot for a NMA comparing eight surgical interventions for treating intrabony defects. Each node represents an intervention; each edge represents trials comparing the interventions at its ends.

### 5.3 Step 2: Conduct Statistical Analysis and Interpret the Results

Figure 8 uses a forest plot to show the results of our network meta‐analysis, expressed as the mean difference in PPD reduction (mm) between FO and another periodontal regenerative surgery. The mean difference is shown as a tick, and the confidence interval as a horizontal line. The vertical solid line at 0 indicates no difference. If a horizontal line crosses the vertical solid line, for example, for EMD + PRP + BG, this indicates that the mean difference is not statistically significant. The differences range from 0.88 mm to 1.28 mm, and except for EMD + PRP + BG, regenerative surgeries achieve significantly greater PPD reduction than FO. Table 1, known as a league table, shows all pairwise comparisons from the NMA in its lower triangle. Most pairwise comparisons between regenerative surgeries found differences that were small and statistically nonsignificant. However, GTR achieves a significantly greater PPD reduction than EMD, by 0.38 mm (95% CI: 0.02 to 0.74). The SD of between‐study heterogeneity is estimated to be 0.63 mm, which is large compared to the estimated mean differences in PPD reduction between the eight surgical interventions. This suggests that although GTR achieved, on average, a slightly greater PPD reduction, a new study comparing GTR with other surgical interventions may find that GTR is not superior to these treatments. Furthermore, bone grafts or platelet‐rich plasma do not appear to provide additional benefit for treating intrabony defects compared with GTR or EMD alone.

**FIGURE 8:**
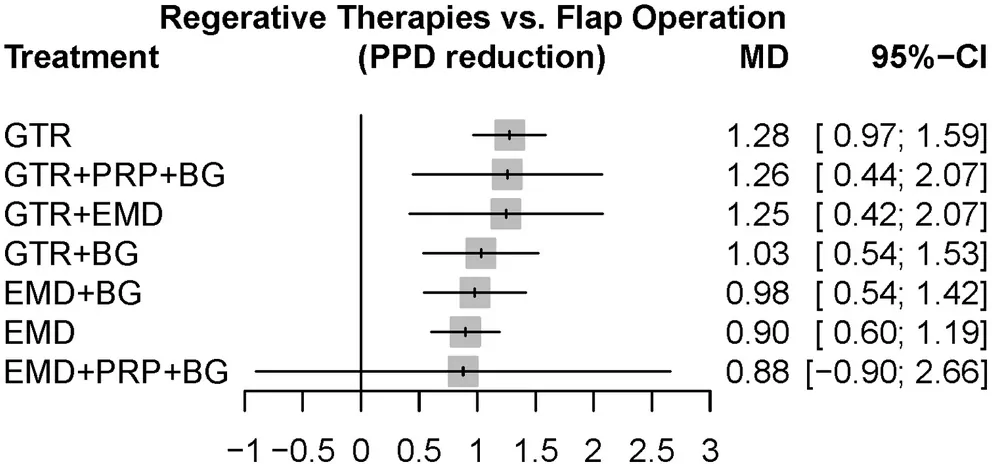
Forest plot for the differences in PPD reduction between flap operation (FO) and the other periodontal regenerative surgeries. EMD: Enamel matrix derivatives; EMD + BG: EMD with bone grafts; EMD + PRP + BG: EMD with platelet‐rich plasma/fibrin and bone grafts; GTR: Guided tissue regeneration; GTR + EMD: GTR with EMD; GTR + BG: GTR with bone grafts; and GTR + PRP + BG: GTR with platelet‐rich plasma/fibrin and bone grafts.

**TABLE 1:** League table of pairwise comparisons between the treatments. The matrix's lower triangle shows results from NMA, while the matrix's upper triangle shows direct evidence only. Each cell gives the estimated difference between the column and row treatments with 95% confidence interval.

	GTR	GTR + PRP + BG	GTR + EMD	GTR + BG	EMD + BG	EMD + PRP + BG	EMD	FO
GTR	GTR	—	−0.39 (−1.53 to 0.75)	−0.10 (−0.86 to 0.66)	—	—	0.60 (0.07 to 1.14)	1.28 (0.92 to 1.64)
GTR + PRP + BG	0.02 (−0.81 to 0.85)	GTR + PRP + BG	—	0.22 (−0.42 to 0.87)	—	—	—	—
GTR + EMD	0.03 (−0.80 to 0.87)	0.01 (−1.13 to 1.16)	GTR + EMD	—	—	—	0.30 (−0.60 to 1.20)	0.60 (−1.01 to 2.21)
GTR + BG	0.25 (−0.28 to 0.77)	0.22 (−0.42 to 0.87)	0.21 (−0.73 to 1.16)	GTR + BG	−0.20 (−1.49 to 1.09)	—	—	0.95 (0.34 to 1.56)
EMD + BG	0.30 (−0.20 to 0.79)	0.28 (−0.61 to 1.16)	0.27 (−0.62 to 1.16)	0.05 (−0.55 to 0.66)	EMD + BG	0.10 (−1.63 to 1.83)	0.15 (−0.27 to 0.56)	0.57 (−0.21 to 1.34)
EMD + PRP + BG	0.40 (−1.40 to 2.20)	0.38 (−1.56 to 2.32)	0.37 (−1.58 to 2.31)	0.15 (−1.68 to 1.99)	0.10 (−1.63 to 1.83)	EMD + PRP + BG	—	—
EMD	0.38 (0.02 to 0.74)	0.36 (−0.48 to 1.20)	0.35 (−0.46 to 1.16)	0.13 (−0.41 to 0.68)	0.08 (−0.30 to 0.46)	−0.02 (−1.79 to 1.75)	EMD	1.00 (0.66 to 1.33)
FO	1.28 (0.97 to 1.59)	1.26 (0.44 to 2.07)	1.25 (0.42 to 2.07)	1.03 (0.54 to 1.53)	0.98 (0.54 to 1.42)	0.88 (−0.90 to 2.66)	0.90 (0.60 to 1.19)	FO

### 5.4 Step 3: Evaluating Inconsistency Between Direct and Indirect Evidence

The ‘netmeta’ package provides an overall ANOVA‐type test for the global design‐by‐treatment inconsistency, which is not statistically significant (*p* = 0.36). Figure 9 shows results from the node‐splitting model; no difference between direct and indirect estimates is statistically significant. This should not, however, be interpreted as evidence of consistency. For instance, both direct and indirect evidence indicate that EMD is superior to FO, with the two differing only in the extent of EMD's superiority. In contrast, direct evidence shows GTR achieves slightly smaller PPD reduction than GTR + BG (−0.10 mm, 95% CI: −0.86 to 0.66), while indirect evidence shows GTR achieves greater PPD reduction, about 0.55 mm (95% CI: −0.17 to 1.27). Because assessments of inconsistency often lack statistical power, interpretation should be cautious when direct and indirect evidence point in opposite directions.

**FIGURE 9:**
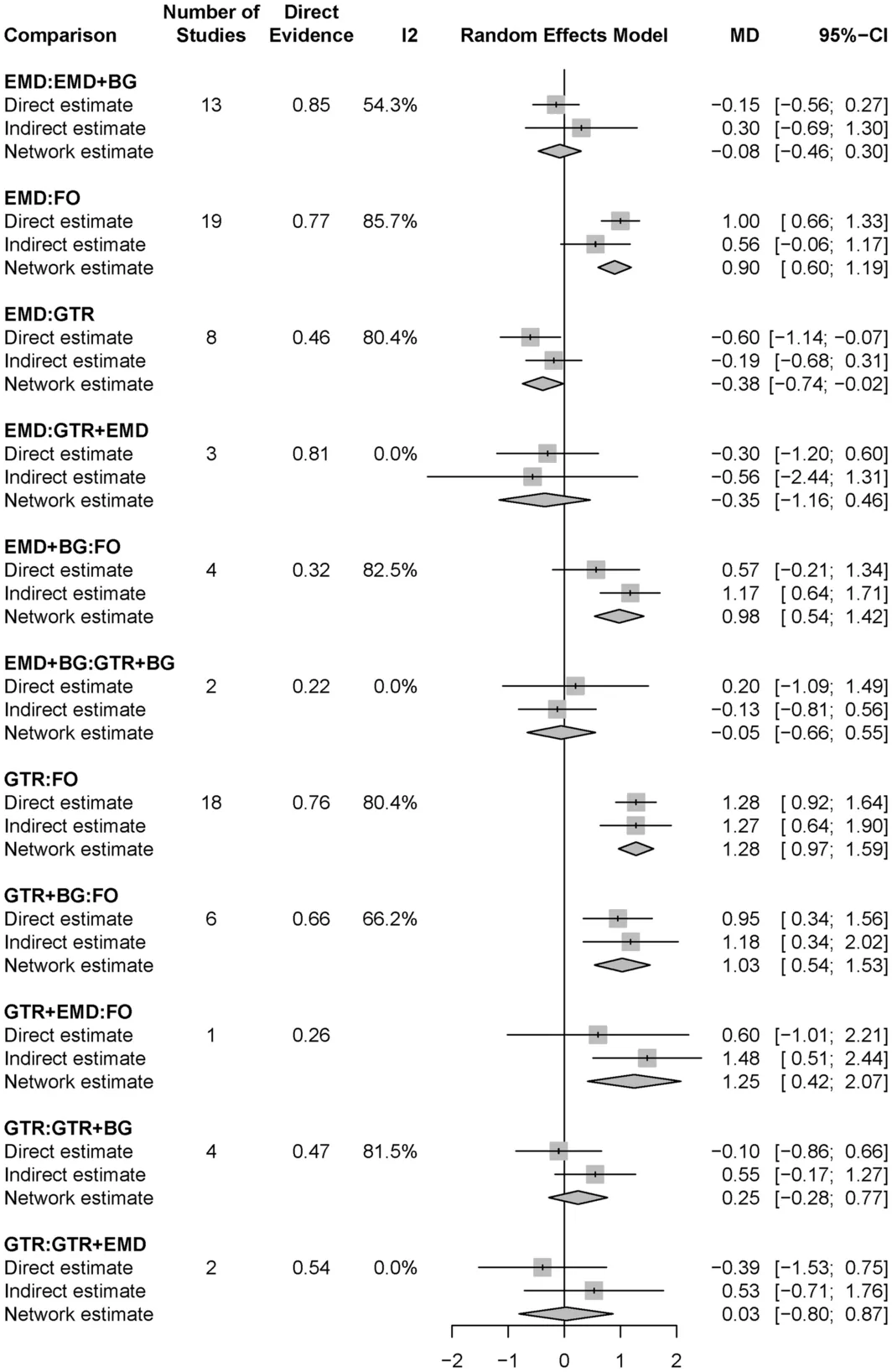
Forest plot of the node‐splitting model for assessing inconsistency. The column labelled ‘Direct Evidence’ shows the proportion of the direct evidence in the total evidence for a pairwise comparison. The column labelled ‘I2’ is the heterogeneity index $I^{2}$ for the direct evidence.

### 5.5 Step 4: Evaluate the Transitivity Assumption

The most common approach to assessing the transitivity assumption is to compare the distribution of effect modifiers across study designs. Here, the design refers to studies comparing the same set of treatments as in the design‐by‐treatment interaction model for assessing inconsistency. Figure 10 shows two violin plots: the distribution of mean baseline PPD (Figure 10A) and the distribution of mean intrabony defect depth (Figure 10B). Because many designs have only a few studies, it is hard to judge whether the distributions are similar across designs. Moreover, not all studies report mean values of the effect modifiers: for example, only about half of the included studies report mean intrabony defect depth. Even if the distribution looks different across designs, how these effect modifiers moderate treatment effects remains unknown, and it is unclear how to account for differences in distribution when interpreting the results. More details about the limitations of this approach can be found in our previous publication [57].

**FIGURE 10:**
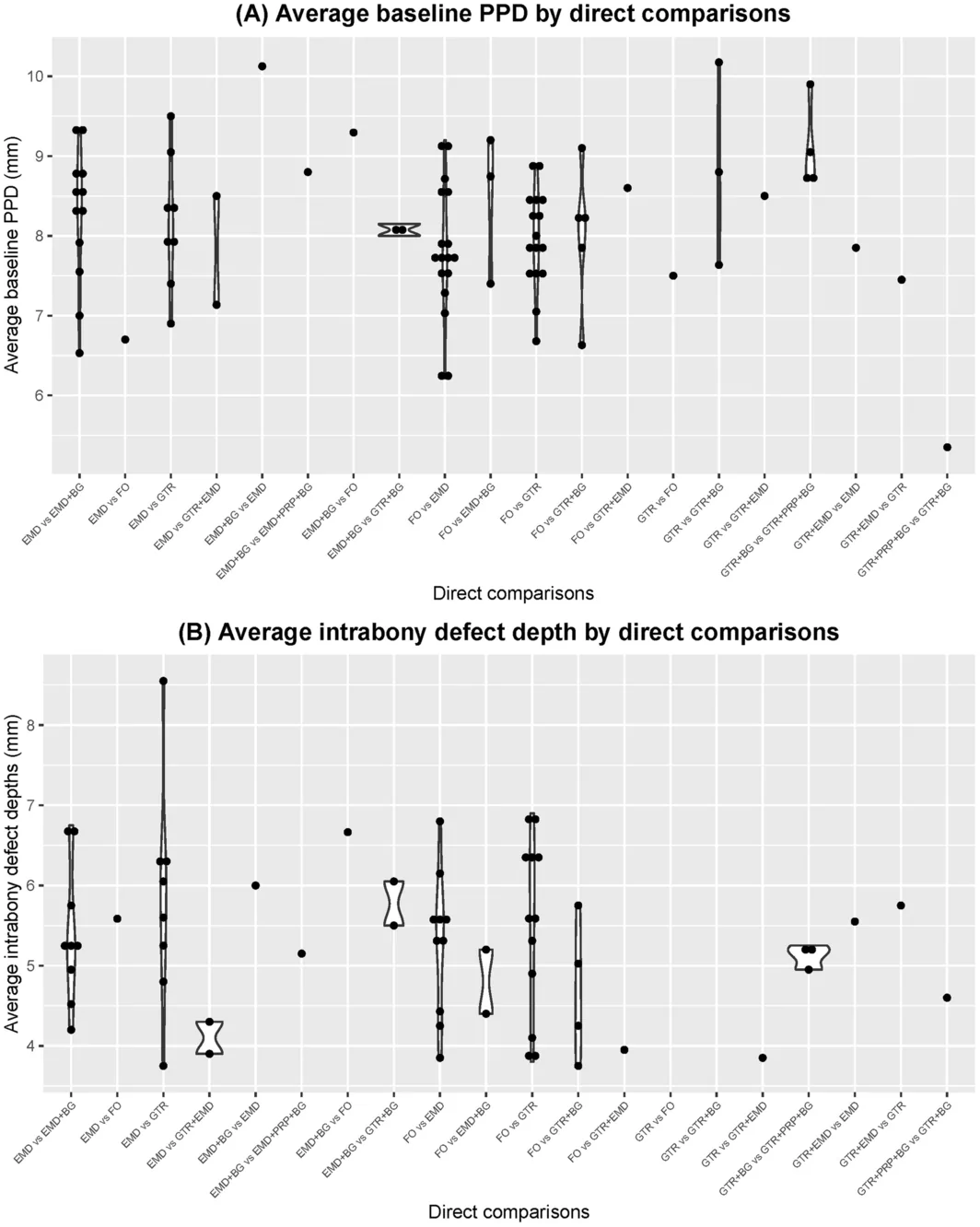
Violin plots assessing the distribution of baseline PPD and intrabony defect depth for various study designs: (A) the distribution of baseline PPD, and (B) the distribution of intrabony defect depth across different direct comparisons. Far fewer studies reported mean baseline intrabony defect depth.

We have proposed an alternative approach to assessing the transitivity assumption by visualising the relationship between absolute treatment effects and the effect modifier [57]. The horizontal axis in Figure 11 is the mean baseline PPD, and the vertical axis is the mean PPD reduction. Each dot in the scatterplot represents a treatment arm from a study, and each treatment is represented by a different colour. Both variables vary widely: mean baseline PPD ranges from 5.3 mm to 10.8 mm, and mean PPD reduction ranges from 1.4 mm to 8.25 mm. Nevertheless, for most arms the mean baseline PPD ranges from 6 mm to 10 mm, and its distribution does not differ notably between GTR and EMD. The association between mean baseline PPD and PPD reduction is positive for all treatments, indicating that mean baseline PPD is an absolute effect modifier. FO (light blue) appears to have a smaller PPD reduction than other treatments across the whole range. GTR (light purple) appears to show greater PPD reduction than EMD (light green) when the mean baseline PPD exceeds 8 mm. However, the effect of a given treatment varies substantially between different studies.

**FIGURE 11:**
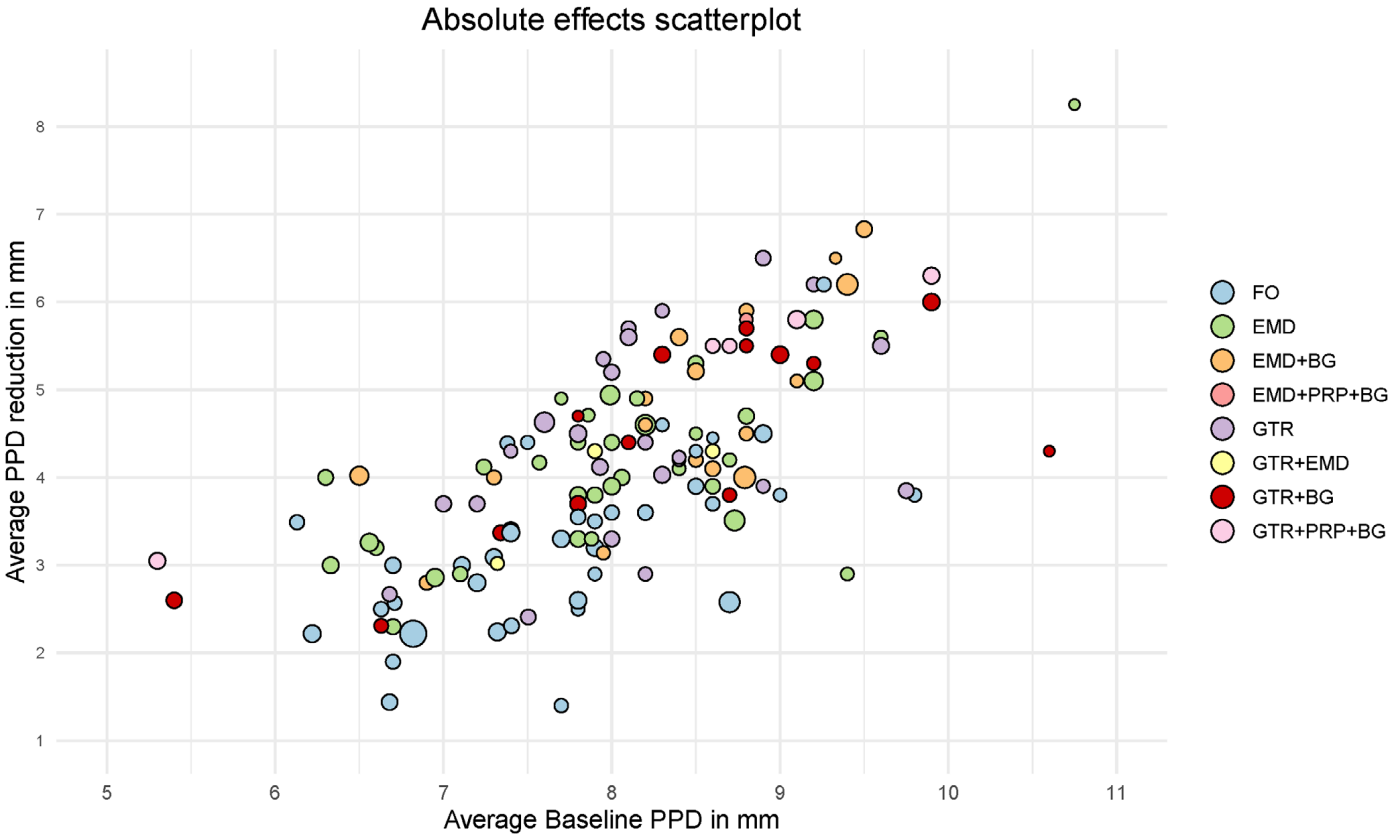
Scatterplot for the relationship between mean PPD reduction and mean baseline PPD for 63 studies that reported mean baseline PPD.

### 5.6 Step 5: Calculate the Ranking of Treatments

The P‐score shows that GTR is the most effective treatment (0.77), followed by GTR + PRP + BG (0.70), GTR + EMD (0.69), GTR + BG (0.51), EMD + BG (0.47), EMD + PRP + BG (0.46), EMD (0.37) and FO (0.02). However, differences in P‐scores between some treatments are small, and the treatment ranking is likely to change if a new study is included [61]. Figure 12 shows the cumulative ranking probability curve for each surgical intervention; the area under the curve is the P‐score or the SUCRA value in a Bayesian analysis.

**FIGURE 12:**
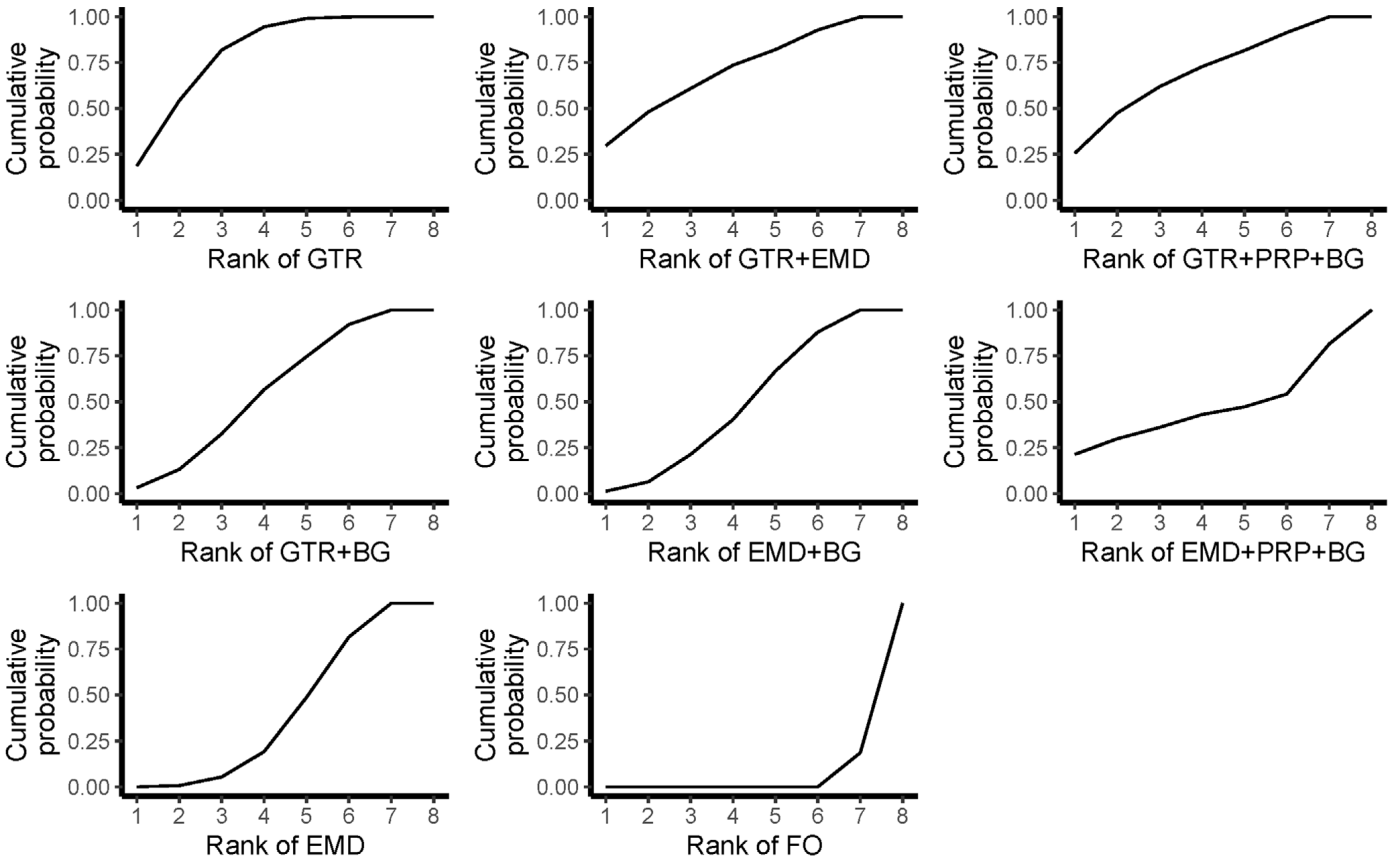
Cumulative ranking probability plots.

### 5.7 Step 6: Subgroup Analysis and Meta‐Regression

Subgroup analysis and meta‐regression can be used to explore sources of heterogeneity in NMA. However, each subgroup (e.g., by patient age) may include different treatment sets, making the interpretation of results from subgroup analyses quite challenging. In our example, 44 trials used a parallel‐group design, while 21 used a split‐mouth design. We conducted a subgroup analysis to investigate whether treatment efficacy differs between parallel‐group and split‐mouth designs (not to be confused with the ‘design’ in the evaluation of inconsistency), and the results are shown in Table 2. Because all trials that included EMD + PRP + BG used a parallel‐group design, no estimate is provided for the split‐mouth trials. Some treatment efficacies differ between trials with a parallel‐group or split‐mouth design, although no difference in the pairwise comparisons is statistically significant. Apart from the comparison between FO and GTR, trials with a split‐mouth design tend to show a larger difference. The meta‐regression using parallel‐group vs. split‐mouth design as an explanatory variable produces very similar results. The main difference is in how heterogeneity is estimated: subgroup analysis estimates heterogeneity variances separately for trials with the two different designs, while meta‐regression estimates a common heterogeneity. The estimated variance of $\tau^{2}$ in the subgroup analysis is 0.37 and 0.44 for the parallel‐group and split‐mouth groups, respectively, similar to that in the original NMA. This indicates that the study design is not a significant contributor to the heterogeneity.

**TABLE 2:** Results from the subgroup analysis stratified by study design.

Comparison	Parallel	Split‐mouth	*Q*	*p*
FO:EMD	0.76 (0.38, 1.15)	1.17 (0.69, 1.66)	1.67	0.197
FO:EMD + BG	0.80 (0.30, 1.30)	1.54 (0.37, 2.71)	1.29	0.255
FO:EMD + PRP + BG	0.70 (−1.06, 2.47)	—	—	—
FO:GTR	1.44 (1.04, 1.84)	0.93 (0.40, 1.47)	2.21	0.137
FO:GTR + BG	0.83 (0.28, 1.39)	1.44 (0.37, 2.51)	0.98	0.323
FO:GTR + EMD	1.32 (0.37, 2.28)	1.33 (−0.33, 2.99)	0	0.992
FO:GTR + PRP + BG	0.94 (−0.06, 1.95)	1.81 (0.34, 3.28)	0.91	0.339

## 6 Additional Issues

This section comments on a few practical issues when conducting a meta‐analysis or NMA.

### 6.1 Minimum Number of Studies Included in a meta‐Analysis

By definition, a meta‐analysis requires at least two studies, but more studies are needed to yield a robust estimate of the heterogeneity variance (random‐effects model) [32]. The popular DerSimonian‐Laird method tends to underestimate heterogeneity, so the Hartung‐Knapp method has been recommended to adjust the SE of the pooled effect, assuming a *t*‐distribution rather than a standard normal distribution, as discussed in the section on the Z test for the pooled effect [71]. The Sidik‐Jonkman method proposes an alternative estimator for the heterogeneity when the number of included studies is small and usually yields a larger estimated $\tau^{2}$ [72].

A related issue is whether a meta‐analysis should be conducted at all when substantial heterogeneity across studies makes the pooled effect estimate difficult to interpret. Heterogeneity is not limited to differences in the magnitude of reported effect sizes; studies may also vary in patient populations, administration of interventions, outcome assessment methods, and follow‐up durations. Importantly, choosing not to perform a meta‐analysis does not eliminate the problem of heterogeneity, as qualitative synthesis of highly heterogeneous studies can be equally challenging and potentially more subjective. Rather than avoiding quantitative synthesis altogether, a more constructive approach is to reduce heterogeneity through a more precise PICOS framework and to explore potential sources of heterogeneity using subgroup analyses and meta‐regression.

### 6.2 The Use of Artificial Intelligence in Evidence Synthesis

Growing access to artificial intelligence (AI), particularly large language models (LLMs), has made it an increasingly popular tool for conducting systematic reviews and meta‐analyses [73, 74]. As discussed at the beginning of this tutorial, evidence synthesis comprises a sequence of distinct methodological steps—literature searching, screening articles for eligibility, extracting data, synthesising effect estimates and appraising the certainty of evidence—each with its suitability for automation [73]. For developing and refining search strategies, AI tools have demonstrated reasonable performance and can help identify relevant search terms and sources. During eligibility screening, AI‐assisted approaches generally achieve high sensitivity (87.8%–95.8%) but a wide range of specificity (44.2%–80.9%) [75, 76, 77, 78]; human reviewers therefore remain indispensable for confirming the final set of eligible studies and preventing inadvertent exclusion of relevant trials.

In contrast, AI performs less reliably at data extraction, where errors such as outcome omission and fabrication (hallucination) of numerical values have been reported, limiting its independent use for this step [79, 80]. Such errors are particularly hazardous because they may propagate undetected into quantitative synthesis. Although general‐purpose models can perform meta‐analytic computations, the underlying statistical settings and packages are often unclear and difficult to reproduce; by comparison, established and validated meta‐analysis software offers greater transparency and reproducibility and is preferable for pooling estimates [81, 82]. For risk‐of‐bias and certainty‐of‐evidence appraisal, AI can achieve high agreement rates, particularly in randomised controlled trials [83]. However, while AI may demonstrate substantial agreement with expert judgement as a primary screening tool, final assessment accuracy still requires verification by experienced reviewers.

Taken together, these considerations indicate that AI tools can assist with—and improve the efficiency of—selected stages of systematic reviews and meta‐analyses, but cannot replace the judgement and accountability of trained academic professionals, on whom reproducibility and scientific integrity ultimately depend. Box 5 summarises a critical appraisal of the potential contributions of AI tools at each stage of evidence synthesis.

However, while using AI tools may improve the efficiency of conducting a meta‐analysis, it may also reduce researchers' engagement in the process. Searching and screening the literature is time‐consuming and laborious, but researchers also gain substantial knowledge about their topics of interest in the process. AI tools may achieve comparable accuracy in assessing the risk of bias for each study, but if no one on the research team carefully reads the full texts of the included studies, can the resulting report still be considered a systematic review?

BOX 5 **Stage‐by‐stage appraisal of artificial intelligence (AI)/large language model (LLM) assistance for the stages of evidence synthesis.**

**Table:** 

Stage	AI acceleration potential^a^	Risk level^b^	Key methodological consideration
Literature search strategy	★★★★★	● Low	AI rapidly proposes Boolean strings and keywords but tends to favour precision over recall; search strategies should be peer‐reviewed (ideally with an information specialist) before execution [74, 78].
Study screening (eligibility)	★★★★☆	● Moderate	High sensitivity with variable specificity; suitable for prioritisation and flagging under predefined stopping rules and sampled verification, rather than autonomous exclusion [75, 76, 77].
Data extraction	★☆☆☆☆	● High	Omission and hallucination of numeric values remain common; key figures require dual human verification against the source, item by item [79, 80].
Statistical analysis (meta‐analysis)	★★★☆☆	● Moderate	AI can draft statistical software code and preliminary interpretation, but models and assumptions must be human‐verified; validated meta‐analysis software is preferred, and full reproducibility should be preserved [73, 74].
Risk‐of‐bias appraisal	★★★★★	● Moderate	Useful for a first‐pass draft judgement with substantial agreement against experts, but consistency remains unstable; final judgements rest with reviewers, each retaining a supporting sentence and page reference [74, 81].

References supporting each stage‐specific judgement are shown in brackets. The ratings for AI acceleration potential and risk level represent the authors' synthesis of the available evidence rather than direct estimates from any individual study cited.

^a^AI acceleration potential is rated on a 5‐point scale (★ = minimal, ★★★★★ = substantial time saving), reflecting the relative efficiency gain from AI assistance at each stage.

^b^Risk level denotes the potential for unverified AI output to compromise validity and is shown as a traffic‐light rating: ● high, ● moderate, ● low.

### 6.3 Assessing the Quality of Evidence From Pairwise meta‐Analysis and NMA

#### 6.3.1 Risk of bias Assessment for Individual Studies

The Cochrane Risk of Bias (RoB) tool, developed by the Cochrane Collaboration, is commonly used to assess the internal validity of RCTs included in a systematic review. Its purpose is to evaluate whether the design, conduct, analysis, or reporting of a study could systematically distort the study's estimated treatment effect. The updated RoB 2 tool is more structured and outcome‐specific [84]. It assesses potential biases in the following five domains: (1) bias arising from the randomisation process; (2) bias due to deviations from intended interventions; (3) bias due to missing outcome data; (4) bias in measurement of the outcome; and (5) bias in selection of the reported results. Each domain is judged using signalling questions, leading to an overall judgement of low risk of bias, some concerns, or high risk of bias. The assessment of the included studies for our NMA example on PPD reduction is provided in Table S3 and could be used to assess and interpret the results from a meta‐analysis or NMA.

#### 6.3.2 GRADE

The GRADE (Grading of Recommendations Assessment, Development and Evaluation) approach is commonly used to assess and interpret results from meta‐analyses [85]. The certainty of evidence is scored as high, moderate, low, or very low. Starting with high quality, each pairwise comparison may be downgraded for the following reasons: risk of bias, inconsistency, indirectness, imprecision and publication bias. However, it is more complex when assessing the certainty or quality of evidence for a NMA. In a NMA, the estimates of treatment effects integrate direct and indirect evidence, so the assessment should consider both direct and indirect comparisons. When evaluating the indirectness of indirect evidence, we assess whether each study is representative of the meta‐analysis's target population and whether there is intransitivity, which refers to biased evidence from indirect comparisons. After assessing the direct and indirect evidence, the certainty of the network estimate is the higher score of the direct and indirect certainty unless inconsistency is present, as the higher certainty evidence dominates the estimated effect certainty of the network estimate [86].

#### 6.3.3 CINeMA

CINeMA (Confidence In Network Meta‐Analysis) [87, 88] is an assessment tool for network meta‐analysis implemented in a web application. It comprises six domains: within‐study bias, reporting bias, indirectness, imprecision, heterogeneity and incoherence. For each domain, three levels of concerns are defined: no concerns, some concerns and major concerns, and these judgements are then summarised into a confidence rating, scored as high, moderate, low, or very low [81]. CINeMA assumes that authors have implemented a comprehensive search strategy and obtained the most suitable estimate of treatment effects from the results of studies [88]. Additionally, authors need to assess the study‐level risk of bias and indirectness before conducting the assessment.

The within‐study bias domain refers to concerns about a biased estimated treatment effect. Using the RoB assessment, where codes of 1 to 3 indicate low, moderate, or high risk of bias, a percentage contribution matrix describes how much information each study contributes to the results. Reviewers can use the average, majority, or highest risk‐of‐bias rating in their assessment. ‘Average’ RoB is a weighted average of the bias levels of each study, calculated by multiplying each study's bias level by its percentage contribution. ‘Majority’ RoB uses the largest percentage of the bias level to determine the overall risk of bias level, while ‘highest’ RoB uses the highest level in the included studies [88]. Reporting bias can be assessed using the Risk Of Bias due to Missing Evidence in Network meta‐analysis (ROB‐MEN) framework, which examines whether the systematic review's search contains unpublished articles, or whether included studies are prone to favouring one treatment [89]. Indirectness refers to relevance of included studies to the research question, including definition of the target population, intervention and outcome of interest. Concerns about indirectness also relate to the transitivity assumption in network meta‐analysis, arising from differences in study settings across included studies.

Imprecision refers to uncertainty in the estimated treatment effect, whereas heterogeneity refers to variation in treatment effects across studies. In CINeMA, both domains are assessed using prespecified ranges of equivalence that define clinically important treatment effects. For imprecision, the judgement is based on whether the confidence interval crosses one or both equivalence boundaries on the opposite side of the no effect line as the point estimate. For heterogeneity, the judgement is based on whether the prediction interval crosses these boundaries. No concerns are assigned when the interval lies entirely within a region leading to a single clinical decision; some concerns are assigned when it crosses one equivalence boundary; and major concerns are assigned when it crosses both boundaries, indicating that different clinically relevant conclusions are supported by the data [88].

Finally, under the assumption of transitivity, incoherence refers to consistency of the estimated treatment effect between direct and indirect evidence. This is assessed using a design‐by‐treatment interaction test, where a *p*‐value < 0.05 indicates a ‘major concern’, a *p*‐value between 0.05 and 0.10 indicates ‘some concerns’, and otherwise it is considered ‘no concerns’. The other approach is SIDE (Separating Indirect from Direct Evidence), with a *p*‐value greater than 0.10 indicating ‘no concern’; otherwise, the judgement is based on the location of the confidence intervals for the direct and indirect estimates. The result of the CINeMA assessment is summarised in the report, with the default judgement set at a high level. Reviewers can downgrade the level by considering the result across the six domains.

As an example, we used CINeMA to assess the results from the NMA on PPD reduction. The assessment, shown in Table S4, suggests a very low to low level of confidence in all pairwise network estimates due to ‘Major concerns’ in the domains of Imprecision and Heterogeneity. Imprecision is evaluated using a clinically important mean difference of −1 mm to 1 mm. The 95% confidence intervals crossed the two clinically important values in different directions, which is a major concern for precision. Due to the generally low confidence in the network estimates, we need to interpret the estimates of relative treatment effects and treatment ranking cautiously.

### 6.4 Disconnected or Broken Network

The contrast‐based model in Model (5) requires that all treatments be connected in a network plot, as in Figure 6; otherwise, not all treatments can be compared within the same model. When the network is not fully connected, one solution is to identify additional studies to connect the disconnected pieces of the network. For example, non‐randomised comparative studies may be included to connect the subnetworks. Those studies have, however, lower internal validity, so it is critical to carefully assess their quality to ensure their results are comparable to those from randomised controlled trials.

An alternative is to use component network meta‐analysis [90, 91]. Complex interventions, such as EMD + BG, have multiple components, and under an assumption of additive treatment effects, we can model the effect of EMD + BG as the sum of the separate effects of EMD and BG. Sometimes a component NMA can connect a disconnected network. In our previous network meta‐analysis on the treatment of peri‐implantitis [3], we used component NMA to analyse a disconnected network with four subnetworks. Standard NMA can be viewed as a component NMA with all interactions included.

Another option is to use the baseline or arm‐based model, which do not require all treatments to be connected [38, 92, 93]. These two models make a stronger assumption about exchangeability across trials of treatment effects [39, 92, 94]. Although these models have been used in the literature, caution must be exercised in interpreting their results because of their stronger assumptions. We refer readers to our recent publication for a more detailed discussion about different assumptions of these models [95].

### 6.5 Including Non‐Randomised Comparative Studies or Single‐Arm Studies

In this article's examples, we included only studies in which interventions were randomly assigned to two or more patient groups or, for split‐mouth studies, to locations within a patient's mouth. Under the assumption of exchangeable treatment contrasts, standard meta‐analysis and NMA require that each study have at least two different groups so that treatment contrasts are calculated within each study before they are combined across studies. Excluding studies with non‐random assignment of interventions is mainly due to concerns about their internal validity, that is, whether such observational studies can provide unbiased evidence comparing treatments. For many research questions, observational studies may be the main or only source of evidence, and special tools have been developed to assess their quality and risk of bias.

Standard meta‐analysis and NMA cannot include single‐arm studies because no treatment contrast can be calculated within such a study. As discussed in previous sections, standard meta‐analysis and NMA assume that treatment *contrasts*, not treatment *arms*, are exchangeable across studies. For interventions that have not been compared with others in any randomised trial, it is only possible to assess their relative effectiveness in a meta‐analysis or NMA using the baseline or arm‐based model, as discussed above for disconnected NMAs. These models, however, make strong assumptions that need to be carefully considered when interpreting their results [95].

### 6.6 Exponential Growth in the Number of meta‐Analyses

A quick PubMed search (on 4 June 2026) shows that the number of studies with ‘meta‐analysis’ in the title rose from 422 in 2000 to more than 35 000 in 2025. In the early years of evidence‐based medicine, systematic reviews and meta‐analyses were relatively uncommon and often associated with collaborative efforts, such as those conducted by the Cochrane Collaboration. Over time, advances in electronic databases, statistical software, reporting guidelines and the expansion of online and open‐access publishing have greatly lowered barriers to conducting and publishing meta‐analyses.

The rapid growth of meta‐analyses has both advantages and disadvantages. On the positive side, evidence can be synthesised more quickly and across a wider range of clinical questions. However, multiple meta‐analyses addressing similar topics may reach different conclusions. Such discrepancies can arise because: (1) seemingly minor differences in the PICOS framework may lead to different study eligibility criteria; (2) addition of newly published studies may alter the conclusions of earlier meta‐analyses; (3) different analytical choices, including risk‐of‐bias assessment, effect measures and statistical models, may influence the results; and (4) the large number of meta‐analyses on related topics increases the likelihood of spurious or false‐positive findings.

Consequently, clinicians should not regard all meta‐analyses as equally trustworthy simply because they are often placed near the top of evidence hierarchies. The validity of a meta‐analysis depends on the quality of the underlying studies, the appropriateness of the review question, the completeness of the literature search, the accuracy of data extraction and the suitability of the statistical methods used. Because most meta‐analytic data are presented in tables and figures, readers are encouraged to critically examine each step of the review process. The procedures described in this tutorial provide a practical framework for evaluating the strengths and limitations of published meta‐analyses.

## 7 Conclusions

Meta‐analyses have become indispensable tools for evidence‐based practice because they provide crucial information on the comparative effectiveness of interventions and enhance the quality of decision‐making. This article provides an essential introduction to their assumptions and statistical models, with a special focus on issues related to meta‐analyses in periodontal research. We strongly encourage close collaboration between periodontal researchers and experienced statisticians when planning and conducting meta‐analyses.

## Funding

This work was supported by the National Science and Technology Council in Taiwan (grant no. NSTC 112‐2314‐B‐002‐210‐MY3).

## Conflicts of Interest

The authors declare no conflicts of interest.

## Supporting information


**Data S1:** Supplementary data and R scripts.

**Table S1:** Common errors in pairwise meta‐analysis and network meta‐analysis.

**Table S2:** Probing pocket depth reduction data from 65 trials.

**Table S3:** Quality Assessment of included RCTs for network meta‐analysis.

**Table S4:** CINeMA assessment for the example of network meta‐analysis regarding outcomes of probing pocket depth reduction.

## Data Availability

The data of the two examples that support the findings of this study can be found in online Supporting Information S1.
