# Supplementary material for: A Practical Guide to Evidence Synthesis: Meta‐Analysis, Network Meta‐Analysis, Meta‐Regressions and Beyond
Source: J Periodontal Res. 2026 Jul 25;61(7):847–75. doi: 10.1111/jre.70150 (PMC13665622; doi:10.1111/jre.70150)
Supplement: Supplementary file 1 [file JRE-61-847-s001.docx]

**Supplementary Table S4:** CINeMA assessment for the example of network meta-analysis regarding outcomes of probing pocket depth reduction.

| **Comparison** | **Number of studies** | **Within- study bias** | **Reporting bias** | **Indirectness** | **Imprecision*** | **Heterogeneity*** | **Incoherence** | **Confidence rating** |
| --- | --- | --- | --- | --- | --- | --- | --- | --- |
| **Mixed evidence** | | | | | | | | |
| FO vs EMD | 19 | Some concerns | Some concerns | No concerns | No concerns | Some concerns | No concerns | Low |
| FO vs EMD+BG | 4 | Some concerns | Some concerns | No concerns | No concerns | Some concerns | No concerns | Low |
| FO vs GTR | 18 | Some concerns | Some concerns | No concerns | No concerns | Some concerns | No concerns | Low |
| FO vs GTR+EMD | 1 | Some concerns | Some concerns | No concerns | No concerns | Some concerns | No concerns | Low |
| FO vs GTR+BG | 6 | Some concerns | Some concerns | No concerns | No concerns | Some concerns | No concerns | Low |
| EMD vs EMD+BG | 13 | Some concerns | Some concerns | No concerns | No concerns | Major concerns | No concerns | Very low |
| EMD vs GTR | 8 | Some concerns | Some concerns | No concerns | No concerns | Some concerns | No concerns | Low |
| EMD vs GTR+EMD | 3 | Some concerns | Some concerns | No concerns | Some concerns | Some concerns | No concerns | Very low |
| EMD+BG vs EMD+PRP+BG | 1 | Some concerns | Some concerns | No concerns | Major concerns | No concerns | No concerns | Very low |
| EMD+BG vs GTR+BG | 2 | Some concerns | Some concerns | No concerns | No concerns | Major concerns | No concerns | Very low |
| GTR vs GTR+EMD | 2 | Some concerns | Some concerns | No concerns | No concerns | Major concerns | No concerns | Very low |
| GTR vs GTR+BG | 4 | Some concerns | Some concerns | No concerns | No concerns | Major concerns | No concerns | Very low |
| GTR+BG vs GTR+PRP+BG | 5 | Some concerns | Some concerns | No concerns | No concerns | Major concerns | No concerns | Very low |
| **Indirect evidence** | | | | | | | | |
| FO vs EMD+PRP+BG | 0 | Some concerns | Some concerns | No concerns | Some concerns | Some concerns | No concerns | Very low |
| FO vs GTR+PRP+BG | 0 | Some concerns | Some concerns | No concerns | No concerns | Some concerns | No concerns | Low |
| EMD vs EMD+PRP+BG | 0 | Some concerns | Some concerns | No concerns | Major concerns | No concerns | No concerns | Very low |
| EMD vs GTR+BG | 0 | Some concerns | Some concerns | No concerns | No concerns | Major concerns | No concerns | Very low |
| EMD vs GTR+PRP+BG | 0 | Some concerns | Some concerns | No concerns | Some concerns | Some concerns | No concerns | Very low |
| EMD+BG vs GTR | 0 | Some concerns | Some concerns | No concerns | No concerns | Major concerns | No concerns | Very low |
| EMD+BG vs GTR+EMD | 0 | Some concerns | Some concerns | No concerns | Some concerns | Some concerns | No concerns | Very low |
| EMD+BG vs GTR+PRP+BG | 0 | Some concerns | Some concerns | No concerns | Some concerns | Some concerns | No concerns | Very low |
| EMD+PRP+BG vs GTR | 0 | Some concerns | Some concerns | No concerns | Major concerns | No concerns | No concerns | Very low |
| EMD+PRP+BG vs GTR+EMD | 0 | Some concerns | Some concerns | No concerns | Major concerns | No concerns | No concerns | Very low |
| EMD+PRP+BG vs GTR+BG | 0 | Some concerns | Some concerns | No concerns | Major concerns | No concerns | No concerns | Very low |
| EMD+PRP+BG vs GTR+PRP+BG | 0 | Some concerns | Some concerns | No concerns | Major concerns | No concerns | No concerns | Very low |
| GTR vs GTR+PRP+BG | 0 | Some concerns | Some concerns | No concerns | No concerns | Major concerns | No concerns | Very low |
| GTR+EMD vs GTR+BG | 0 | Some concerns | Some concerns | No concerns | Some concerns | Some concerns | No concerns | Very low |
| GTR+EMD vs GTR+PRP+BG | 0 | Some concerns | Some concerns | No concerns | Major concerns | No concerns | No concerns | Very low |

*Imprecision and heterogeneity are evaluated with a clinically important mean difference between -1mm and 1mm. The 95% confidence intervals and prediction interval crossed the two clinically important values in different directions, which is a major concern for the precision and heterogeneity, respectively.

*Abbreviations***:** BG, bone graft; EMD, enamel matrix derivatives; FO, flap operation; GTR, guided tissue regeneration; PRP, platelet-rich plasma.
