# Supplementary material for: A Practical Guide to Evidence Synthesis: Meta‐Analysis, Network Meta‐Analysis, Meta‐Regressions and Beyond
Source: J Periodontal Res. 2026 Jul 25;61(7):847–75. doi: 10.1111/jre.70150 (PMC13665622; doi:10.1111/jre.70150)
Supplement: Supplementary file 2 [file JRE-61-847-s002.docx]

**Supplementary Table S1. Common errors in pairwise meta-analysis and network meta-analysis**

| **Stage** | **Common error** | **Consequence** | **Recommended practice** |
| --- | --- | --- | --- |
| Data extraction | Confusing SD with SE | Incorrect study weights; artificially precise estimates | Verify reported measures and convert SE to SD when necessary |
| Data extraction | Ignoring within-person correlation in split-mouth studies | Overestimated uncertainty | Adjust variance using appropriate correlation assumptions |
| Data extraction | Ignoring the clustering of teeth within patients | Inflated effective sample size | Use an effective sample size accounting for the intra-class correlation or use cluster-adjusted estimates |
| Effect size selection | Pooling outcomes measured on the same scales using SMD | Lacks clinical interpretation | Use MD when outcomes are measured on the same scale across studies |
| Effect size selection | Pooling outcomes measured on different scales using MD | Non-comparable estimates | Use SMD when appropriate |
| Model selection | Choosing common-effect or random-effects model solely according to $I^{2}$ | Model misspecification | Choice is based on scientific assumptions about treatment effects |
| Heterogeneity | Interpreting $I^{2}=0\%$ as absence of heterogeneity | Overconfidence in results | Recognize that $I^{2}$ is an imperfect measure, especially with few studies |
| Interpretation | Pooling absolute treatment effects across studies | Biased conclusions | Pool treatment contrasts, not absolute outcomes |
| Publication bias | Interpreting funnel plot asymmetry as definitive publication bias | Misleading conclusions | Consider alternative explanations such as heterogeneity or chance |
| Meta-regression | Using study-level covariates to infer patient-level effects | Ecological bias | Interpret meta-regression cautiously |
| NMA assumptions | Ignoring transitivity | Invalid indirect comparisons | Assess distributions of potential effect modifiers across comparisons |
| NMA assumptions | Ignoring inconsistency between direct and indirect evidence | Biased network estimates | Evaluate consistency using statistical methods and consider clinical heterogeneities in direct and indirect evidence |
| NMA design | Combining clinically distinct interventions into a single node | Violation of homogeneity/transitivity | Define treatment nodes carefully |
| NMA interpretation | Focusing only on treatment rankings (SUCRA/P-scores) | Overinterpretation of trivial differences | Emphasize effect estimates and uncertainty, and consider clinical relevance |
