# Supplementary material for: A Practical Guide to Evidence Synthesis: Meta‐Analysis, Network Meta‐Analysis, Meta‐Regressions and Beyond
Source: J Periodontal Res. 2026 Jul 25;61(7):847–75. doi: 10.1111/jre.70150 (PMC13665622; doi:10.1111/jre.70150)
Supplement: Supplementary file 3 [file JRE-61-847-s003.docx]

**Supplementary Appendix Table S2**: Probing pocket depth reduction data from 65 trials.

| study | trtname | y | n | t | g | sd | year | b.PPD |
| --- | --- | --- | --- | --- | --- | --- | --- | --- |
| 1 | Flap op (1) | 3.7 | 14 | 1 | 0 | 1.40 | 2001 | 8.6 |
| 1 | EMD+24%EDTA (2) | 4.1 | 14 | 2 | 0 | 1.70 | 2001 | 8.4 |
| 1 | GTR(Resolut) (5) | 4.2 | 14 | 5 | 0 | 1.90 | 2001 | 8.4 |
| 1 | EMD+GTR(Resolut) (6) | 4.3 | 14 | 6 | 0 | 1.40 | 2001 | 8.6 |
| 2 | Flap op (1) | 3.8 | 18 | 1 | 0 | 1.80 | 2006 | 9.8 |
| 2 | EMD+24%EDTA (2) | 3.9 | 19 | 2 | 0 | 1.30 | 2006 | 8.6 |
| 2 | EMD+24%EDTA+Cerasorb(TCP) (3) | 4.1 | 19 | 3 | 0 | 1.20 | 2006 | 8.6 |
| 3 | EMD+24%EDTA+Bio-Oss (3) | 5.9 | 13 | 3 | 0 | 1.30 | 2008 | 8.8 |
| 3 | EMD+24%EDTA+Bio-Oss+PRP (4) | 5.8 | 13 | 4 | 0 | 1.80 | 2008 | 8.8 |
| 4 | Flap op (1) | 2.57 | 12 | 1 | 0 | 1.27 | 2004 | 6.71 |
| 4 | EMD+24%EDTA (2) | 4.71 | 12 | 2 | 0 | 1.60 | 2004 | 7.86 |
| 5 | Flap op (1) | 3 | 46 | 1 | 0 | 1.47 | 2005 | 7.11 |
| 5 | EMD+24%EDTA (2) | 4 | 64 | 2 | 0 | 2.01 | 2005 | 8.06 |
| 6 | EMD+37%orthophosphoric acid gel+GTR(Tissue Guide) (6) | 4.3 | 21 | 6 | 0 | 1.60 | 2002 | 7.9 |
| 6 | EMD+37%orthophosphoric acid gel (2) | 3.8 | 17 | 2 | 0 | 0.90 | 2002 | 7.8 |
| 6 | GTR(Tissue Guide) (5) | 3.7 | 23 | 5 | 0 | 1.20 | 2002 | 7 |
| 7 | EMD+24%EDTA (2) | 4.5 | 15 | 2 | 0 | 2.00 | 2005 | 8.5 |
| 7 | EMD+24%EDTA+Perioglass (3) | 4.2 | 15 | 3 | 0 | 1.40 | 2005 | 8.5 |
| 8 | Flap op (1) | 1.4 | 10 | 1 | 0 | 1.26 | 2000 | 7.7 |
| 8 | EMD+17%EDTA (2) | 4.9 | 10 | 2 | 0 | 1.79 | 2000 | 7.7 |
| 8 | GTR(ePTFE) (5) | 5.7 | 10 | 5 | 0 | 1.06 | 2000 | 8.1 |
| 9 | Flap op+24%EDTA (1) | 3.3 | 83 | 1 | 0 | 1.70 | 2002 | 7.7 |
| 9 | EMD+24%EDTA (2) | 3.9 | 83 | 2 | 0 | 1.70 | 2002 | 8 |
| 10 | Flap op+24%EDTA (1) | 4.5 | 30 | 1 | 0 | 1.00 | 2002 | 8.9 |
| 10 | EMD+24%EDTA (2) | 5.1 | 30 | 2 | 0 | 0.70 | 2002 | 9.2 |
| 10 | GTR(Ti-ePTFE) (5) | 6.5 | 30 | 5 | 0 | 1.60 | 2002 | 8.9 |
| 11 | EMD+24%EDTA (2) | 5.8 | 30 | 2 | 0 | 0.80 | 2003 | 9.2 |
| 11 | EMD+24%EDTA+Bio-Oss (3) | 6.2 | 30 | 3 | 0 | 0.40 | 2003 | 9.4 |
| 12 | EMD+24%EDTA (2) | 5.6 | 14 | 2 | 0 | 1.70 | 2007 | 9.6 |
| 12 | EMD+24%EDTA+Autogenous bone grafts (3) | 5.1 | 13 | 3 | 0 | 1.70 | 2007 | 9.1 |
| 13 | Flap op (1) | 3.9 | 15 | 1 | 0 | 1.00 | 2009 | 8.5 |
| 13 | EMD+24%EDTA (2) | 4.2 | 15 | 2 | 0 | 1.60 | 2009 | 8.7 |
| 14 | EMD+24%EDTA (2) | 4.6 | 20 | 2 | 0 | 0.40 | 2010 | 8.2 |
| 14 | EMD+24%EDTA+Autogenous bone grafts (3) | 5.6 | 20 | 3 | 0 | 0.90 | 2010 | 8.4 |
| 15 | EMD+24%EDTA (2) | 3.2 | 19 | 2 | 0 | 1.30 | 2008 | 6.6 |
| 15 | GTR(ePTFE) (5) | 3.7 | 20 | 5 | 0 | 1.20 | 2008 | 7.2 |
| 16 | EMD+24%EDTA (2) | 5.3 | 48 | 2 | 0 | 1.90 | 2003 | 8.5 |
| 16 | GTR(ePTFE) (5) | 5.6 | 48 | 5 | 0 | 1.50 | 2003 | 8.1 |
| 17 | EMD+24%EDTA (2) | 3.8 | 35 | 2 | 0 | 1.50 | 2004 | 7.9 |
| 17 | GTR(Guidor) (5) | 3.3 | 32 | 5 | 0 | 1.50 | 2004 | 8 |
| 18 | Flap op (1) | 3.2 | 62 | 1 | 0 | 1.50 | 2004 | 7.9 |
| 18 | GTR(Bio-Gide)+Bio-Oss (7) | 3.7 | 62 | 7 | 0 | 1.80 | 2004 | 7.8 |
| 19 | Flap op (1) | 3.6 | 54 | 1 | 0 | 2.10 | 2001 | 8.2 |
| 19 | GTR(Guidor) (5) | 4.4 | 55 | 5 | 0 | 2.40 | 2001 | 8.2 |
| 20 | Flap op (1) | 2.5 | 18 | 1 | 0 | 1.90 | 1998 | 7.8 |
| 20 | GTR(Guidor) (5) | 2.9 | 20 | 5 | 0 | 1.80 | 1998 | 8.2 |
| 21 | Flap op (1) | 3.09 | 71 | 1 | 0 | 1.67 | 1998 | 7.3 |
| 21 | GTR(Resolut) (5) | 4.03 | 72 | 5 | 0 | 1.81 | 1998 | 8.3 |
| 22 | GTR(ePTFE)+Bio-Oss (7) | 5.7 | 12 | 7 | 0 | 1.20 | 2007 | 8.8 |
| 22 | GTR(ePTFE)+Bio-Oss+PRP (8) | 5.5 | 12 | 8 | 0 | 1.20 | 2007 | 8.6 |
| 23 | GTR(ePTFE)+TCP (7) | 5.4 | 14 | 7 | 0 | 0.70 | 2008 | 9 |
| 23 | GTR(ePTFE)+TCP+PRP (8) | 5.8 | 14 | 8 | 0 | 0.60 | 2008 | 9.1 |
| 24 | GTR(ePTFE)+Bio-Oss (7) | 5.5 | 15 | 7 | 0 | 1.70 | 2007 | 8.8 |
| 24 | GTR(ePTFE)+Bio-Oss+PRP (8) | 5.5 | 15 | 8 | 0 | 1.30 | 2007 | 8.7 |
| 25 | Flap op (1) | 3.6 | 16 | 1 | 0 | 1.30 | 2005 | 8 |
| 25 | GTR(Bio-Gide)+Bio-Oss (7) | 5.4 | 16 | 7 | 0 | 0.90 | 2005 | 8.3 |
| 26 | Flap op (1) | 3.8 | 14 | 1 | 0 | 1.80 | 2003 | 9 |
| 26 | GTR(Bio-Gide)+Bio-Oss (7) | 5.3 | 14 | 7 | 0 | 1.60 | 2003 | 9.2 |
| 27 | GTR(Guidor) (5) | 4.12 | 14 | 5 | 0 | 0.84 | 2000 | 7.93 |
| 27 | GTR(Guidor)+DFDBA (7) | 3.37 | 16 | 7 | 0 | 1.16 | 2000 | 7.34 |
| 28 | Flap op (1) | 2.8 | 17 | 1 | 0 | 0.73 | 2008 | 7.2 |
| 28 | GTR(Collagen) (5) | 5.2 | 17 | 5 | 0 | 1.09 | 2008 | 8 |
| 29 | Flap op (1) | 4.3 | 12 | 1 | 0 | 1.73 | 1996 | 8.5 |
| 29 | GTR(ePTFE);GTR(Resolute) (5) | 6.2 | 24 | 5 | 0 | 1.72 | 1996 | 9.2 |
| 30 | Flap op (1) | 4.6 | 15 | 1 | 0 | 1.76 | 1995 | 8.3 |
| 30 | GTR(ePTFE);GTR(Ti-ePTFE) (5) | 5.9 | 30 | 5 | 0 | 2.19 | 1995 | 8.3 |
| 31 | Flap op (1) | 2.9 | 15 | 1 | 0 | 1.74 | 2003 | 7.9 |
| 31 | GTR(Resolut-XT) (5) | 3.9 | 14 | 5 | 0 | 1.56 | 2003 | 8.9 |
| 31 | GTR(Resolut-XT)+Bio-Oss (7) | 3.8 | 15 | 7 | 0 | 1.52 | 2003 | 8.7 |
| 32 | Flap op (1) | 4.4 | 15 | 1 | 0 | 1.60 | 2011 | 7.5 |
| 32 | EMD+24%EDTA (2) | 4.4 | 15 | 2 | 0 | 1.20 | 2011 | 7.8 |
| 32 | EMD+24%EDTA+Bio-Oss (3) | 4 | 15 | 3 | 0 | 1.30 | 2011 | 7.3 |
| 33 | EMD+24%EDTA (2) | 2.9 | 20 | 2 | 0 | 2.10 | 2011 | 9.4 |
| 33 | GTR(Ti-ePTFE) (5) | 5.5 | 20 | 5 | 0 | 1.00 | 2011 | 9.6 |
| 34 | EMD+24%EDTA (2) | 2.9 | 35 | 2 | 0 | 1.80 | 2011 | 7.1 |
| 34 | EMD+24%EDTA+Bone Ceramic (3) | 2.8 | 38 | 3 | 0 | 2.10 | 2011 | 6.9 |
| 35 | Flap op (1) | 1.51 | 10 | 1 | 1 | 0.57 | 1990 | NA |
| 35 | GTR(Collagen) (5) | 1.99 | 10 | 5 | 1 | 0.28 | 1990 | NA |
| 35 | GTR(Collagen)+DFDBA (7) | 2.73 | 10 | 7 | 1 | 0.28 | 1990 | NA |
| 36 | EMD+24%EDTA (2) | 2.86 | 11 | 2 | 1 | 0.52 | 2005 | 6.95 |
| 36 | EMD+24%EDTA+GTR(ePTFE) (6) | 3.02 | 11 | 6 | 1 | 1.45 | 2005 | 7.32 |
| 37 | Flap op (1) | 2.24 | 23 | 1 | 1 | 0.82 | 2001 | 7.32 |
| 37 | EMD+citric acid (2) | 4.94 | 23 | 2 | 1 | 0.53 | 2001 | 7.99 |
| 38 | Flap op+PGA (1) | 2.6 | 30 | 1 | 1 | 1.01 | 1997 | 7.8 |
| 38 | EMD (2) | 3.3 | 30 | 2 | 1 | 1.24 | 1997 | 7.8 |
| 39 | Flap op+PGA+37%orthophosphoric acid gel (1) | 2.22 | 16 | 1 | 1 | 0.68 | 2000 | 6.22 |
| 39 | EMD+37%orthophosphoric acid gel (2) | 3 | 16 | 2 | 1 | 0.86 | 2000 | 6.33 |
| 40 | Flap op+PGA (1) | 3.5 | 10 | 1 | 1 | 1.11 | 1999 | 7.9 |
| 40 | EMD+24%EDTA (2) | 4.4 | 10 | 2 | 1 | 0.88 | 1999 | 8 |
| 41 | Flap op+24%EDTA (1) | 4.39 | 14 | 1 | 1 | 1.29 | 2005 | 7.38 |
| 41 | EMD+24%EDTA (2) | 4.17 | 14 | 2 | 1 | 1.39 | 2005 | 7.57 |
| 42 | Flap op (1) | 3.49 | 10 | 1 | 1 | 1.04 | 2010 | 6.13 |
| 42 | EMD+24%EDTA (2) | 4 | 9 | 2 | 1 | 0.84 | 2010 | 6.3 |
| 43 | Flap op (1) | 2.31 | 25 | 1 | 1 | 1.41 | 2002 | 7.404 |
| 43 | GTR(Guidor) (5) | 2.41 | 25 | 5 | 1 | 1.45 | 2002 | 7.504 |
| 44 | Flap op (1) | 1.44 | 20 | 1 | 1 | 1.06 | 1995 | 6.68 |
| 44 | GTR(ePTFE) (5) | 2.67 | 20 | 5 | 1 | 1.48 | 1995 | 6.68 |
| 45 | Flap op (1) | 4.45 | 11 | 1 | 1 | 2.21 | 2000 | 8.6 |
| 45 | GTR(Guidor) (5) | 4.23 | 11 | 5 | 1 | 1.49 | 2000 | 8.4 |
| 46 | Flap op (1) | 3 | 23 | 1 | 1 | 1.18 | 1998 | 6.7 |
| 46 | GTR(Guidor) (5) | 4.3 | 23 | 5 | 1 | 2.10 | 1998 | 7.4 |
| 48 | GTR(Resolut XT)+24%EDTA+TCP (7) | 6 | 25 | 7 | 1 | 0.94 | 2006 | 9.9 |
| 48 | GTR(Resolut XT) +24%EDTA+TCP+PRP (8) | 6.3 | 25 | 8 | 1 | 1.05 | 2006 | 9.9 |
| 50 | Flap op (1) | 3.4 | 30 | 1 | 1 | 1.25 | 1999 | 7.4 |
| 50 | GTR(Guidor, Resolut, ePTFE) (5) | 4.5 | 30 | 5 | 1 | 1.03 | 1999 | 7.8 |
| 51 | Flap op (1) | 3.55 | 10 | 1 | 1 | 0.92 | 1996 | 7.8 |
| 51 | GTR(ePTFE) (5) | 5.35 | 10 | 5 | 1 | 1.15 | 1996 | 7.95 |
| 52 | Flap op (1) | 2.4 | 19 | 1 | 1 | 1.13 | 2009 | NA |
| 52 | EMD+24%EDTA (2) | 4.2 | 19 | 2 | 1 | 1.13 | 2009 | NA |
| 53 | GTR(ePTFE) & GTR(ePTFE) +Doxycyclin (5) | 3.85 | 14 | 5 | 1 | 1.06 | 1995 | 9.75 |
| 53 | GTR(ePTFE)+Doxycyclin+DFDBA (7) | 4.3 | 7 | 7 | 1 | 2.06 | 1995 | 10.6 |
| 54 | EMD (2) | 4.7 | 12 | 2 | 0 | 0.87 | 2012 | 8.8 |
| 54 | EMD+BG(biphasic calcium phosphate) (3) | 4.5 | 12 | 3 | 0 | 1.31 | 2012 | 8.8 |
| 55 | Flap op+24%EDTA (1) | 2.22 | 15 | 1 | 1 | 0.08 | 2013 | 6.82 |
| 55 | Flap op+EDTA(24%)+EMD (2) | 4.12 | 15 | 2 | 1 | 1.09 | 2013 | 7.24 |
| 56 | Flap op (1) | 2.58 | 34 | 1 | 1 | 0.48 | 2013 | 8.7 |
| 56 | Flap op+EMD (2) | 3.51 | 34 | 2 | 1 | 0.50 | 2013 | 8.73 |
| 56 | Flap op+EMD+BG(HA/b-TCP) (3) | 4 | 34 | 3 | 1 | 0.37 | 2013 | 8.79 |
| 57 | EMD (2) | 3.26 | 23 | 2 | 0 | 0.73 | 2014 | 6.56 |
| 57 | EMD+BG(FDBA or DFDBA) (3)*** | 4.02 | 44 | 3 | 0 | 0.86 | 2014 | 6.5 |
| 58 | IPR+EMD+DBBM (3) | 4.9 | 10 | 3 | 0 | 1.20 | 2016 | 8.2 |
| 58 | GTR+DBBM (7) | 4.7 | 10 | 7 | 0 | 2.36 | 2016 | 7.8 |
| 59 | EMD+DBBM+(24%EDTA gel) (3) | 4.6 | 20 | 3 | 0 | 1.90 | 2014 | 8.2 |
| 59 | GTR(Collagen membrane)+DBBM (7) | 4.4 | 20 | 7 | 0 | 1.70 | 2014 | 8.1 |
| 60 | EMD (2) | 3.3 | 23 | 2 | 0 | 1.89 | 2017 | 7.88 |
| 60 | EMD/BC (biphasic calcium phosphate) (3) | 3.14 | 21 | 3 | 0 | 1.95 | 2017 | 7.95 |
| 61 | EMD+BG (Xenograft) (3) | 6.83 | 12 | 3 | 0 | 0.81 | 2022 | 9.5 |
| 61 | EMD (2) | 8.25 | 12 | 2 | 0 | 2.70 | 2022 | 10.75 |
| 62 | EMD+BG (Xenograft) (3) | 6.5 | 15 | 3 | 0 | 2.65 | 2020 | 9.33 |
| 62 | Flap op (1) | 6.2 | 15 | 1 | 0 | 1.33 | 2020 | 9.26 |
| 63 | EMD (2) | 2.3 | 20 | 2 | 0 | 1.20 | 2020 | 6.7 |
| 63 | Flap op (1) | 1.9 | 20 | 1 | 0 | 1.50 | 2020 | 6.7 |
| 64 | EMD (2) | 4.9 | 19 | 2 | 0 | 1.44 | 2025 | 8.15 |
| 64 | EMD+BG (Xenograft) (3) | 5.21 | 21 | 3 | 0 | 1.02 | 2025 | 8.5 |
| 65 | Flap op (1) | 2.5 | 15 | 1 | 0 | 1.22 | 2022 | 6.63 |
| 65 | GTR(Biogide)+BG (Bio-Oss) (7) | 2.31 | 16 | 7 | 0 | 1.47 | 2022 | 6.63 |
| 66 | GTR(Collagen)+BG+PRP (8) | 3.05 | 14 | 8 | 1 | 0.79 | 2021 | 5.3 |
| 66 | GTR(Collagen)+BG (7) | 2.6 | 14 | 7 | 1 | 0.93 | 2021 | 5.4 |
| 67 | GTR (resorbable) (5) | 4.63 | 30 | 5 | 1 | 0.53 | 2021 | 7.6 |
| 67 | Flap op (1) | 3.37 | 30 | 1 | 1 | 0.91 | 2021 | 7.4 |
