# Supplementary material for: A Practical Guide to Evidence Synthesis: Meta‐Analysis, Network Meta‐Analysis, Meta‐Regressions and Beyond
Source: J Periodontal Res. 2026 Jul 25;61(7):847–75. doi: 10.1111/jre.70150 (PMC13665622; doi:10.1111/jre.70150)
Supplement: Supplementary file 4 [file JRE-61-847-s004.docx]

**Supplementary Data and R Scripts**

**Data for pairwise meta-analysis**

| study | year | id | treatment1 | ndefect1 | npatient1 | dppd1 | sddppd1 | dcal1 | sddcal1 | treatment2 | ndefect2 | npatient2 | dppd2 | sddppd2 | dcal2 | sddcal2 | design | group | month |
| --- | --- | --- | --- | --- | --- | --- | --- | --- | --- | --- | --- | --- | --- | --- | --- | --- | --- | --- | --- |
| Sculean et al. | 2001 | 38 | EMD | 12 | 12 | 4.3 | 1.0247 | 3.4 | 1.18322 | GTR (Resorbable Membrane) | 12 | 12 | 4.5 | 1.14017 | 3.2 | 1.41421 | Split-mouth | EMD vs GTR | 12 |
| Sculean et al. | 2001 | 39 | EMD | 14 | 14 | 4.1 | 1.7 | 3.4 | 1.5 | GTR (Resorbable Membrane) | 14 | 14 | 4.2 | 1.9 | 3.1 | 1.5 | Parallel | EMD vs GTR | 12 |
| Minabe et al. | 2002 | 40 | EMD | 22 | 17 | 3.8 | 0.9 | 2.6 | 1 | GTR (Resorbable Membrane) | 23 | 23 | 3.7 | 1.2 | 2.8 | 0.9 | Parallel | EMD vs GTR | 12 |
| Sanz et al. | 2004 | 41 | EMD | 35 | 35 | 3.8 | 1.5 | 3.1 | 1.8 | GTR (Resorbable Membrane) | 32 | 32 | 3.3 | 1.5 | 2.5 | 1.9 | Parallel | EMD vs GTR | 12 |
| Cortellini et al. | 2005 | 42 | EMD | 10 | 10 |  |  | 5.9 | 1.5 | GTR (Resorbable Membrane) | 7 | 7 |  |  | 5.9 | 1.2 | Parallel | EMD vs GTR | 12 |
| Silvestri et al. | 2000 | 43 | EMD | 10 | 10 | 4.8 | 1.6 | 4.5 | 1.6 | GTR (Non-Resorbable Membrane) | 10 | 10 | 5.9 | 1.1 | 4.8 | 2.1 | Parallel | EMD vs GTR | 12 |
| Zucchelli et al. | 2002 | 44 | EMD | 30 | 30 | 5.1 | 0.7 | 4.2 | 0.9 | GTR (Non-Resorbable Membrane) | 30 | 30 | 6.5 | 1.6 | 4.9 | 1.6 | Parallel | EMD vs GTR | 12 |
| Silvestri et al. | 2003 | 45 | EMD | 49 | 49 | 5.3 | 1.9 | 4.1 | 1.8 | GTR (Non-Resorbable Membrane) | 49 | 49 | 5.6 | 1.5 | 4.3 | 1.9 | Parallel | EMD vs GTR | 12 |
| Cortellini et al. | 2005 | 46 | EMD | 10 | 10 |  |  | 5.9 | 1.5 | GTR (Non-Resorbable Membrane) | 12 | 12 |  |  | 6.8 | 2.2 | Parallel | EMD vs GTR | 12 |
| Crea et al. | 2008 | 47 | EMD | 19 | 19 | 3.5 | 1.3 | 2.8 | 1.3 | GTR (Non-Resorbable Membrane) | 20 | 20 | 3.7 | 1.2 | 2.7 | 1.2 | Parallel | EMD vs GTR | 12 |
| Iorio-Siciliano et al. | 2011 | 48 | EMD | 20 | 20 | 2.9 | 2.1 | 2.4 | 2.2 | GTR (Non-Resorbable Membrane) | 20 | 20 | 5.5 | 1 | 4.1 | 1.4 | Parallel | EMD vs GTR | 12 |
| Pontoriero et al. | 1999 | 49 | EMD | 10 | 10 | 4.4 | 1.05 | 3 | 1 | GTR (Resorbable + Non-Resorbable Membranes) | 30 | 30 | 4.5 | 1.4 | 3.1 | 1.3 | Parallel | EMD vs GTR | 12 |

**R script for pairwise meta-analysis**

```
## R code for EMD project
# Call package to read data
library("readxl")
infra <- read_excel("Intrabony_defects_JPR.xlsx",sheet = "infra_wide")
infra <- data.frame(infra)
infra$subgroup <- with(infra, paste(treatment1, treatment2,sep = " vs "))

# Call package for meta-analysis
library(meta)

## EMD vs GTR without subgroups
emd.gtr <- subset(infra, group == "EMD vs GTR")
emd.gtr <- na.omit(emd.gtr)

# metagen => Six variables
emd.gtr.ma <- metacont(n.e = npatient2, mean.e = dppd2, sd.e = sddppd2,
                       n.c = npatient1, mean.c = dppd1, sd.c = sddppd1,
                       studlab = paste(study, year), method.tau = "DL", sm = "MD",
                       label.e = "GTR     ",  # Custom label for experimental
                       label.c = "EMD     ",      # Custom label for control
                       data = emd.gtr)
summary(emd.gtr.ma)


### Forest plot
pdf(file="Forest plot for EMD vs GTR_PPD.pdf",width=12,height=8,paper='special')
forest(emd.gtr.ma, prediction = F, digits.sd = 2, sep.subgroup = ": ",
       label.left = "Favours EMD",
       label.right = "Favours GTR",pooled.totals = FALSE)
dev.off()

### Contour-enhanced funnel plot
# Contour-enhanced funnel plot
pdf(file="Funnel plot for EMD vs GTR_PPD.pdf",width=8,height=6,paper='special')
funnel(emd.gtr.ma, random = F, contour.levels = c(0.9,0.95,0.99),
       col.contour = c("gray50","gray70","gray90"))
legend("topright", c("p < 0.01", "0.01 < p < 0.05", "0.05 < p < 0.10", "p > 0.10"),
       fill=c("gray90","gray70","gray50", 'white'), bg = 'white')
title ("EMD vs GTR")
dev.off()

### Egger's test
# Egger's test
emd.gtr.ma <- metacont(n.e = npatient2, mean.e = dppd2, sd.e = sddppd2,
                       n.c = npatient1, mean.c = dppd1, sd.c = sddppd1,
                       studlab = paste(study, year), method.tau = "DL", sm = "MD",
                       data = emd.gtr)

pdf(file="Egger plot for EMD vs GTR_PPD.pdf",width=8,height=6,paper='special')
emd.gtr.ma.bias <- metabias(emd.gtr.ma,method.bias="Egger", plotit=T, k.min=3) # T for TRUE
emd.gtr.ma.bias
abline(h=c(0,-qt(0.975,emd.gtr.ma.bias$df),qt(0.975,emd.gtr.ma.bias$df)),
       col = c("blue","gray","gray"), lwd=2, lty=c(1,2,2))
arrows(0,emd.gtr.ma.bias$estimate[1]-qt(0.975,emd.gtr.ma.bias$df)*emd.gtr.ma.bias$estimate[2],
       0,emd.gtr.ma.bias$estimate[1]+qt(0.975,emd.gtr.ma.bias$df)*emd.gtr.ma.bias$estimate[2],
       length=0.1,code=3, angle = 90, lwd=2,col="red")
title ("EMD vs GTR")
dev.off()

# Run a meta-regression with membrane types 'subgroup' as a covariate
emd.gtr.metareg.model <- metareg(emd.gtr.ma, ~ subgroup)

# View the results
summary(emd.gtr.metareg.model)

# subgroup analysis
emd.gtr.ma.sub <- metacont(n.e = npatient2, mean.e = dppd2, sd.e = sddppd2,
                       n.c = npatient1, mean.c = dppd1, sd.c = sddppd1,
                       studlab = paste(study, year), method.tau = "DL", sm = "MD",
                       label.e = "GTR     ",  # Custom label for experimental
                       label.c = "EMD     ",      # Custom label for control
                       data = emd.gtr, subgroup = subgroup)
summary(emd.gtr.ma.sub)

### Forest plot
pdf(file="Subgroup forest plot for EMD vs GTR_PPD.pdf",width=12,height=8,paper='special')
forest(emd.gtr.ma.sub, prediction = T, digits.sd = 2, sep.subgroup = ": ",
       label.left = "Favours EMD",
       label.right = "Favours GTR",pooled.totals = FALSE)
dev.off()
```

**Data for network meta-analysis**

| study | authoryr | trtname | y | sd.unadj | n | t | g | sd | year | base.PPD | base.intra.def |
| --- | --- | --- | --- | --- | --- | --- | --- | --- | --- | --- | --- |
| 1 | Sculean 2001b | Flap op (1) | 3.7 | 1.4 | 14 | 1 | 0 | 1.4 | 2001 | 8.6 | 3.9 |
| 1 | Sculean 2001b | EMD+24%EDTA (2) | 4.1 | 1.7 | 14 | 2 | 0 | 1.7 | 2001 | 8.4 | 3.8 |
| 1 | Sculean 2001b | GTR(Resolut) (5) | 4.2 | 1.9 | 14 | 5 | 0 | 1.9 | 2001 | 8.4 | 3.7 |
| 1 | Sculean 2001b | EMD+GTR(Resolut) (6) | 4.3 | 1.4 | 14 | 6 | 0 | 1.4 | 2001 | 8.6 | 4 |
| 2 | Bokan 2006 | Flap op (1) | 3.8 | 1.8 | 18 | 1 | 0 | 1.8 | 2006 | 9.8 | NA |
| 2 | Bokan 2006 | EMD+24%EDTA (2) | 3.9 | 1.3 | 19 | 2 | 0 | 1.3 | 2006 | 8.6 | NA |
| 2 | Bokan 2006 | EMD+24%EDTA+Cerasorb(TCP) (3) | 4.1 | 1.2 | 19 | 3 | 0 | 1.2 | 2006 | 8.6 | NA |
| 3 | Döri 2008b | EMD+24%EDTA+Bio-Oss (3) | 5.9 | 1.3 | 13 | 3 | 0 | 1.3 | 2008 | 8.8 | 5.2 |
| 3 | Döri 2008b | EMD+24%EDTA+Bio-Oss+PRP (4) | 5.8 | 1.8 | 13 | 4 | 0 | 1.8 | 2008 | 8.8 | 5.1 |
| 4 | Francetti 2004 | Flap op (1) | 2.57 | 1.27 | 12 | 1 | 0 | 1.27 | 2004 | 6.71 | 4.81 |
| 4 | Francetti 2004 | EMD+24%EDTA (2) | 4.71 | 1.6 | 12 | 2 | 0 | 1.6 | 2004 | 7.86 | 5.93 |
| 5 | Francetti 2005 | Flap op (1) | 3 | 1.47 | 46 | 1 | 0 | 1.47 | 2005 | 7.11 | NA |
| 5 | Francetti 2005 | EMD+24%EDTA (2) | 4 | 2.01 | 64 | 2 | 0 | 2.01 | 2005 | 8.06 | NA |
| 6 | Minabe 2002 | EMD+37%orthophosphoric acid gel+GTR(Tissue Guide) (6) | 4.3 | 1.6 | 21 | 6 | 0 | 1.6 | 2002 | 7.9 | 5.7 |
| 6 | Minabe 2002 | EMD+37%orthophosphoric acid gel (2) | 3.8 | 0.9 | 17 | 2 | 0 | 0.9 | 2002 | 7.8 | 5.4 |
| 6 | Minabe 2002 | GTR(Tissue Guide) (5) | 3.7 | 1.2 | 23 | 5 | 0 | 1.2 | 2002 | 7 | 5.8 |
| 7 | Sculean 2005b | EMD+24%EDTA (2) | 4.5 | 2 | 15 | 2 | 0 | 2 | 2005 | 8.5 | 4.1 |
| 7 | Sculean 2005b | EMD+24%EDTA+Perioglass (3) | 4.2 | 1.4 | 15 | 3 | 0 | 1.4 | 2005 | 8.5 | 4.3 |
| 8 | Silvestri 2000 | Flap op (1) | 1.4 | 1.26 | 10 | 1 | 0 | 1.26 | 2000 | 7.7 | 5.2 |
| 8 | Silvestri 2000 | EMD+17%EDTA (2) | 4.9 | 1.79 | 10 | 2 | 0 | 1.79 | 2000 | 7.7 | 5.9 |
| 8 | Silvestri 2000 | GTR(ePTFE) (5) | 5.7 | 1.06 | 10 | 5 | 0 | 1.06 | 2000 | 8.1 | 4.6 |
| 9 | Tonetti 2002 | Flap op+24%EDTA (1) | 3.3 | 1.7 | 83 | 1 | 0 | 1.7 | 2002 | 7.7 | 5.4 |
| 9 | Tonetti 2002 | EMD+24%EDTA (2) | 3.9 | 1.7 | 83 | 2 | 0 | 1.7 | 2002 | 8 | 5.8 |
| 10 | Zucchelli 2002 | Flap op+24%EDTA (1) | 4.5 | 1 | 30 | 1 | 0 | 1 | 2002 | 8.9 | 6.2 |
| 10 | Zucchelli 2002 | EMD+24%EDTA (2) | 5.1 | 0.7 | 30 | 2 | 0 | 0.7 | 2002 | 9.2 | 6.1 |
| 10 | Zucchelli 2002 | GTR(Ti-ePTFE) (5) | 6.5 | 1.6 | 30 | 5 | 0 | 1.6 | 2002 | 8.9 | 6.6 |
| 11 | Zucchelli 2003 | EMD+24%EDTA (2) | 5.8 | 0.8 | 30 | 2 | 0 | 0.8 | 2003 | 9.2 | 6.8 |
| 11 | Zucchelli 2003 | EMD+24%EDTA+Bio-Oss (3) | 6.2 | 0.4 | 30 | 3 | 0 | 0.4 | 2003 | 9.4 | 6.7 |
| 12 | Guida 2007 | EMD+24%EDTA (2) | 5.6 | 1.7 | 14 | 2 | 0 | 1.7 | 2007 | 9.6 | 6.2 |
| 12 | Guida 2007 | EMD+24%EDTA+Autogenous bone grafts (3) | 5.1 | 1.7 | 13 | 3 | 0 | 1.7 | 2007 | 9.1 | 7 |
| 13 | Grusovin 2009 | Flap op (1) | 3.9 | 1 | 15 | 1 | 0 | 1 | 2009 | 8.5 | 5.1 |
| 13 | Grusovin 2009 | EMD+24%EDTA (2) | 4.2 | 1.6 | 15 | 2 | 0 | 1.6 | 2009 | 8.7 | 6.1 |
| 14 | Yilmaz 2010 | EMD+24%EDTA (2) | 4.6 | 0.4 | 20 | 2 | 0 | 0.4 | 2010 | 8.2 | 5.2 |
| 14 | Yilmaz 2010 | EMD+24%EDTA+Autogenous bone grafts (3) | 5.6 | 0.9 | 20 | 3 | 0 | 0.9 | 2010 | 8.4 | 5.4 |
| 15 | Crea 2008 | EMD+24%EDTA (2) | 3.2 | 1.3 | 19 | 2 | 0 | 1.3 | 2008 | 6.6 | 4.7 |
| 15 | Crea 2008 | GTR(ePTFE) (5) | 3.7 | 1.2 | 20 | 5 | 0 | 1.2 | 2008 | 7.2 | 4.9 |
| 16 | Silvestri 2003 | EMD+24%EDTA (2) | 5.3 | 1.9 | 48 | 2 | 0 | 1.9 | 2003 | 8.5 | 6.4 |
| 16 | Silvestri 2003 | GTR(ePTFE) (5) | 5.6 | 1.5 | 48 | 5 | 0 | 1.5 | 2003 | 8.1 | 6.1 |
| 17 | Sanz 2004 | EMD+24%EDTA (2) | 3.8 | 1.5 | 35 | 2 | 0 | 1.5 | 2004 | 7.9 | 6.2 |
| 17 | Sanz 2004 | GTR(Guidor) (5) | 3.3 | 1.5 | 32 | 5 | 0 | 1.5 | 2004 | 8 | 5.9 |
| 18 | Tonetti 2004 | Flap op (1) | 3.2 | 1.5 | 62 | 1 | 0 | 1.5 | 2004 | 7.9 | 5.9 |
| 18 | Tonetti 2004 | GTR(Bio-Gide)+Bio-Oss (7) | 3.7 | 1.8 | 62 | 7 | 0 | 1.8 | 2004 | 7.8 | 5.6 |
| 19 | Cortellini 2001 | Flap op (1) | 3.6 | 2.1 | 54 | 1 | 0 | 2.1 | 2001 | 8.2 | 6.3 |
| 19 | Cortellini 2001 | GTR(Guidor) (5) | 4.4 | 2.4 | 55 | 5 | 0 | 2.4 | 2001 | 8.2 | 6.3 |
| 20 | Mayfield 1998 | Flap op (1) | 2.5 | 1.9 | 18 | 1 | 0 | 1.9 | 1998 | 7.8 | 6.6 |
| 20 | Mayfield 1998 | GTR(Guidor) (5) | 2.9 | 1.8 | 20 | 5 | 0 | 1.8 | 1998 | 8.2 | 6.9 |
| 21 | Tonetti 1998 | Flap op (1) | 3.09 | 1.67 | 71 | 1 | 0 | 1.67 | 1998 | 7.3 | 5.3 |
| 21 | Tonetti 1998 | GTR(Resolut) (5) | 4.03 | 1.81 | 72 | 5 | 0 | 1.81 | 1998 | 8.3 | 5.9 |
| 22 | Dori 2007b | GTR(ePTFE)+Bio-Oss (7) | 5.7 | 1.2 | 12 | 7 | 0 | 1.2 | 2007 | 8.8 | 5.2 |
| 22 | Dori 2007b | GTR(ePTFE)+Bio-Oss+PRP (8) | 5.5 | 1.2 | 12 | 8 | 0 | 1.2 | 2007 | 8.6 | 5.1 |
| 23 | Dori 2008a | GTR(ePTFE)+TCP (7) | 5.4 | 0.7 | 14 | 7 | 0 | 0.7 | 2008 | 9 | 5.2 |
| 23 | Dori 2008a | GTR(ePTFE)+TCP+PRP (8) | 5.8 | 0.6 | 14 | 8 | 0 | 0.6 | 2008 | 9.1 | 5.3 |
| 24 | Dori 2007a | GTR(ePTFE)+Bio-Oss (7) | 5.5 | 1.7 | 15 | 7 | 0 | 1.7 | 2007 | 8.8 | 5 |
| 24 | Dori 2007a | GTR(ePTFE)+Bio-Oss+PRP (8) | 5.5 | 1.3 | 15 | 8 | 0 | 1.3 | 2007 | 8.7 | 4.9 |
| 25 | Sculean 2005a | Flap op (1) | 3.6 | 1.3 | 16 | 1 | 0 | 1.3 | 2005 | 8 | 3.7 |
| 25 | Sculean 2005a | GTR(Bio-Gide)+Bio-Oss (7) | 5.4 | 0.9 | 16 | 7 | 0 | 0.9 | 2005 | 8.3 | 3.8 |
| 26 | Sculean 2003 | Flap op (1) | 3.8 | 1.8 | 14 | 1 | 0 | 1.8 | 2003 | 9 | 4.4 |
| 26 | Sculean 2003 | GTR(Bio-Gide)+Bio-Oss (7) | 5.3 | 1.6 | 14 | 7 | 0 | 1.6 | 2003 | 9.2 | 4.1 |
| 27 | Trejo 2000 | GTR(Guidor) (5) | 4.12 | 0.84 | 14 | 5 | 0 | 0.84 | 2000 | 7.93 | NA |
| 27 | Trejo 2000 | GTR(Guidor)+DFDBA (7) | 3.37 | 1.16 | 16 | 7 | 0 | 1.16 | 2000 | 7.34 | NA |
| 28 | Paolantonio 2008 | Flap op (1) | 2.8 | 0.73 | 17 | 1 | 0 | 0.73 | 2008 | 7.2 | NA |
| 28 | Paolantonio 2008 | GTR(Collagen) (5) | 5.2 | 1.09 | 17 | 5 | 0 | 1.09 | 2008 | 8 | NA |
| 29 | Cortellini 1996 | Flap op (1) | 4.3 | 1.73 | 12 | 1 | 0 | 1.7349 | 1996 | 8.5 | 6.7 |
| 29 | Cortellini 1996 | GTR(ePTFE);GTR(Resolute) (5) | 6.2 | 1.72 | 24 | 5 | 0 | 1.72 | 1996 | 9.2 | 7.1 |
| 30 | Cortellini 1995 | Flap op (1) | 4.6 | 1.76 | 15 | 1 | 0 | 1.76 | 1995 | 8.3 | 5.5 |
| 30 | Cortellini 1995 | GTR(ePTFE);GTR(Ti-ePTFE) (5) | 5.9 | 2.19 | 30 | 5 | 0 | 2.1879 | 1995 | 8.3 | 5.65 |
| 31 | Stavropoulos 2003 | Flap op (1) | 2.9 | 1.74 | 15 | 1 | 0 | 1.74 | 2003 | 7.9 | NA |
| 31 | Stavropoulos 2003 | GTR(Resolut-XT) (5) | 3.9 | 1.56 | 14 | 5 | 0 | 1.56 | 2003 | 8.9 | NA |
| 31 | Stavropoulos 2003 | GTR(Resolut-XT)+Bio-Oss (7) | 3.8 | 1.52 | 15 | 7 | 0 | 1.52 | 2003 | 8.7 | NA |
| 32 | Cortellini 2011 | Flap op (1) | 4.4 | 1.6 | 15 | 1 | 0 | 1.6 | 2011 | 7.5 | 5.2 |
| 32 | Cortellini 2011 | EMD+24%EDTA (2) | 4.4 | 1.2 | 15 | 2 | 0 | 1.2 | 2011 | 7.8 | 5.3 |
| 32 | Cortellini 2011 | EMD+24%EDTA+Bio-Oss (3) | 4 | 1.3 | 15 | 3 | 0 | 1.3 | 2011 | 7.3 | 5.2 |
| 33 | Siciliano 2011 | EMD+24%EDTA (2) | 2.9 | 2.1 | 20 | 2 | 0 | 2.1 | 2011 | 9.4 | 8.5 |
| 33 | Siciliano 2011 | GTR(Ti-ePTFE) (5) | 5.5 | 1 | 20 | 5 | 0 | 1 | 2011 | 9.6 | 8.6 |
| 34 | Meyle 2011 | EMD+24%EDTA (2) | 2.9 | 1.8 | 35 | 2 | 0 | 1.8 | 2011 | 7.1 | 5.6 |
| 34 | Meyle 2011 | EMD+24%EDTA+Bone Ceramic (3) | 2.8 | 2.1 | 38 | 3 | 0 | 2.1 | 2011 | 6.9 | 5.9 |
| 35 | Blumenthal 1990 | Flap op (1) | 1.51 | 0.63 | 10 | 1 | 1 | 0.567186 | 1990 | NA | NA |
| 35 | Blumenthal 1990 | GTR(Collagen) (5) | 1.99 | 0.32 | 10 | 5 | 1 | 0.277128 | 1990 | NA | NA |
| 35 | Blumenthal 1990 | GTR(Collagen)+DFDBA (7) | 2.73 | 0.32 | 10 | 7 | 1 | 0.277128 | 1990 | NA | NA |
| 36 | Sipos 2005 | EMD+24%EDTA (2) | 2.86 | 0.75 | 11 | 2 | 1 | 0.521416 | 2005 | 6.95 | 4.3 |
| 36 | Sipos 2005 | EMD+24%EDTA+GTR(ePTFE) (6) | 3.02 | 1.55 | 11 | 6 | 1 | 1.453229 | 2005 | 7.32 | 4.3 |
| 37 | Froum 2001 | Flap op (1) | 2.24 | 0.91 | 23 | 1 | 1 | 0.823377 | 2001 | 7.32 | NA |
| 37 | Froum 2001 | EMD+citric acid (2) | 4.94 | 0.66 | 23 | 2 | 1 | 0.534275 | 2001 | 7.99 | NA |
| 38 | Heijl 1997* | Flap op+PGA (1) | 2.6 | 1.2 | 30 | 1 | 1 | 1.00995 | 1997 | 7.8 | 6.5 |
| 38 | Heijl 1997* | EMD (2) | 3.3 | 1.4 | 30 | 2 | 1 | 1.240967 | 1997 | 7.8 | 7.1 |
| 39 | Okuda 2000 | Flap op+PGA+37%orthophosphoric acid gel (1) | 2.22 | 0.81 | 16 | 1 | 1 | 0.677993 | 2000 | 6.22 | NA |
| 39 | Okuda 2000 | EMD+37%orthophosphoric acid gel (2) | 3 | 0.97 | 16 | 2 | 1 | 0.86283 | 2000 | 6.33 | NA |
| 40 | Pontoriero 1999a | Flap op+PGA (1) | 3.5 | 1.25 | 10 | 1 | 1 | 1.111024 | 1999 | 7.9 | 4.3 |
| 40 | Pontoriero 1999a | EMD+24%EDTA (2) | 4.4 | 1.05 | 10 | 2 | 1 | 0.879986 | 1999 | 8 | 4.2 |
| 41 | Rosing 2005 | Flap op+24%EDTA (1) | 4.39 | 1.5 | 14 | 1 | 1 | 1.285982 | 2005 | 7.38 | NA |
| 41 | Rosing 2005 | EMD+24%EDTA (2) | 4.17 | 1.59 | 14 | 2 | 1 | 1.38991 | 2005 | 7.57 | NA |
| 42 | Chambrone 2010 | Flap op (1) | 3.49 | 1.18 | 10 | 1 | 1 | 1.041418 | 2010 | 6.13 | NA |
| 42 | Chambrone 2010 | EMD+24%EDTA (2) | 4 | 0.99 | 9 | 2 | 1 | 0.838473 | 2010 | 6.3 | NA |
| 43 | Loos 2002 | Flap op (1) | 2.31 | 1.64 | 25 | 1 | 1 | 1.412941 | 2002 | 7.404 | 5.3 |
| 43 | Loos 2002 | GTR(Guidor) (5) | 2.41 | 1.67 | 25 | 5 | 1 | 1.450816 | 2002 | 7.504 | 5.32 |
| 44 | Pritlove-Carson 1995 | Flap op (1) | 1.44 | 1.29 | 20 | 1 | 1 | 1.063943 | 1995 | 6.68 | NA |
| 44 | Pritlove-Carson 1995 | GTR(ePTFE) (5) | 2.67 | 1.65 | 20 | 5 | 1 | 1.479992 | 1995 | 6.68 | NA |
| 45 | Ratka-Kruger 2000 | Flap op (1) | 4.45 | 2.45 | 11 | 1 | 1 | 2.20944 | 2000 | 8.6 | 6 |
| 45 | Ratka-Kruger 2000 | GTR(Guidor) (5) | 4.23 | 1.83 | 11 | 5 | 1 | 1.492657 | 2000 | 8.4 | 6.8 |
| 46 | Cortellini 1998 | Flap op (1) | 3 | 1.5 | 23 | 1 | 1 | 1.177922 | 1998 | 6.7 | 3.9 |
| 46 | Cortellini 1998 | GTR(Guidor) (5) | 4.3 | 2.3 | 23 | 5 | 1 | 2.104163 | 1998 | 7.4 | 4 |
| 48 | Christau 2006 | GTR(Resolut XT)+24%EDTA+TCP (7) | 6 | 1.1 | 25 | 7 | 1 | 0.938083 | 2006 | 9.9 | NA |
| 48 | Christau 2006 | GTR(Resolut XT) +24%EDTA+TCP+PRP (8) | 6.3 | 1.2 | 25 | 8 | 1 | 1.053565 | 2006 | 9.9 | NA |
| 50 | Pontoriero 1999b | Flap op (1) | 3.4 | 1.41 | 30 | 1 | 1 | 1.249384 | 1999 | 7.4 | NA |
| 50 | Pontoriero 1999b | GTR(Guidor, Resolut, ePTFE) (5) | 4.5 | 1.22 | 30 | 5 | 1 | 1.025163 | 1999 | 7.8 | NA |
| 51 | Mora 1996 | Flap op (1) | 3.55 | 1.1 | 10 | 1 | 1 | 0.923309 | 1996 | 7.8 | 3.85 |
| 51 | Mora 1996 | GTR(ePTFE) (5) | 5.35 | 1.3 | 10 | 5 | 1 | 1.15434 | 1996 | 7.95 | 4.35 |
| 52 | Fickl 2009 | Flap op (1) | 2.4 | 1.31 | 19 | 1 | 1 | 1.132415 | 2009 | NA | NA |
| 52 | Fickl 2009 | EMD+24%EDTA (2) | 4.2 | 1.31 | 19 | 2 | 1 | 1.132415 | 2009 | NA | NA |
| 53 | DiBattista 1995 | GTR(ePTFE) & GTR(ePTFE) +Doxycyclin (5) | 3.85 | 1.52 | 14 | 5 | 1 | 1.062129 | 1995 | 9.75 | NA |
| 53 | DiBattista 1995 | GTR(ePTFE)+Doxycyclin+DFDBA (7) | 4.3 | 2.2 | 7 | 7 | 1 | 2.061276 | 1995 | 10.6 | NA |
| 54 | Pietruska 2012** | EMD (2) | 4.7 | 0.87 | 12 | 2 | 0 | 0.87 | 2012 | 8.8 | NA |
| 54 | Pietruska 2012** | EMD+BG(biphasic calcium phosphate) (3) | 4.5 | 1.31 | 12 | 3 | 0 | 1.31 | 2012 | 8.8 | NA |
| 55 | Bhutda 2013 | Flap op+24%EDTA (1) | 2.22 | 0.15 | 15 | 1 | 1 | 0.076485 | 2013 | 6.82 | NA |
| 55 | Bhutda 2013 | Flap op+EDTA(24%)+EMD (2) | 4.12 | 1.11 | 15 | 2 | 1 | 1.091089 | 2013 | 7.24 | NA |
| 56 | Leonardis 2013 | Flap op (1) | 2.58 | 0.55 | 34 | 1 | 1 | 0.475289 | 2013 | 8.7 | 4.31 |
| 56 | Leonardis 2013 | Flap op+EMD (2) | 3.51 | 0.58 | 34 | 2 | 1 | 0.503488 | 2013 | 8.73 | 4.55 |
| 56 | Leonardis 2013 | Flap op+EMD+BG(HA/b-TCP) (3) | 4 | 0.42 | 34 | 3 | 1 | 0.37081 | 2013 | 8.79 | 4.49 |
| 57 | Ogihara 2014 | EMD (2) | 3.26 | 0.73 | 23 | 2 | 0 | 0.73 | 2014 | 6.56 | NA |
| 57 | Ogihara 2014 | EMD+BG(FDBA or DFDBA) (3)*** | 4.02 | 0.86 | 44 | 3 | 0 | 0.86 | 2014 | 6.5 | NA |
| 58 | Ghezzi 2016 | MIST+IPR(inductive periodontal regeneration) (IPR+EMD+DBBM) (3) | 4.9 | 1.2 | 10 | 3 | 0 | 1.2 | 2016 | 8.2 | 5.6 |
| 58 | Ghezzi 2016 | MIST(minimal invasive surgical technique)+GTR (GTR+DBBM) (7) | 4.7 | 2.36 | 10 | 7 | 0 | 2.36 | 2016 | 7.8 | 5.4 |
| 59 | Iorio-Siciliano 2014 | EMD+DBBM+(24%EDTA gel) (3) | 4.6 | 1.9 | 20 | 3 | 0 | 1.9 | 2014 | 8.2 | 6.1 |
| 59 | Iorio-Siciliano 2014 | GTR(Collagen membrane)+DBBM (7) | 4.4 | 1.7 | 20 | 7 | 0 | 1.7 | 2014 | 8.1 | 6 |
| 60 | Losada 2017 | EMD (2) | 3.3 | 1.89 | 23 | 2 | 0 | 1.89 | 2017 | 7.88 | 5.24 |
| 60 | Losada 2017 | EMD/BC (biphasic calcium phosphate) (3) | 3.14 | 1.95 | 21 | 3 | 0 | 1.95 | 2017 | 7.95 | 4.66 |
| 61 | Moreno Rodríguez 2022 | EMD+BG (Xenograft) (3) | 6.83 | 0.81 | 12 | 3 | 0 | 0.81 | 2022 | 9.5 | 6.08 |
| 61 | Moreno Rodríguez 2022 | EMD (2) | 8.25 | 2.7 | 12 | 2 | 0 | 2.7 | 2022 | 10.75 | 5.92 |
| 62 | Aslan 2020 | EMD+BG (Xenograft) (3) | 6.5 | 2.65 | 15 | 3 | 0 | 2.65 | 2020 | 9.33 | 6.63 |
| 62 | Aslan 2020 | Flap op (1) | 6.2 | 1.33 | 15 | 1 | 0 | 1.33 | 2020 | 9.26 | 6.7 |
| 63 | Fileto Mazzonetto 2020 | EMD (2) | 2.3 | 1.2 | 20 | 2 | 0 | 1.2 | 2020 | 6.7 | 5.64 |
| 63 | Fileto Mazzonetto 2020 | Flap op (1) | 1.9 | 1.5 | 20 | 1 | 0 | 1.5 | 2020 | 6.7 | 5.53 |
| 64 | Gomez-Monchero 2025 | EMD (2) | 4.9 | 1.440919 | 19 | 2 | 0 | 1.440919 | 2025 | 8.15 | 5.35 |
| 64 | Gomez-Monchero 2025 | EMD+BG (Xenograft) (3) | 5.21 | 1.024694 | 21 | 3 | 0 | 1.024694 | 2025 | 8.5 | 5.04 |
| 65 | Liu 2022 | Flap op (1) | 2.5 | 1.22 | 15 | 1 | 0 | 1.22 | 2022 | 6.63 | 5.33 |
| 65 | Liu 2022 | GTR(Biogide)+BG (Bio-Oss) (7) | 2.31 | 1.47 | 16 | 7 | 0 | 1.47 | 2022 | 6.63 | 4.72 |
| 66 | Liu 2021 | GTR(Collagen)+BG+PRP (8) | 3.05 | 0.93675 | 14 | 8 | 1 | 0.794735 | 2021 | 5.3 | 4.8 |
| 66 | Liu 2021 | GTR(Collagen)+BG (7) | 2.6 | 1.05 | 14 | 7 | 1 | 0.925529 | 2021 | 5.4 | 4.4 |
| 67 | Vu Pham 2021 | GTR (resorbable) (5) | 4.63 | 0.67 | 30 | 5 | 1 | 0.530471 | 2021 | 7.6 | NA |
| 67 | Vu Pham 2021 | Flap op (1) | 3.37 | 1 | 30 | 1 | 1 | 0.912414 | 2021 | 7.4 | NA |

**R script for network meta-analysis**

```
## Step 1: Read data
## read data from Excel into R
## install.packages("readxl")
library("readxl")
PPD_long<-read_excel("Periodontal_Regeneration_JPR.xlsx",
                     sheet = "PPD", na = "NA")

## Make R package netmeta available
library(netmeta)

## Label treatments
PPD_long$txt<-as.factor(PPD_long$t)

## Rename all levels of txt
levels(PPD_long$txt) <- c("FO", "EMD", "EMD+BG", "EMD+PRP+BG",
                               "GTR", "GTR+EMD", "GTR+BG", "GTR+PRP+BG")

## Relevel the order of treatments according to alphabet
PPD_long$txt <- factor(PPD_long$txt,
                       levels = c("EMD", "EMD+BG", "EMD+PRP+BG",
                                  "FO", "GTR", "GTR+BG", "GTR+EMD", "GTR+PRP+BG"))

## Define order of treatments in printouts and forest plots
## Do not use trts!
txts <- c("FO", "EMD", "EMD+BG", "EMD+PRP+BG",
          "GTR", "GTR+EMD", "GTR+BG", "GTR+PRP+BG")

## Define the variable for study design
PPD_long$group <- factor(PPD_long$g, levels = 0:1,
                       labels = c("Parallel", "Split-mouth"))

## Step 2: Prepare the data
## Transforming data
PPD_wide <- pairwise(treat=txt, mean=y, sd=sd, n=n,
                     studlab=study, data=PPD_long, sm="md")

## Step 3: fit random effects NMA
## Standard random effects NMA model (with FO as reference treatment)
net1 <- netmeta(PPD_wide, reference.group = "FO",
                sm = "md", common = FALSE, random = TRUE,
                nchar.trts = 18, seq=txts)

net1

netgraph(net1)

## Step 4: Create a network plot
## network graphs looking like those in Stata
n.subj<-PPD_long$n
n.txts<-PPD_long$txt
netsize<-data.frame(n.txts, n.subj)
pointsize<-aggregate(n.subj ~ factor(n.txts), data=netsize, FUN=sum)

pdf(file="networkplot.pdf",width=10,height=8,paper='special')
# create a transparent colour
mycol <- rgb(0, 0, 255, max = 255, alpha = 0, names = "blue0")
netgraph(net1, points=TRUE, multiarm=FALSE, plastic=FALSE,
         scale=1.1, cex.points=pointsize$n.subj/60, cex=1.5,
         number.of.studies=TRUE, thickness="number.of.studies",
         pos.number.of.studies=0.3, col.number.of.studies="black",
         bg.number.of.studies=mycol, offset=0.05,
         cex.number.of.studies=1.1, col="lightblue",
         col.points="steelblue")

dev.off()

## Step 5: Create a forest plot
## Create forest plot according to effect size
forest(net1)

pdf(file="forestplot.pdf",width=6,height=4,paper='special')
forest(net1,reference.group = "FO",sortvar = -TE,xlim=c(-1,3),
       at=c(-1,-0.5,0,0.5,1,1.5,2,2.5,3),
       smlab = "Regerative Therapies vs. Flap Operation \n (PPD reduction)")
dev.off()


## Step 6: Treatment ranking
## treatment ranking
ranking<-netrank(net1, small.values = "bad")
ranking
set.seed(1909) # get reproducible results
ran1 <- rankogram(net1, nsim = 1000, small.values = "undesirable")
ran1
print(ran1, cumulative.rankprob = TRUE)
pdf(file="ranking_prob_plot.pdf",width=8,height=5,paper='special')
plot(ran1, type="l")
dev.off()
pdf(file="cumul_prob_plot.pdf",width=8,height=5,paper='special')
plot(ran1, type="l", cumulative.rankprob = TRUE)
dev.off()

## Step 7: League table
## Create league table
## League table for random effects model with
## - network estimates in lower triangle
## - direct estimates in upper triangle
## - change order of treatments according to treatment ranking
netleague <- netleague(net1, common = FALSE,
                       bracket = "(", separator = " to ", digits=2,
                       seq = netrank(net1, small.values = "bad"))
write.csv(netleague$random, "netleague.csv")


## Step 8: Funnel plot
## Comparison-adjusted Funnel Plots
ranking.r<-as.data.frame(ranking$Pscore.random)
names(ranking.r)<-"pscore"
ranking.r$trtnames<-rownames(ranking.r)
ranking.order<-ranking.r[order(ranking.r$pscore, decreasing = TRUE),]

pdf(file="funnelplot.pdf",width=10,height=8,paper='special')
funnel(net1,order = ranking.order$trtnames,pch = 19,
legend=FALSE, method.bias = "Egger",studlab = TRUE,cex.studlab = 0.7)
dev.off()

## Step 9: Global inconsistency
## Design-by-treatment interaction model
decomp.design(net1)

## Step 10: Local inconsistency
## node-splitting model
netsplit(net1)

pdf(file="nodesplit.pdf",width=8,height=13,paper='special')

forest(netsplit(net1))

dev.off()

## Step 11: violin plot for the distribution of baseline PPD
PPD_wide$ave.bppd <- (PPD_wide$base.PPD1 + PPD_wide$base.PPD2)/2
PPD_wide$ave.intra.def <- (PPD_wide$base.intra.def1 + PPD_wide$base.intra.def2)/2
PPD_wide$design <- paste(PPD_wide$treat1, PPD_wide$treat2, sep = " vs ")
# Basic violin plot
library(ggplot2)
library(gridExtra)
pdf(file="Violinplot for baseline ppd & intra.pdf",width=8,height=10)

p1 <- ggplot(PPD_wide, aes(x=design, y=ave.bppd)) + geom_violin() +
  geom_dotplot(binaxis='y', stackdir='center', dotsize=0.5) +
  theme(
  axis.text.x = element_text(angle = 45, hjust = 1, vjust = 1, size=6)
  )

p2 <- ggplot(PPD_wide, aes(x=design, y=ave.intra.def)) + geom_violin() +
  geom_dotplot(binaxis='y', stackdir='center', dotsize=0.5) +
  theme(
    axis.text.x = element_text(angle = 45, hjust = 1, vjust = 1, size=6)
  )

p1 <- p1 +
  labs(
    title = "(A) Average baseline PPD by direct comparisons",
    x = "Direct comparisons",
    y = "Average baseline PPD (mm)"
  ) +
  theme(
    plot.title = element_text(
      hjust = 0.5,
      size = 14,
      face = "bold"
    )
  )

p2 <- p2 +
  labs(
    title = "(B) Average intrabony defect depth by direct comparisons",
    x = "Direct comparisons",
    y = "Average intrabony defect depths (mm)"
  ) +
  theme(
    plot.title = element_text(
      hjust = 0.5,
      size = 14,
      face = "bold"
    )
  )

grid.arrange(p1, p2, nrow = 2)

dev.off()

## Step 11: Subgroup analysis and Meta-regression
# group (parallel group versus split-mouth design)
# Network meta-regression with a continuous covariate and assumption of
# independent slopes

# Conduct subgroup network meta-analysis
sub.net1<-subgroup(net1, group, common = FALSE)
sub.net1

pdf(file="subgroup_forest.pdf",width=8,height=20,paper='special')
forest(sub.net1)
dev.off()

sub.net1$TE.random

nr1 <- netmetareg(net1, group)
nr1
summary(nr1)
```
