# Supplementary material for: A Practical Guide to Evidence Synthesis: Meta‐Analysis, Network Meta‐Analysis, Meta‐Regressions and Beyond
Source: J Periodontal Res. 2026 Jul 25;61(7):847–75. doi: 10.1111/jre.70150 (PMC13665622; doi:10.1111/jre.70150)
Supplement: Supplementary file 5 [file JRE-61-847-s005.docx]

**Supplementary Table S3**: Quality Assessment of included RCTs for network meta-analysis

| **Study** | **Adequate sequence generation?** | **Allocation concealment?** | **Blinding?** | **Incomplete outcome data addressed?** | **Free of selective reporting?** | **Risk of bias** |
| --- | --- | --- | --- | --- | --- | --- |
| 1 | Unclear | Unclear | Unclear | Yes | Yes | Unclear |
| 2 | Unclear | Unclear | Unclear | Yes | Yes@ | Unclear |
| 3 | Yes | Unclear | Yes | Yes | Yes | Unclear |
| 4 | Unclear | Unclear | Unclear | Yes | Yes | Unclear |
| 5 | Yes | Unclear | Unclear | Yes | Yes | Unclear |
| 6 | Yes | No | Unclear | Yes | Yes | High |
| 7 | Yes | Unclear | Yes | Yes | Yes | Unclear |
| 8 | Unclear | Unclear | Unclear | Yes | Yes | Unclear |
| 9 | Yes | Unclear* | Yes | Yes= | Yes | Unclear |
| 10 | Yes | Unclear* | Unclear | Yes | Yes | Unclear |
| 11 | Unclear | Unclear | Yes | Yes | Yes% | Unclear |
| 12 | Yes | Unclear* | Unclear | Yes= | Yes | Unclear |
| 13 | Unclear | Unclear | Unclear | Yes | Yes | Unclear |
| 14 | Yes | No§ | Yes | Yes | Yes | High |
| 15 | Unclear | Unclear | Unclear | Yes | Yes | Unclear |
| 16 | Unclear | Unclear | Unclear | Yes | Yes | Unclear |
| 17 | Yes | Unclear* | Yes | Yes= | Yes | Unclear |
| 18 | Yes | Yes | Unclear | Yes | Yes | Unclear |
| 19 | Yes | Unclear | Yes | Unclear¤ | Yes | Unclear |
| 20 | Yes | No | Yes | Yes | Yes | High |
| 21 | Yes | Unclear* | Yes | Yes | Yes | Unclear |
| 22 | Unclear | Unclear | Yes | Yes | Yes | Unclear |
| 23 | Yes | Yes | No | Yes | Yes | High |
| 24 | Yes | Unclear | Yes | Yes | Yes | Unclear |
| 25 | Unclear | Unclear | Unclearµ | Yes | Yes | Unclear |
| 26 | Yes | Unclear | Unclear | Yes= | Yes | Unclear |
| 27 | Yes | Unclear* | Yes | Yes= | Yes | Unclear |
| 28 | Yes | Unclear | Unclear | Yes | Yes# | Unclear |
| 29 | Yes | Unclear | Yes | Yes | Yes# | Unclear |
| 30 | Yes | Unclear | No | Unclear° | Yes | High |
| 31 | Yes | Yes | No | Yes= | Yes | High |
| 32 | Yes | Yes | Yes | Yes | Yes | Low |
| 33 | Yes | No | Yes | Yes | Yes | High |
| 34 | Yes | Unclear | Unclearµ | Yes | Yes | Unclear |
| 35 | Yes | Unclear | Yes | Yes | Yes | Unclear |
| 36 | Yes | Unclear | Yes | Yes^ | Yes | Unclear |
| 37 | Yes | Unclear | Yes | Yes | Yes¶ | Unclear |
| 38 | Yes | Yes | Yes | Yes | Yes | Low |
| 39 | Unclear | Unclear | Yes | Yes | Yes | Unclear |
| 40 | Yes | Unclear | Yes | Yes | Yes | Unclear |
| 41 | Yes | Unclear | Yes | Yes | Yes | Unclear |
| 42 | Unclear | Unclear | No | Yes | Yes | High |
| 43 | Yes | Unclear* | Yes | Yes | Yes | Unclear |
| 44 | Yes | Unclear | Yes | Yes | Yes | Unclear |
| 45 | Yes | Unclear | Yes | Yes | Yes | Unclear |
| 46 | Yes | Unclear | Yes& | Yes | Yes | Unclear |
| 47 | Yes | No | Yes | Yes | Yes | High |
| 48 | Yes | Yes | Yes | Yes | Yes= | Low |
| 49 | Yes | No | Yes | Yes | Yes | High |
| 50 | Yes | No | Yes | Yes | Yes | High |
| 51 | Yes | Yes | Unclear | Yes | Yes | Unclear |
| 52 | Yes | Unclear* | Yes | Yes | Yes | Unclear |
| 53 | Unclear | Unclear | Yes | Yes | Yes | Unclear |
| 54 | Yes | Unclear | Unclear | Yes | Yes | Unclear |
| 55 | Yes | Unclear | Unclear | Yes | Yes | Unclear |
| 56 | Yes | Yes | Unclear | Yes | Yes | Unclear |
| 57 | Yes | Yes | Unclear | Yes | Yes | Unclear |
| 58 | Yes | Yes | Unclear | Yes | Yes | Unclear |
| 59 | Yes | Yes | Yes | Yes | Yes | Low |
| 60 | Yes | Yes | Yes | Unclear | Yes | Unclear |
| 61 | Low | Low | Low | Low | Low | Low |
| 62 | Low | Low | Low | Low | Low | Low |
| 63 | Low | Low | Low | Low | Low | Low |
| 64 | Unclear | Low | Low | Low | Unclear | Unclear |
| 65 | Low | Low | Low | Low | Unclear | Unclear |
| 66 | Low | Low | Low | Low | Low | Low |
| 67 | Low | Low | Low | Low | Unclear | Unclear |
